# Trial-by-trial fMRI-neurofeedback dissociates fusiform and occipital contributions to face detection and recognition

**DOI:** 10.1038/s41467-026-74331-2

**Published:** 2026-06-13

**Authors:** Lucas Peek, Yury Koush, Patrik Vuilleumier

**Affiliations:** 1 Laboratory for Behavioural Neurology and Imaging of Cognition, Department of Basic Neuroscience, University of Geneva, Geneva, Switzerland; 2 Center for Bio- and Medical Technologies, Moscow, Russia; 3 Swiss Center for Affective Science, University of Geneva, Geneva, Switzerland; 4 Center for Biomedical Imaging, University of Geneva/EPFL, Lausanne, Switzerland

**Keywords:** Extrastriate cortex, Perception

## Abstract

Self-regulation of specific brain regions can be achieved using neurofeedback with real-time functional magnetic resonance imaging (rt-fMRI). We leveraged this technique to dissect the role of two tightly interconnected areas implicated in face perception, by interleaving a visual task with upregulation of either the occipital (OFA) or fusiform face-responsive areas (FFA) in a trial-wise manner. Experimental participants (*N* = 22) successfully enhanced their target region when compared to yoked controls (*N* = 20). Regulation was face-selective, as evidenced by concomitant increases in other nodes of the face processing network. Critically, face detection was faster with enhanced FFA activity but hindered by enhanced OFA, whereas face identity recognition was optimal with concomitant increases in both FFA and OFA. These results argue against traditional face processing models assuming an information flow from posterior occipital to anterior fusiform cortex and instead support non-hierarchical models where FFA mediates initial face detection and OFA contributes to subsequent identity recognition.

## Abstract (web summary)

Using real-time fMRI neurofeedback to selectively upregulate fusiform and occipital face areas, the authors show that FFA mediates face detection while OFA contributes to identity recognition, challenging hierarchical models of face processing.

## Introduction

Performing any perceptual or cognitive task requires recruiting the most appropriate brain areas in the most efficient manner for the task purpose—a neurobiological feat we achieve every day without thinking about which neurons in our brain must be turned on (or off) and how they must be coordinated. This effortless mastery may be disrupted by injury affecting critical nodes or connections within brain networks, sometimes resulting in very selective deficits in one particular domain, as seen, for example, in patients with prosopagnosia (a failure to recognize faces with no other visual impairment) or alexia (a failure to read words with no other language disorders) after stroke^1^. Our knowledge of functional brain networks in humans has been greatly expanded by mapping their activation during specific tasks, yet the exact role of many areas within these networks still remains unresolved.

Recent development in neuroimaging now allows real-time measures of activity that open new avenues to explore brain regions and networks. For example, functional magnetic resonance imaging (rt-fMRI) can be employed for neurofeedback (NFB) studies where individuals are given real-time information on their brain activity to learn self-regulating specific regions^2^. This has usually been applied to train participants to modulate average BOLD activity in a target area over repeated sessions to produce a sustained enhancement or attenuation of corresponding behaviours, such as pain perception^3^, anxiety^4^, emotion^5^, or visual attention^6,7^. Fine-grained voxel-wise activity patterns within a region can also be trained, e.g., in early visual cortex to induce stimulus-specific perceptual learning^8^.

Here, we leveraged rt-fMRI NFB in a targeted approach to separately manipulate global activity within two *different* cortical regions during the *same* visual task (across different trials), with the aim to influence their relative recruitment for optimal task performance and thus dissect their respective functional role.

### Hierarchical and non-hierarchical processing in the core face network

We deployed NFB to target two nearby visual areas implicated in face perception, the fusiform face area (FFA) and the occipital face area (OFA) and test their contribution to early stages of face processing, namely: face detection and face recognition. Both FFA^9–11^ and OFA^11–13^ play a key role in the visual analysis of faces^13–16^, together with the posterior superior temporal sulcus (pSTS) implicated in motion and social analysis^17,18^. However, despite intense research, fundamental disagreements remain concerning the computation and propagation of face information within this core network. Most notably, two models have been opposed to describe functional interactions between OFA and FFA, assuming either hierarchical or non-hierarchical relationships.

Following a conventional feed-forward, “local to global” organization of the visual system^19^, the *hierarchical model* posits that face information is processed with increasing complexity from posterior/occipital (lower order) to anterior/temporal (higher order) areas^13,15,20^. In this view, OFA is the first region to receive face-related inputs from early visual cortex and encodes local features (e.g., eyes, nose) that are subsequently integrated into a face configuration in higher-order regions, such as FFA. Accordingly, some authors have proposed that OFA encodes elementary face features necessary for face detection (i.e., categorizing faces from non-faces), which are combined in full faces at later stages^16,21^, and convey limited information about identity^12^

In both cases, information would propagate from OFA to FFA for finer analysis of face shape, eventually enabling *identity recognition*. In support of this view, OFA responses are larger for faces with internal features (eyes, nose, and mouth) than without, regardless of their placement^22^. In contrast, FFA is sensitive to both features and their correct placement^22^, pointing to a more general role in structural face encoding^16^. Finer configural analysis of feature position and metrics in FFA might be crucial for face identification^23^, in agreement with FFA recruitment during recognition or priming studies^24–26^ where a given identity is presented in visually different images^27^. Other studies reported that FFA activity correlates with variability in recognition performance across trials^9^ or individuals^28,29^. Time-resolved measures with magnetoencephalography (MEG) showed that face-evoked activity at 100 ms (M100) post-stimulus is sensitive to face features and predictive of face detection performance^29^, whereas later responses at 170 ms (M170) are tuned to face configuration and related to both face detection and recognition. These functional characteristics might accord with those of OFA and FFA, respectively. Indeed, a simultaneous fMRI-EEG study reported face-selective ERP responses localized in OFA at 110–120 ms, preceding face-selective ERPs in FFA around 150–180 ms^30^. Accordingly, in line with a hierarchical “local to global” account of visual perception, OFA could constitute the first entry point extracting face-specific inputs for basic visual computations (i.e., based on internal feature) that mediate an early face detection stage^12^ or encode detection-specific cues^31^ while FFA might be recruited later by more global computations needed for identity recognition.

Instead, a *non-hierarchical model* argues for a “global-to-local” scheme where face processing may *start* with a crude, global face representation in FFA^32,33^, underpinned by low spatial frequency information received from early visual cortex. This first processing step would constitute a domain-specific stimulus-categorization filter^33^ whereby FFA initially mediates *face detection*. Subsequently, additional neurons in lower-order regions such as OFA might operate through re-entrant signalling and “fill-in” local information to allow face *identity recognition*^32,33^. In line with this view, two-tone face images^34^, made of non-recognizable local elements, produce selective activation of FFA but *not* OFA when perceived as faces^35,36^. Moreover, the FFA still responds to faces after OFA damage in patients with prosopagnosia^37,38^, who typically fail to identify faces but can normally categorize faces from non-faces^39^. FFA responses are also preserved after surgical removal of OFA^40^, suggesting that the face processing network does not depend on a unique hierarchical flow of inputs. In healthy participants, TMS to OFA impairs face identity discrimination but not face categorization^41^. Furthermore, unlike MEG/EEG findings described above, fast fMRI sampling suggests that FFA activity might precede OFA activity when detecting faces (vs cars) that progressively appear in a noisy background^42^.

In sum, these two models disagree on the flow of face information within the core face network and suggest opposite roles for FFA and OFA in *face detection* and *identity recognition*. According to the hierarchical feedforward model, OFA mediates face detection by encoding local face elements, while FFA subserves identity recognition by integrating these elements into a complete face percept. Instead, in the non-hierarchical account, face detection is mediated by FFA through an extraction of global configural cues, while OFA is recruited in parallel for fine-grained feature analysis and allows subsequent identity recognition.

### Our study

We addressed this theoretical disagreement by designing a rt-fMRI NFB procedure where participants learned to induce a relative activity bias between FFA and OFA, a state we refer to as “ROI dominance”, on a trial-by-trial basis. Crucially, ROI self-regulation was immediately followed by a single behavioural probe that required both detection and recognition of a face (or animal). This enabled us to manipulate ROI activity while keeping task demands constant, and probe for any corresponding impact on behavioural performance. Our rationale builds on evidence of experience-dependent neuroplasticity^43^, indicating that NFB can enhance cognitive functions linked to the regulated region by recruiting neural populations overlapping with natural task performance and thus promote their engagement beyond NFB itself. Here, our participants received real-time NFB based on current ROI dominance, calculated as the moment-by-moment activity difference between the target (e.g., FFA) and the other (e.g., OFA) ROI. We hypothesized that self-regulating FFA or OFA dominance would influence their relative engagement and concomitant face processing performance, in line with previous work showing that spontaneous fluctuations of baseline activity in visual cortex can affect the quality of perceptual representations^44^ and bias behavioural responses to a subsequent stimulus^45^. Notably, better face perception performance was reported to correlate with higher *stimulus-evoked* activity in the core face network^45^ including FFA^9,28,46^, and with higher *pre-stimulus* activity in OFA^47^ or FFA^48,49^. However, these findings do not unveil the specific role of OFA vs. FFA.

Here, by manipulating *pre-stimulus* FFA or OFA dominance across trials through NFB, we could determine whether region-specific activity is instrumental to face detection and/or face recognition. According to the classic hierarchical model, OFA dominance would be expected to affect face detection, while FFA dominance should impact face recognition. Conversely, according to the non-hierarchical model, increased OFA dominance should mainly affect face recognition, while FFA dominance should influence detection (and possibly recognition as well). Please note we do not suggest that facial recognition or detection is resolved at a single cortical location like FFA or OFA, but instead surmise that these regions make distinct causal contributions to these processes, which should be uncovered by different functional relationships between NFB-induced activity and behavioural performance.

To characterize these effects, each participant underwent two distinct NFB training sessions, separately targeting FFA and OFA dominance. Given a well-established right hemispheric dominance in face perception^14,32^, we targeted only the right FFA and right OFA in all participants. Our results reveal for the first time that people can learn to self-regulate information flow across two co-activated regions in their brain, and that such neural biases can impact specific processing stages in visual perception (i.e., face detection vs recognition).

## Results

**Fig. 1:**
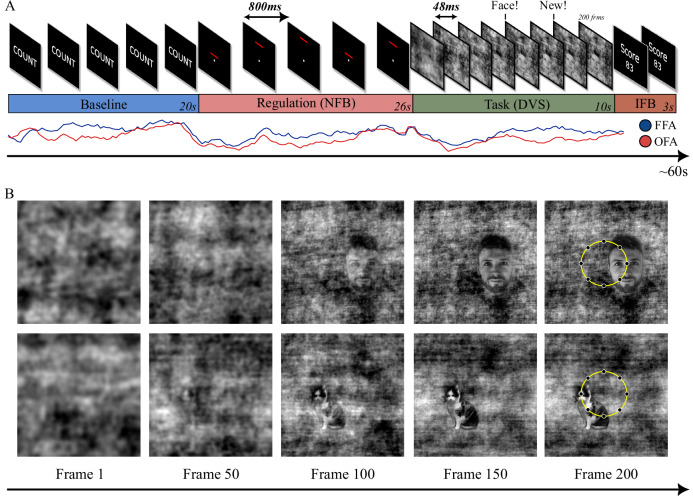
Single NFB trial. **A** Each trial unfolded in four phases. During the *baseline* phase (blue), participants counted down from 100. This was followed by the *regulation* phase (red), where participants received continuous NFB via a dynamic thermometer, representing the differential PSC (Eq. (2)) between the target ROI (e.g., FFA) and non-target ROI (e.g., OFA). In the immediately succeeding *visual task* phase (green), participants had to categorize the emerging image (FACE or ANIMAL: detection task) and determine its familiarity (OLD or NEW: recognition task). Each trial concluded with a brief *intermittent feedback* phase (IFB, dark red), providing a summary score of performance during the *regulation* phase. **B** Examples of a progressively denoised face (top row) and animal (bottom row) stimulus. Each frame was presented for 48 ms, for a total dynamic visual stimulation of 9.6 s. Yellow circles with white dots illustrate all possible spatial positions for stimulus appearance. NFB neurofeedback, FFA fusiform face area, OFA occipital face area, ROI region of interest, PSC percent signal change, IFB intermittent feedback, DVS dynamic visual stimulation. The face and animal images shown here are illustrative examples created and owned by the authors; the actual stimuli used in the experiment were sourced from the Umea University Database of Facial Expressions (UUDFE) and the Natural Face and Object Stimuli (NFOS) set, as described in “Methods”.

To investigate the role of FFA and OFA in face processing, we assessed how NFB-mediated modulation of activity in these regions influenced face detection and face recognition. Each participant completed two NFB sessions on consecutive days (see “Methods”), one targeting FFA dominance and the other OFA dominance via differential feedback signal, with each session comprising 7 runs of 7 trials (Fig. 1A). Per trial a face or animal stimulus was progressively revealed through dynamic visual stimulation (Fig. 1B). Through complementary stepwise analyses, we first verified the impact of real-time NFB on neural activity in target ROIs during regulation, comparing such effects between experimental (EXP; real NFB) and control (CONT; sham NFB) groups. Next, we evaluated whether enhanced FFA and OFA activity during regulation impacted their subsequent neural response during face detection and recognition, and whether this in turn affected behavioural performance. We employed time series (TS) analyses, linear mixed models (LMMs), and structural equation models (SEMs) to relate neural and behavioural data across these phases. We also examined debriefing questionnaires concerning individual regulation strategies. However, although participants reported various approaches (e.g., imagining familiar faces, recalling emotional expressions, focusing on facial features, etc.), no systematic differences emerged between strategies used to upregulate FFA versus OFA activity or between the EXP and CONT groups. This aligns with previous findings indicating that participants often lack direct phenomenal access to the precise mental processes underlying successful regulation^50,51^, as well as with the idea that NFB learning is also shaped by implicit processes such as reinforcement learning that may operate outside conscious awareness^2,52^.

### NFB self-regulation performance

#### Session by group TS comparisons

**Fig. 2:**
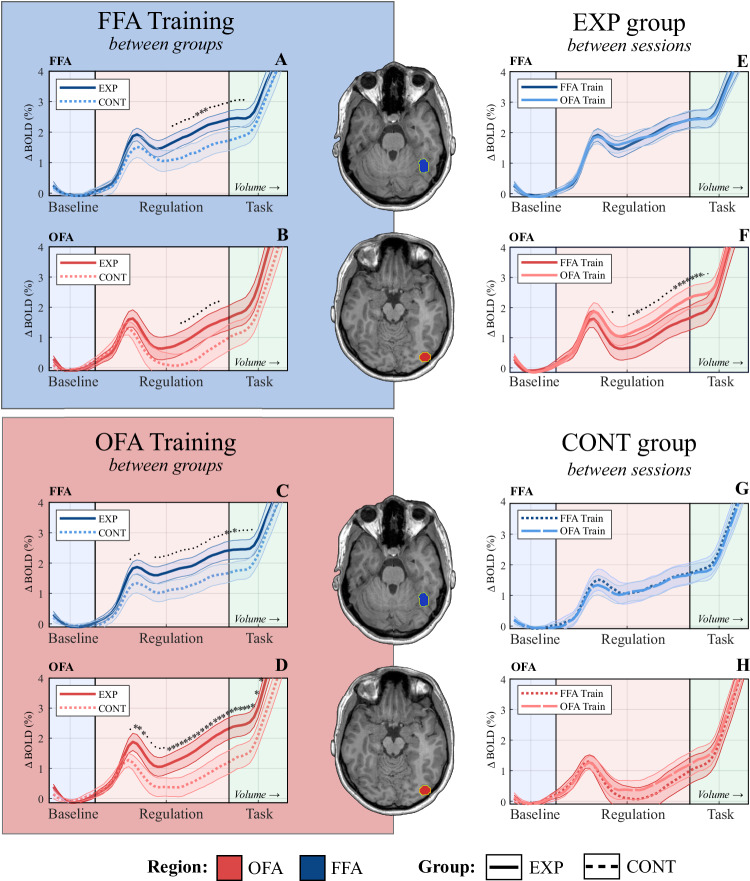
Neurofeedback regulation performance in target regions. The efficacy of neurofeedback-induced modulation is presented for each ROI and each training session, depicting mean changes in activity levels during baseline, regulation, and task phases (pooled across all regulation runs within a session), for the EXP and CONT groups. *Comparison between groups (left panel)*. **A**, **B** FFA Training: The EXP group shows (**A**) significantly higher self-regulation activity in FFA during the FFA training session, but (**B**) weaker and non-significant differences in the non-target OFA region, suggesting successful self-regulation. **C**, **D** OFA Training: The EXP group demonstrates (**C**) a weak and delayed increase in non-target FFA and (**D**) more pronounced and sustained modulation of OFA. *Comparison between sessions (right panel)*. **E**, **F** EXP group: Time-courses of activity show a selective neurofeedback modulation of (**E**) OFA during OFA training, but (**F**) no differential effect on FFA is observed in the EXP group in the same sessions, indicating region-specific regulation. **G**, **H** CONT group: No modulation of either ROIs is seen in this group. All data are pooled over all regulation runs from each session. Shaded error bands indicate ±1 SEM; time-courses are baseline-corrected for visualization. Volume-by-volume permutation results (uncorr.) are marked with asterisks for significant effects and dots for trending effects. Data are presented as mean values ± SEM (*n* = 22 independent participants in EXP group, *n* = 20 in CONT group). Statistical analysis: volume-by-volume permutation tests (10,000 permutations), uncorrected for multiple comparisons across time points. **A**–**D** independent-samples permutation test (label-shuffling), one-sided (EXP > CONT), significance at *p* > 0.95, trending at 0.90 <*p* ≤ 0.95. **E**, **F** paired permutation test (sign-flipping on within-subject difference scores; target session minus non-target session), two-sided, significance at *p* < 0.025 or *p* > 0.975, trending at 0.025 ≤ *p* < 0.05 or 0.95 <*p* ≤ 0.975. * *p* < 0.05, · trending. Source data are provided as a Source Data file. NFB neurofeedback, FFA fusiform face area, OFA occipital face area, ROI region of interest, EXP experimental group, CONT control group, SEM standard error of the mean, PSC percent signal change.

NFB efficacy was examined by comparing fMRI signal changes in FFA/OFA during self-regulation in each NFB session and each group (Fig. 2). Data were pooled across all successive runs of each training session (regulation blocks 1 to 7; see “Methods”). Overall, the EXP group exhibited more pronounced and sustained increases in FFA and OFA during regulation compared to the CONT group, particularly in sessions training the target region. The largest gains were observed in OFA during OFA training, with significant OFA activation from 8 s onwards (permutation test, *p* < 0.001, unc.), extending into the task phase (Fig. 2D). A weaker and delayed modulation was seen in FFA during this session. Similarly, FFA showed significant increases during FFA training (permutation test, *p* < 0.001; Fig. 2A), which were short-lived but more pronounced than those in the non-targeted OFA (Fig. 2B). Within-group comparisons for the EXP group revealed greater OFA enhancement in OFA versus FFA session (Fig. 2F), while FFA enhancements were similar in both sessions (Fig. 2E). However, a focused comparison between the initial and final runs of each training session revealed that FFA activity was significantly more pronounced in the FFA-targeted sessions than in the OFA-targeted ones during the final run, a contrast that was not observed in the initial run (Supplementary Fig. 1). The CONT group showed no session-specific differences whatsoever (Fig. 2G, H).

These results demonstrate that NFB training successfully modulated OFA, and to a lesser extent FFA, only in the EXP group. Thus, receiving reliable differential NFB information allowed participants to self-regulate activity in target ROIs. The seemingly weaker modulation of FFA (compared to OFA) across training sessions in the EXP group will be further discussed below. Furthermore, the fact that OFA activity during the FFA session was *higher* and not lower compared to the CONT group points to general upregulation effects (rather than suppression of non-targeted ROI).

#### Learning across runs

**Fig. 3:**
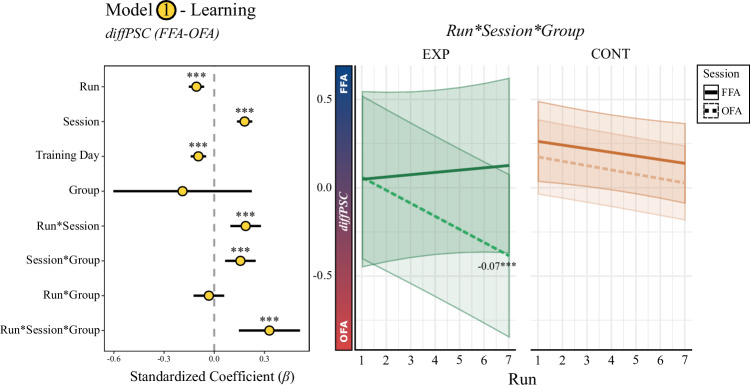
Learning effects in NFB regulation. Results of Linear Mixed Model 1 are shown for diffPSC across the two ROIs (FFA minus OFA), averaged over the regulation period from each successive run (1 to 7) in each training session. In the EXP group (left; green), FFA activity increased slightly and became progressively dominant during the FFA training session, whereas OFA activity increased robustly and became dominant during the OFA session. The CONT group (right; orange) showed a progressive reduction in FFA dominance in both sessions but no differential activity between the two ROIs over time. EXP group, *n* = 22; CONT group, *n* = 20; 3966 trial-level observations. Dot-whisker plots show coefficient estimates (centre) with 95% CIs (whiskers). Shaded bands represent ±95% CI of LMM-predicted marginal effects (centre: predicted mean). Statistics were obtained from a two-sided LMM (fixed effects: Run × Session × Group; random effects: participant and stimulus) fitted with REML and Satterthwaite degrees of freedom. Key effects: Run × Session × Group (*ß* = 0.33, 95% CI [0.15, 0.51], *p* < 0.001), Run × Session (*ß* = 0.19, 95% CI [0.10, 0.28], *p* < 0.001), Run (*ß* = −0.11, 95% CI [−0.15, −0.06], *p* < 0.001), Training Day (ß = −0.095, 95% CI [−0.14, −0.05], *p* < 0.001). The slope annotation (−0.07) reflects a post-hoc simple slope estimate for the OFA session in the EXP group and is FWER-corrected (Holm method) for 4 slope estimates. * *p* < 0.05, ** *p* < 0.01, *** *p* < 0.001. Source data are provided as a Source Data file. NFB neurofeedback, FFA fusiform face area, OFA occipital face area, EXP experimental group, CONT control group, LMM linear mixed model, diffPSC differential percent signal change.

In addition to the global session effects above, we examined NFB learning effects in each ROI across successive runs from each training session. We computed a LMM (with four factors: *Run*, *Session*, *Training Day*, and *Group*) using trial-wise data to evaluate differential PSCs between FFA and OFA over time (LMM 1, Fig. 3, left panel). This showed a significant main effect of *Run* (*ß* = −0.11, SE = 0.023, *t*(3917) = −4.58, 95% CI [−0.15, −0.06], *p* < 0.001) reflecting a gradual divergence of activity between ROIs across successive regulation periods, a main effect of *Session* (*ß* = 0.18, SE = 0.023, *t*(3917) = 7.78, 95% CI [0.14, 0.23], *p* < 0.001) indicating overall higher OFA dominance during OFA training relative to FFA training, and a *Session × Group* interaction (*ß* = 0.16, SE = 0.047, *t*(3917) = 3.36, 95% CI [0.07, 0.25], *p* < 0.001) confirming that this session-dependent pattern was driven by the EXP group. Additionally, there was an interaction of *Run* × *Session* (*ß* = 0.19, SE = 0.047, *t*(3917) = 4.04, 95% CI [0.10, 0.28], *p* < 0.001) indicating a progressively increasing OFA dominance in the OFA training session.

Critically, there was a significant *Run* × *Session* × *Group* interaction (*ß* = 0.33, SE = 0.093, *t*(3917) = 3.54, 95% CI [0.15, 0.51], *p* < 0.001) which revealed that this progressive difference between ROIs according to training session was driven solely by the EXP group (Fig. 3, right). Finally, we observed a negative main effect of *Training Day* on differential PSCs (not plotted; *ß* = −0.095, SE = 0.023, *t*(3917) = −4.07, 95% CI [−0.14, −0.05], *p* < 0.001), suggesting that FFA generally reduced its dominance during training.

Post-hoc analysis of the three-way interaction revealed a slight (albeit non-significant) increase in FFA dominance across runs during the FFA session (*ß* = 0.013, SE = 0.012, *t*(3917) = 1.13, 95% CI [−0.010, 0.036], *p.holm* = 0.260) and a robust increase in OFA dominance in the OFA session (*ß* = −0.074, SE = 0.011, *t*(3917) = −6.48, 95% CI [−0.096, −0.052], *p.holm* < 0.001). In contrast, the CONT group exhibited a progressive *reduction* in FFA dominance across runs in both sessions, without reaching any differential activity between ROIs (Fig. 3, right panel). For additional insights into the underlying data structure of LMM1, we refer the interested reader to Supplementary Fig. 2.

These results accord with the global session effects above and confirm a differential modulation of both FFA and OFA during NFB training in the EXP group, yielding a progressive dominance of the target ROI across successive regulation runs, unlike in the CONT group.

#### Whole brain analysis and overlap with target ROIs

**Fig. 4:**
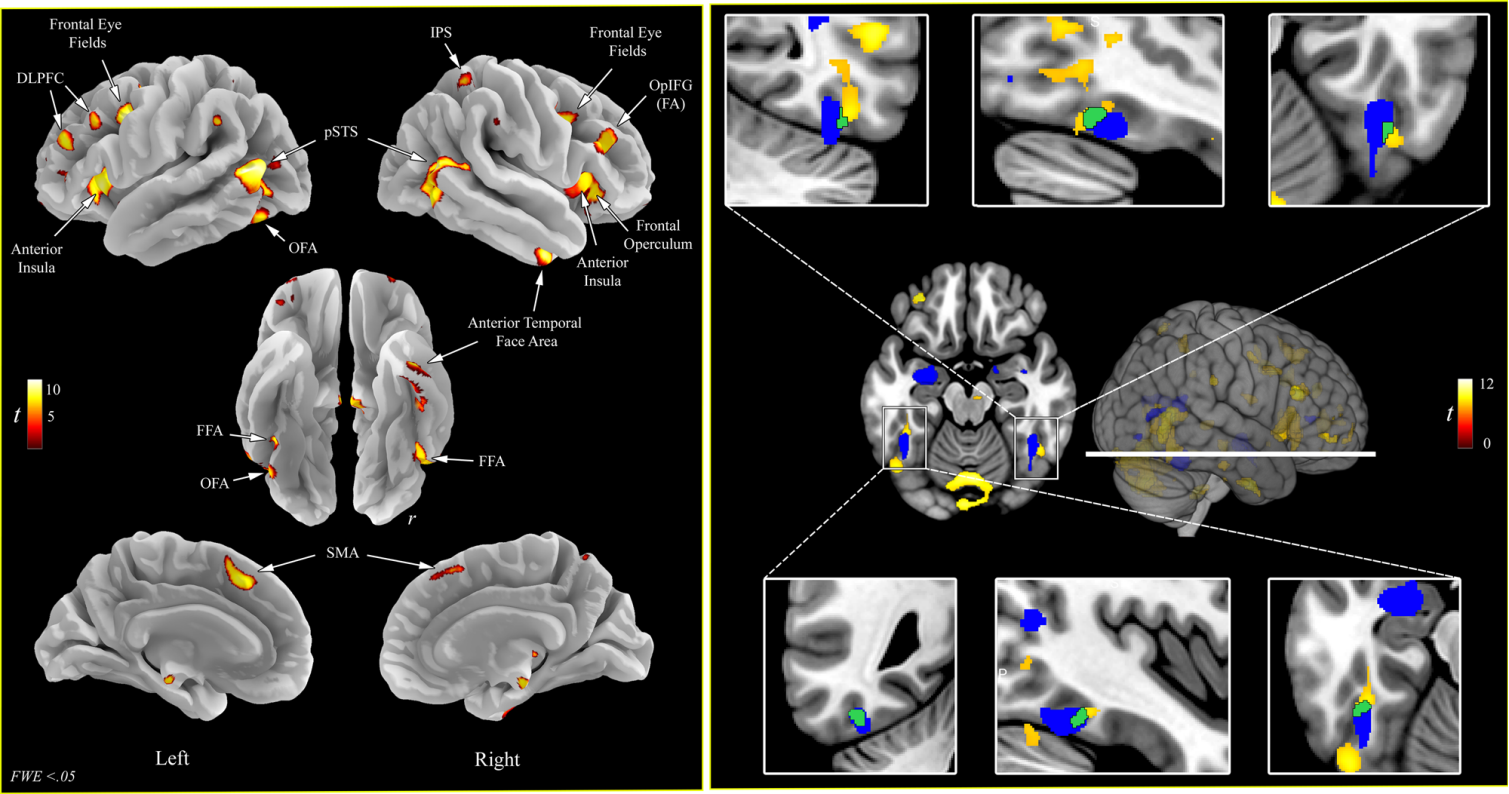
Effect of self-regulation on whole brain activity during NFB. Left panel: A distributed network of face-related regions was activated during NFB regulation periods (compared to baseline) in the EXP group. These regions dovetail neatly with the core and extended face network reported in the literature (see text for details). The CONT group showed no suprathreshold activation in the same contrast. Right panel: Overlay mapping between self-regulation increases during NFB training (yellow) and face-evoked activation during the functional localizer (blue) revealed highly focal overlap (green) in bilateral FFA and pSTS. Brain maps show results of second-level one-sample *t*-tests on first-level contrast images (regulation > baseline), corrected for multiple comparisons (voxel-level FWE < 0.05), rendered on an MNI152 template brain (*n* = 22 independent participants in EXP group, *n* = 20 in CONT group). Statistical maps are available upon request. NFB neurofeedback, FFA fusiform face area, OFA occipital face area, EXP experimental group, CONT control group, pSTS posterior superior temporal sulcus, IPS intraparietal sulcus, IFG inferior frontal gyrus, SMA supplementary motor area.

We also assessed brain activity during self-regulation using second-level contrasts (one-sample *t*-test) comparing NFB runs with baseline periods across the whole brain. This revealed activation of a widespread network for the EXP group (Fig. 4, left panel), unlike CONT, where no significant effects were found. These activations in the EXP group encompassed several nodes of the core and extended face network: bilateral FFA (right dominant), pSTS, anterior insula, left OFA, right IPS, and right anterior temporal cortex (overlapping with the Anterior Temporal Face Area)^53^. Additional activations were noted in right IFG, also frequently activated by faces^14^, as well as in bilateral frontal eye fields, left dorsolateral prefrontal cortex, and left (more than right) supplementary motor area.

To determine whether these activations overlapped with face-selective responses in visual cortex, we computed overlay maps comparing regulation-induced increases with face-localizer data. Figure 4 (right panel) depicts these results showing the face perception network (blue clusters, from face localizer) and self-regulation effects (yellow clusters, from NFB runs), as well as their overlaps (green). A selective overlap was evident in two bilateral regions in the EXP group, including the left and right *FFAs* and the left and right *pSTS*. No overlap was found in CONT group, indicating no reliable modulation of face-responsive areas.

### Impact of self-regulation on subsequent activation of target ROIs during face detection and recognition

**Fig. 5:**
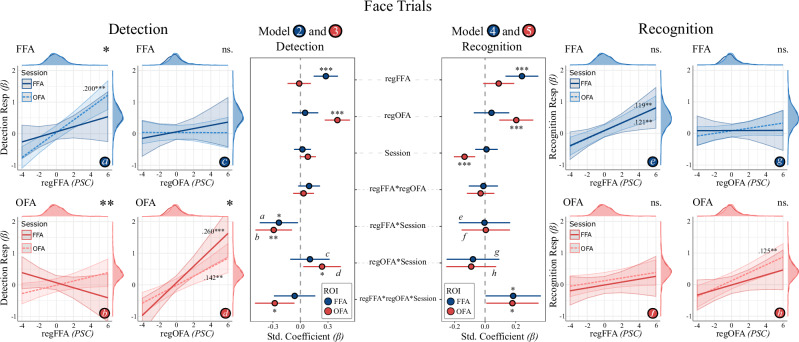
Self-regulation effects on subsequent task-evoked activity in each ROI during face detection and face recognition. Coefficient plots from LMMs demonstrate that self-regulation of FFA (blue) and OFA (red) selectively enhanced task-evoked responses within the same region (but not the other) during face detection (models 2 and 3) and recognition (models 4 and 5). **a**–**d** illustrate significant effects in each model for detection and recognition, highlighted in dot-whisker plots by small letter insets. Slopes show LMM predictions with 95% CIs (shaded; centre: predicted mean). Dot-whisker plots show coefficient estimates (centre) with 95% CIs (whiskers). Marginal plots show density distributions by session. EXP group only, *n* = 22; 1494 trial-level observations (independent participants). Statistics were obtained from two-sided LMMs (fixed effects: regFFA × regOFA × Session, with Run, Training Day, and detection frames as covariates; random effects: participant and stimulus) fitted with REML and Satterthwaite degrees of freedom. Estimates and significance annotations on individual slopes reflect post-hoc simple slope tests, FWER-corrected using the Holm method. **Detection (left)**. Higher FFA regulation activity led to (**a**) higher FFA detection responses (regFFA: *ß* = 0.28, 95% CI [0.15, 0.41], *p* < 0.001), particularly during OFA training sessions (regFFA × Session: *ß* = −0.23, 95% CI [−0.44, −0.03], *p* = 0.027), and (**b**) higher OFA detection responses during OFA training sessions but lower OFA detection responses during FFA training sessions (regFFA × Session: *ß* = −0.29, 95% CI [−0.49, −0.09], *p* = 0.004). In contrast, OFA regulation activity produced (**c**) no effect on FFA detection responses, but (**d**) significantly enhanced OFA detection responses (regOFA: *ß* = 0.40, 95% CI [0.27, 0.54], *p* < 0.001), particularly during FFA training sessions (regOFA × Session: *ß* = 0.24, 95% CI [0.03, 0.44], *p* = 0.024). **Recognition (right)**. Higher FFA regulation activity led to (**e**) significantly enhanced FFA recognition responses (regFFA: *ß* = 0.24, 95% CI [0.13, 0.35], *p* < 0.001), but (**f**) no effect on OFA recognition responses. Conversely, OFA regulation produced (**g**) no significant modulation of FFA recognition responses, but (**h**) marked increases in OFA recognition responses (regOFA: *ß* = 0.20, 95% CI [0.09, 0.32], *p* < 0.001). * *p* < 0.05, ** *p* < 0.01, *** *p* < 0.001, ns. not significant. Source data are provided as a Source Data file. FFA fusiform face area, OFA occipital face area, ROI region of interest, LMM linear mixed model, CI confidence interval.

We then analysed how self-regulating FFA and OFA influenced their subsequent recruitment during the face detection and face recognition task. Four LMMs were computed, as depicted in Fig. 5. LMMs 2 and 3 assessed the effects of self-regulation on respectively FFA and OFA during face detection, while LMMs 4 and 5 evaluated these effects during face recognition. Given the lack of NFB modulation in CONT participants, this analysis was restricted to the EXP group and exclusively considered trials with face stimuli.

#### Detection-related activity

Heightened self-regulation of FFA and OFA during NFB runs led to stronger face detection responses in the same (but not the other) region (Fig. 5, left panels). Specifically, increased FFA self-regulation was linked to greater face detection responses in FFA (Fig. 5, model 2*, regFFA*: *ß* = 0.27, SE = 0.067, *t*(1453) = 4.11, 95% CI [0.14, 0.41], *p* < 0.001) but not OFA (model 3), and vice versa for OFA self-regulation (Fig. 5, model 3, *regOFA*: *ß* = 0.40, SE = 0.070, *t*(1413) = 5.76, 95% CI [0.27, 0.54], *p* < 0.001). Additionally, the effect of NFB self-regulation on detection responses varied by training session. That is, a positive influence of FFA self-regulation on its own detection responses was observed during both training sessions, but more prominent during the OFA than FFA session (Fig. 5, model 2, panel a, *regFFA* × *Session*: *ß* = −0.23, SE = 0.11, *t*(1468) = −2.21, 95% CI [−0.44, −0.03], *p* = 0.027). Similarly, OFA self-regulation positively influenced OFA detection responses in both sessions, but more during the FFA session (model 3, panel d, *regOFA* × *Session*: *ß* = 0.24, SE = 0.10, *t*(1463) = 2.25, 95% CI [0.03, 0.44], *p* = 0.024).

We also observed a session-dependent interaction for OFA detection responses, driven by the preceding FFA self-regulation activity (Fig. 5, model 3, panel b, *regFFA* × *Session*: *ß* = −0.29, SE = 0.10, *t*(1466) = −2.87, 95% CI [−0.49, −0.09], *p* = 0.004). Post-hoc simple slopes showed that during FFA training, greater FFA upregulation tended to suppress OFA detection responses (*ß* = −0.080, SE = 0.041, *t*(1471) = −1.92, 95% CI [−0.161, 0.002], *p.holm* = 0.109), while during OFA training the trend was reversed (*ß* = 0.066, SE = 0.041, *t*(1477) = 1.61, 95% CI [−0.014, 0.146], *p.holm* = 0.109), consistent with a dynamic rebalancing within the face processing network whereby prioritizing FFA leads to a relative downregulation of OFA during detection.

Furthermore, there was a triple interaction for OFA detection responses, driven by a modulation of regulation effects on OFA by concurrent FFA regulation, with a relative suppression of OFA responses during detection when FFA activity was higher in the FFA training session.

#### Recognition-related activity

Like detection responses, higher FFA regulation activity was related to significantly *enhanced* recognition responses in FFA (Fig. 5, right-side panels, Model 4, *regFFA*: *ß* = 0.24, SE = 0.055, *t*(1468) = 4.40, 95% CI [0.13, 0.35], *p* < 0.001) but not in OFA (Model 5). Conversely, higher OFA regulation activity was related to enhanced recognition responses in OFA (model 5, *regOFA*: *ß* = 0.20, SE = 0.057, *t*(1482) = 3.61, 95% CI [0.09, 0.32], *p* < 0.001) but not in FFA (model 4). In addition, OFA recognition activity was significantly higher during OFA training (model 5, *Session*: *ß* = −0.14, SE = 0.036, *t*(1462) = −3.80, 95% CI [−0.20, −0.07], *p* < 0.001). The interaction effects between *region* and *session* were not significant.

Taken together, these data indicate that the magnitude of FFA or OFA modulation during NFB self-regulation significantly influenced their subsequent recruitment during the face processing task, with increases in FFA and OFA during NFB leading to stronger face *detection* and *recognition* responses in the same region. However, interaction effects also suggest that FFA regulation also influenced subsequent OFA activity depending on training conditions, with reduced OFA recruitment during face detection, pointing to reciprocal interactions between areas within the face processing network.

### Behavioural performance in the visual task

#### Response latencies

To compare face detection and recognition performance between conditions, we computed two mixed-effects ANOVAs on response latencies (image frames corresponding to correct detection and correct recognition) across trials, with the factors Group (EXP, CONT), Training session (FFA, OFA), and Stimulus (Faces, Animals). Outliers were identified using R’s rstatix “identify_outliers” function, which flags values outside Q3 + 1.5 × IQR or Q1 − 1.5 × IQR. This led to excluding two EXP participants, leaving 20 in each group for the ANOVAs. Visualizations of the effects described below are shown in the supplementary materials (Supplementary Fig. 5).

For *detection* latencies, we found significant main effects of *Group* (*F*(1,38) = 4.21, *p* = 0.04) and *Stimulus* (*F*(1,38) = 43, *p* < 0.001), but no main effect of *Training Session* (*F*(1,38) = 0.09, *p* = 0.76) nor interactions. Post-hoc pairwise comparisons (Holm-corrected across four tests) indicated that the EXP group detected stimuli faster (*M* = 90.4, *SD* = 15.7) than the CONT group (*M* = 96.5, *SD* = 13.6): *t*(158) = −2.64, *p.holm* = 0.009, while faces (*M* = 85.7, *SD* = 11) were detected faster than animals (*M* = 101, *SD* = 14.4): *t*(79) = 8.89, *p.holm* < 0.001) in both groups.

For *recognition*, we again found significant main effects of *Group* (*F*(1,38) = 4.85, *p* = 0.03) and *Stimulus* (*F*(1,38) = 19.83, *p* < 0.001), but no effect of *Training Session* (*F*(1,38) = 0.45, *p* = 0.56) nor interactions. Post-hoc pairwise comparisons indicated that the EXP group recognized stimuli faster (*M* = 133, SD = 15.1) than the CONT group (*M* = 140, *SD* = 10.7): *t*(158) = −3.61, *p.holm* < 0.001, while faces (*M* = 133, *SD* = 13.4) were recognized faster than animals (*M* = 144, *SD* = 12.6): *t*(79) = 5.89, *p.holm* < 0.001.

### The effects of ROI detection and recognition responses on behavioural performance

**Fig. 6:**
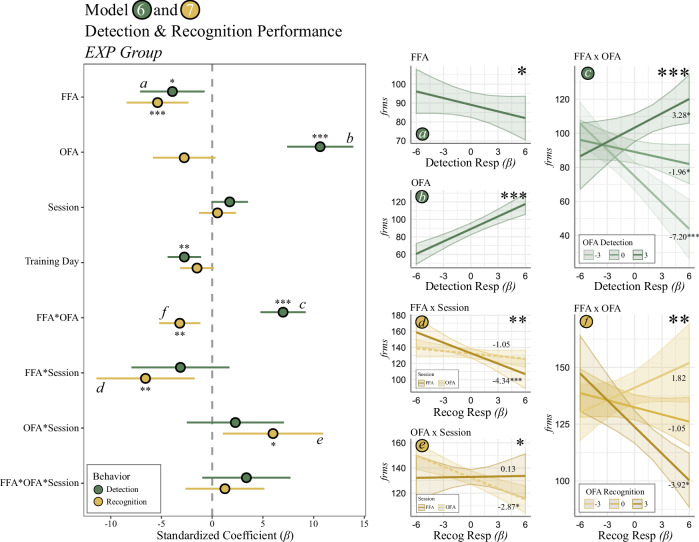
Relation of behavioural performance with task-evoked activity in FFA and OFA during face detection and face recognition. Performance is expressed in terms of correct response latencies (image frames). Two LMMs were computed to assess the impact of each ROI activation at detection (model 6; green) and recognition (model 7; yellow) times on behavioural detection and recognition latencies, respectively. EXP group only, *n* = 22; 1494 trial-level observations (independent participants). Dot-whisker plots show standardized coefficient estimates (centre) with 95% CIs (whiskers). Slope panels show LMM-predicted marginal effects (centre: predicted mean) with 95% CIs (shaded bands). Small letter indices in LMM coefficient plots refer to the linear regression panels illustrating the corresponding effect, split according to relevant conditions. Statistics were obtained from two-sided LMMs (fixed effects: FFA × OFA × Session, with Training Day as covariate; Model 7 additionally includes detection latency; random effects: participant and stimulus) fitted with REML and Satterthwaite degrees of freedom. Estimates and significance annotations on individual slopes reflect post-hoc simple slope tests, FWER-corrected using the Holm method. **Detection (green)**. Better (faster) face detection performance is associated with (**a**) increased FFA (*ß* = −3.92, 95% CI [−7.10, −0.74], *p* = 0.016) and (**b**) attenuated OFA recruitment (*ß* = 10.64, 95% CI [7.39, 13.90], *p* < 0.001), as further reflected by (**c**) a significant interaction between FFA and OFA activity (*ß* = 6.99, 95% CI [4.76, 9.22], *p* < 0.001). These data suggest that optimal face detection performance is achieved with concomitantly high FFA and low OFA activity. **Recognition (yellow)**. Better (faster) face recognition performance is related to (**d**) higher FFA activation (*ß* = −5.38, 95% CI [−8.43, −2.33], *p* < 0.001), especially during FFA training (FFA × Session: *ß* = −6.57, 95% CI [−11.40, −1.74], *p* = 0.008), and (**e**) higher OFA activation, primarily during OFA training (OFA × Session: *ß* = 5.99, 95%  CI [1.06, 10.93], *p* = 0.017). In addition, (**f**) a notable FFA × OFA interaction indicates that the positive impact of each ROI on recognition performance is amplified by co-activation of the other ROI (*ß* = −3.19, 95% CI [−5.22, −1.16], *p* = 0.002), unlike the negative interaction for detection. Detection latency was included as a covariate (*ß* = 21.44, 95% CI [19.19, 23.69], *p* < 0.001). Source data are provided as a Source Data file. FFA fusiform face area, OFA occipital face area, LMM linear mixed model, EXP experimental group, CI confidence interval, ROI region of interest.

We next examined the relationship between task-evoked activity in FFA/OFA during face detection or recognition, and corresponding behavioural performance in the EXP group. Performance was measured via detection and recognition latencies, defined as the image frame at which correct responses were made. Two LMMs (Fig. 6; models 6 and 7) were computed for each response, incorporating *target region*, *training session*, their interaction, and *training day* as factors. See supplementary materials (Supplementary Fig. 6) for numerical statistics.

For *detection* (Model 6), higher FFA activity was associated with quicker face detection (i.e., lower image frame numbers, *ß* = −3.92, SE = 1.62, *t*(1440) = −2.42, 95% CI [−7.10, −0.74], *p* = 0.016, Fig. 6a), while increased OFA activity led to slower detection (*ß* = 10.64, SE = 1.66, *t*(1437) = 6.41, 95% CI [7.39, 13.90], *p* < 0.001, Fig. 6b). A significant interaction between FFA and OFA activities (*ß* = 6.99, SE = 1.14, *t*(1422) = 6.14, 95% CI [4.76, 9.22], *p* < 0.001) indicated optimal detection performance with high FFA and concurrent low OFA levels (Fig. 6c, with FFA effects on behaviour shown for 3 levels of OFA activity).

For *recognition* (Model 7), better performance was also associated with high FFA activity (*ß* = −5.38, SE = 1.55, *t*(1484) = −3.46, 95% CI [−8.43, −2.33], *p* < 0.001), particularly in the FFA-training session (*FFA* × *Session;* *ß* = −6.57, SE = 2.47, *t*(1464) = −2.67, 95% CI [−11.40, −1.74], *p* = 0.008, Fig. 6d). The influence of OFA activity was session-dependent, enhancing recognition only during OFA-training (*OFA* × *Session*: *ß* = 5.99, SE = 2.52, *t*(1466) = 2.38, 95% CI [1.06, 10.93], *p* = 0.017, Fig. 6e). There was also a significant interaction between FFA and OFA, again revealing that concurrent high activity in both ROIs facilitated recognition (*FFA* × *OFA*: *ß* = −3.19, SE = 1.03, *t*(1467) = −3.09, 95% CI [−5.22, −1.16], *p* = 0.002). This interaction was visually most pronounced during OFA training (Fig. 6f), though the session difference was not statistically significant (FFA × OFA × Session: *ß* = 1.26, SE = 1.98, *t*(1465) = 0.63, 95% CI [−2.63, 5.15], *p* = 0.526)

These results demonstrate distinct functional relationships between ROI activity and face processing performance. Higher FFA activity aided both detection and recognition, while OFA activity effects varied: detrimental for detection but beneficial for recognition. These effects were more pronounced in the respective training sessions.

### Path analyses with structural equation modelling

**Fig. 7:**
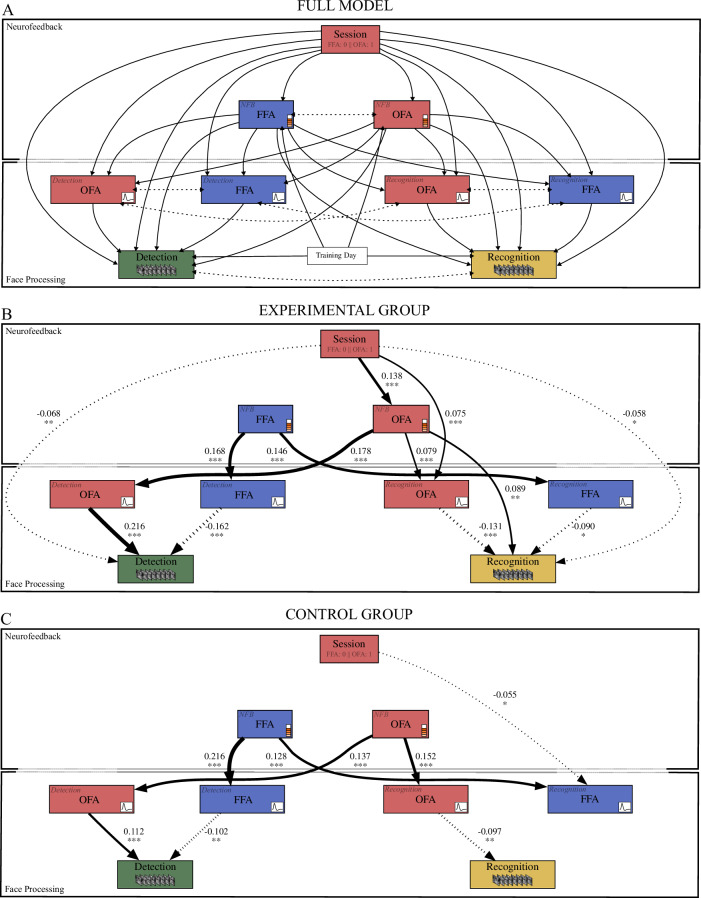
Path analysis of brain-behaviour relationships during neurofeedback modulation and task performance (using SEM), demonstrating the sequence of modulations initiated by NFB training. The analysis assesses how NFB training (top) influences ROI self-regulation and, subsequently, the functional and behavioural aspects of face processing (bottom). **A** Displays the full model tested, including all proposed connections (solid lines) and covariances between variables (dashed lines). In (**B** and **C**), only significant pathways are illustrated, with positive and negative effects represented by solid and dashed lines, respectively. **B** Depicts significant model estimates for the experimental EXP group, illustrating the widespread effects of NFB training on ROI regulation activity and subsequent face processing. **C** Presents the results of the same path analysis on the CONT group, revealing a distinct absence of NFB effects on ROI self-regulation, while a similar, yet significantly attenuated, pattern of effects between ROI activity and face processing behaviour remains. See main text for more details. Data are presented for *n* = 22 independent participants in the EXP group and *n* = 20 in the CONT group (trial-level observations). Statistical analysis: path analysis (structural equation modelling) fitted via maximum likelihood estimation (semopy^85^), two-sided. Path coefficients and *p*-values are estimated simultaneously within a single model; * *p* < 0.05, ** *p* < 0.01, *** *p* < 0.001. Source data are provided as a Source Data file. NFB neurofeedback, FFA fusiform face area, OFA occipital face area, ROI region of interest, SEM structural equation modelling, EXP experimental group, CONT control group.

Finally, we conducted a path analysis to obtain a comprehensive view of brain-behaviour relationships linking FFA/OFA activity to face processing during both NFB modulation and task performance. This analysis was computed using all face trials from both the EXP (Fig. 7B) and CONT (Fig. 7C) groups. Path coefficients were standardized for direct comparisons, and only significant effects are presented in Fig. 7 (see Supplementary Fig. 7 for complete results). In these models, solid lines represent positive relationships, while dashed lines indicate negative associations. Because “Session” was set to 1 for the OFA session, a positive coefficient for this factor implies a difference favouring the OFA regulation session relative to the FFA session. As behavioural performance is expressed in image frame counts, negative values for detection and recognition outcomes imply faster response times and thus better performance.

#### Model fit evaluation

Both the EXP and CONT models showed satisfactory fit to the data, with RMSEA values of 0.017 and 0.02, and chi-square values of 12.8 (*p* = 0.16) and 13.83 (*p* = 0.12), respectively.

#### Face detection effects (Fig. 7B, bottom green box)

In the EXP group, OFA training led to higher OFA activity (*β* = 0.138, *z* = 5.405, *p* < 0.001) and subsequently higher detection-related activity (*β* = 0.178, *z* = 5.10, *p* < 0.001), which in turn was related to longer detection latencies (*β* = 0.216, *z* = 5.51, *p* < 0.001). However, a direct negative effect of session on detection latencies (*β* = −0.068, *z* = −2.59, *p* = 0.010) suggested an overall, albeit smaller, benefit in the OFA training session, independent of task-evoked activity. Conversely, while FFA training did not significantly alter FFA regulation activity, FFA regulation activity predicted higher detection-related responses in FFA (*β* = 0.168, *z* = 4.84, *p* < 0.001), which in turn predicted shorter detection latencies and therefore better behavioural performance (*β* = −0.162, *z* = −4.16, *p* < 0.001).

#### Face recognition effects (Fig. 7B, yellow box)

OFA training enhanced both regulation and recognition-related activity in OFA (*β* = 0.138, *z* = 5.41, *p* < 0.001 and *β* = 0.079, *z* = 2.93, *p* = 0.003 respectively), with the latter inversely linked to recognition latencies (*β* = −0.131, *z* = −3.45, *p* < 0.001), signifying faster and improved performance. Additionally, OFA training directly influenced face recognition performance (*β* = −0.058, *z* = −2.23, *p* = 0.026), independent of ROI activity effects. Although FFA training did not significantly increase FFA regulation activity, FFA regulation activity still enhanced recognition-related activity (*β* = 0.146, *z* = 5.09, *p* < 0.001), which in turn increased recognition performance (*β* = −0.090, *z* = −2.40, *p* = 0.016), similar to the OFA effects.

Overall, these SEM results further demonstrate that upregulation of FFA and OFA by NFB produced distinct effects: FFA enhancement improved both face detection and recognition, while OFA upregulation hindered detection but aided recognition.

In contrast, the CONT group exhibited only weak “local” connections between task-evoked activity in each ROI and behavioural detection/recognition performance (Fig. 7C). However, no significant mediation paths between NFB sessions and either ROI activity or task performance were observed. These findings suggest the lower paths might reflect the intrinsic functional architecture of the core face processing network, regardless of the superimposed modulations of target ROIs by self-regulation during NFB.

## Discussion

Our study leveraged real-time fMRI to achieve self-regulation of two different, but functionally interconnected brain areas, and thus probe for their relative contribution to face perception. Doing so, we were able to modulate region-specific, pre-stimulus activity to address opposing theoretical hypotheses, i.e., hierarchical versus non-hierarchical models of the core face processing network. Across two sessions, participants trained to upregulate their FFA (relative to OFA) or OFA (relative to FFA) prior to a visual task requiring both face detection and face recognition. Results show that NFB successfully modulated our target ROIs in a differential manner. Furthermore, this modulation was driven by face-selective effects, as evidenced by concomitant upregulation of other nodes of face-responsive networks. These effects were not observed in a control group receiving sham NFB. Of note, self-regulation induced progressive increases in the target ROI reflecting differential learning for both areas, but these effects were larger in OFA than FFA (with more sustained activity in the latter regardless of training condition; see below). Critically, we observed a region-specific impact on face processing. FFA modulation had a selective influence on behavioral face detection performance, while face recognition was influenced by both FFA and OFA activity.

These data provide evidence for a non-hierarchical model of face processing, whereby face detection may arise in FFA prior to OFA recruitment for recognition^32^, unlike a traditional hierarchical processing flow from occipital to temporal visual cortex^13,15,20^.

### General behavioural performance

We found no global difference in task performance across sessions for either group, suggesting no broad behavioural gains from NFB training, despite its impact on ROI activity. Faces were detected and recognized faster than animals by both groups, and the EXP group performed generally faster than the CONT group. It remains unclear whether this speed difference was due to NFB training. It is possible that our behavioural measures were insensitive to more subtle changes, although our trial-by-trial analysis revealed that behavioural performance was reliably influenced by stimulus-evoked activity during the task, which was itself related to NFB upregulation in the EXP group (see Fig. 7).

### Self-regulation of pre-stimulus brain activity

Differential regulation of target ROIs was seen in the EXP group, unlike CONT, even though all participants were exposed to the same training protocol and blind to their condition. The EXP group successfully increased their OFA activity during OFA training (compared to FFA training or CONT group) and conversely showed higher FFA activity during FFA training relative to the CONT group, although average FFA increases were similar across both OFA and FFA sessions. Moreover, only the EXP exhibited a significant progression of activity in the target ROI within each training session, indicative of successful learning. This was again particularly apparent for OFA, while there was only a mild (nonsignificant) increase in FFA dominance during FFA NFB. This pattern suggests a general FFA dominance, persistent across conditions and groups, but progressively reduced during OFA regulation in the EXP group (see Fig. 3). This might reflect ceiling effects in FFA activity or more constant (e.g., mandatory) engagement of this region in current task context (e.g., due to face processing demands, see refs. ^49,54,55^), which was however partly counteracted by OFA NFB training. This pervasive FFA bias mitigating self-regulation may accord with a “natural” FFA dominance within the face network, consistent with the hypothesis of an obligatory “entry node” for face processing^33^. While these results support a differential modulation of FFA and OFA activity by NFB, we acknowledge that evidence for robust regulation effects in FFA would have been more convincing if a clear main effect, akin to that observed for OFA, had emerged.

While FFA and OFA were delineated based on their response to faces using a standard functional localizer, these regions are not *exclusively* engaged in face processing^56–59^. Because self-regulation was achieved through purely endogenous top-down efforts, their modulation could have been associated with a wider visual network (partly) unrelated to face processing. Offline analysis of whole-brain activity during NFB confirmed however, that, in the EXP group, FFA (bilateral) and OFA (left) were selectively co-activated with regions comprised in the extended face network (including pSTS, IFG, and presumably Anterior Temporal Face Area (AT-FA), indicating that self-regulation engaged a coherent face-responsive brain system (Fig. 4). This recruitment did not differ between OFA and FFA training, suggesting that globally similar face processing regulation strategies were utilized but with region-specific modulations according to NFB condition (ROI analyses). None of these effects were seen in the CONT group, further demonstrating that reliable feedback from FFA/OFA resulted in a more consistent engagement of face-related regulation strategies. These findings support the idea that NFB allowed participants to activate (and potentially “prime”) neural processes involved in face perception in each target ROI, rather than unspecific visual processes.

### Effects of pre-stimulus self-regulation activity on task-evoked neural responses

Upregulation of FFA prior to the visual task led to stronger recruitment of FFA (but not OFA) at the time of face detection and recognition, and vice versa for upregulation of OFA. These findings accord with our hypothesis that changes in pre-stimulus activity through self-regulation could lead to ROI-specific biases during face processing. These effects were substantiated by our analysis considering detection- and recognition-related responses for each ROI separately (LMM), as well as our path analysis (SEM) that included both direct and indirect influences on successive phases of a given trial. The latter additionally highlighted that, for OFA, the influence of training this ROI on its subsequent response during recognition was not solely mediated by pre-stimulus activity (unlike OFA detection responses) but also by other effects due to the training session itself. Thus, while OFA training induced higher OFA pre-stimulus activity that in turn increased OFA recognition responses, other factor(s) unique to OFA training could have affected its activity during face recognition. This might reflect that OFA training primed OFA processes contributing to recognition or modulated other areas that in turn affected OFA recognition activity. We cannot fully exclude such contribution of regions outside the target ROIs, but additional control analyses on the right pSTS (another co-activated face-responsive node) showed no differential effects in this region linked to NFB training or behavioural performance (see Supplementary Figs. 8 and 9).

Interestingly, we found that the relationship between FFA regulation activity and FFA detection responses was stronger during OFA training, while the relationship between OFA regulation activity and OFA detection responses was stronger during FFA training. This effect would accord with a form of adaptation where successful regulation of the target ROI (e.g., FFA in FFA training) could prime face processing in this region (e.g., by pre-activating some neural representation) which may subsequently lead to a reduced response during face detection (akin to repetition suppression: ref. ^27^). However, as a similar pattern was observed for animal trials (see Supplementary Fig. 4), any such priming was likely to act on more general visual processes. At the same time, greater upregulation of FFA during FFA (but not OFA) training was associated with a relative suppression of OFA detection responses. This suggests that by prioritizing FFA detection activity through prior upregulation, a relative downregulation of OFA may occur as part of a functional rebalancing of processing strategy induced by NFB. Together, these findings suggest that NFB-induced FFA modulation may enhance its sensitivity both by priming its representations and by downregulating competing pathways in OFA, thus optimizing its proposed functional role: face detection (see section “Neurofeedback training”).

As the CONT group showed no significant NFB regulation on target ROIs, we did not report similar detailed analysis on all task conditions, but closer inspection of their data accord with our conclusions about NFB-specific effects in EXP group. Neither ROI time-series nor SEM results showed any evidence of differential training-related modulation in CONT group. This again highlights the specificity of NFB training in driving neural changes in the EXP group.

In sum, our findings reveal that modulating FFA and OFA through NFB alters their response to subsequent visual stimuli in a region- and task-dependent manner, unveiling their respective contribution to face perception. We speculate that these changes may facilitate or “prime” neuronal processes engaged during stimulus processing, perhaps via sustained changes in synaptic weights or short-term plasticity within the target ROI, which could reverberate beyond the immediate regulation phase. This functional priming of FFA or OFA circuits could enhance the readiness or efficiency of neural computations necessary for face detection and recognition, presumably underlying concomitant changes in behavioural performance.

### Effects of FFA/OFA modulations on face detection performance

Most critically, our design enabled us to unveil distinct patterns of functional relationships between target ROIs and behavioural performance at two different perceptual stages of face processing, i.e., detection vs. recognition. The exact neural substrates of these two processes has remained debated^15,32^. Here, we show that face detection was faster with higher FFA but lower OFA recruitment. Moreover, the benefit of FFA increases on detection was contingent on concomitant OFA activity and amplified when the latter was attenuated, with a statistically significant interaction between activity in the two regions. This suggests a form of interference or competition produced by OFA during the initial stage of face processing. Importantly, the optimal functional profile: high FFA activity with reduced OFA interference) was precisely the state induced through NFB (section “Pre-training”), suggesting that our NFB training actively rebalanced the network toward a behaviourally efficient configuration. Taken more broadly, these data accord with a non-hierarchical account of face processing where FFA has a primary role in face detection, possibly acting as a domain-specific categorization mechanism and an entry point into the face processing network^33^.

Remarkably, the benefits of higher FFA and lower OFA activity on face detection in the EXP group was also seen in the CONT group, but much weaker and unrelated to NFB training. Furthermore, this was not seen for animal detection in either group. These data dovetail with an intrinsic functional architecture of face processing where FFA (but not OFA) plays a pivotal role in face detection. Thus, while evoked ROI activity influenced concurrent face detection responses in both groups, these effects were modulated by prior self-regulation and had a stronger impact on face detection performance in the EXP group.

The fact that high OFA activity interferes with face detection processes mediated by FFA might be explained in several ways. Firstly, if OFA is preferentially involved in the local, fine-grained analysis of internal face features^13,15,32^, stronger reliance on these processes could be detrimental to initial stimulus categorization based on configural cues. For example, focusing on local details from an eyebrow might be inefficient to rapidly distinguish a face from an animal. In contrast, FFA might preferentially encode a crude, more global representation of face structure^32^, crucial for detecting the “faceness” of visual stimuli. Moreover, unlike FFA, the OFA does not encode the position of internal parts^60^, such that extracting local details in earlier processing stages could potentially disrupt the computation of a global face configuration in FFA, leading to slower face detection times. OFA may also partly overlap with the lateral occipital complex that encodes surface and texture information across various object categories^61^, possibly contributing to finer face analysis and identification but not detection.

### Effects of FFA/OFA modulations on face recognition performance

A key finding is that, unlike face detection, optimal face recognition was achieved with combined recruitment of both FFA and OFA. While task-evoked increases in either ROI predicted faster recognition, concurrent increase in *both* produced further benefits in behavioral performance. This synergetic interaction suggests that the positive contribution of one region to face recognition is enhanced by co-activation of the other region. Accordingly, finer face details represented in OFA (e.g., local feature shape or surface information) might be combined with a concomitant representation of face structure in FFA (e.g., global configuration and metrics) to enable successful recognition. Alternatively, FFA may itself mediate an integration process whereby local details received from OFA are incorporated into the FFA representation of an individual face.

Again, this joint effect of FFA and OFA activity on recognition was seen in both the EXP and CONT groups, consistent with an intrinsic functional architecture of the face network. However, the effects of each ROI in isolation (LMM 6 & 7) were significant only in the EXP group, implying that enhanced recruitment of one region through NFB could effectively boost or “prime” their own functional computations (e.g., local feature extraction or global whole-face integration in OFA or FFA, respectively). Accordingly, a significant effect of training session was observed exclusively in the EXP group. Here, the positive impact of higher FFA recruitment on recognition performance was stronger in the FFA-training session (and weaker in the OFA-training session). Conversely, higher OFA recruitment positively affected recognition only after OFA-training (not significantly after FFA-training). As such, face related computations mediated by each region could be “primed” to a greater degree during the corresponding NFB training and lead to faster face recognition in both cases. No such training effect was seen after sham NFB in the CONT group.

In sum, we show both FFA and OFA play a key role in face recognition, but their interaction is crucial for optimal performance. Importantly, these data shed new light on neural mechanisms of face recognition deficits after brain damage, such as prosopagnosia^62^. Despite abundant neuropsychological research, much debate still surrounds the exact substrates of prosopagnosia^63,64^. Our results provide new explanations for why lesions affecting both areas simultaneously can cause severe and persistent deficits in face recognition^65,66^, while reconciling these observations with lesion studies suggesting that damage to OFA might be sufficient to cause selective prosopagnosia despite sparing of FFA^67^.

### Limitations

Our innovative methodology leverages rt-fMRI to probe distinct cortical nodes within the same functional network, yet bears some limitations. Firstly, we did not establish clear face-specificity concerning target ROI activity or behavioural performance. Although we used animals as control images, these were selected to allow binary responses rather than a distinct experimental condition, leading to fewer animal trials and preventing direct comparisons. Animals were chosen over other objects like tools or vehicles to minimize low-level cue reliance, but share facial features (e.g., eyes) that may obscure face-specific distinctions. Future research employing diverse ROIs, stimuli, and tasks might better address category-specificity^56^.

Secondly, the sequence of events within each trial (baseline → self-regulation → detection/recognition task → delayed intermittent feedback) introduced the possibility of non-specific carryover effects from the regulation phase into the task phase due to the sluggish nature of the BOLD response, potentially complicating the interpretation of task-related responses. However, this sequence was fixed across all conditions, and the observed effects seem to reflect meaningful condition-specific modulations, consistent with our theoretical assumptions. Moreover, our path analyses demonstrated that task-evoked activity in the ROIs independently influenced performance, even after accounting for more global regulation activity.

Thirdly, our differential feedback approach did not explicitly control for potential OFA downregulation during FFA training (nor vice versa). However, we observed *increases* in OFA activity during FFA session and a pattern of rising FFA dominance in the EXP group, suggesting effective FFA upregulation during NFB.

Finally, we combined detection and recognition in the same trials, possibly engendering cognitive and functional overlaps. Despite statistical adjustment for detection responses, unaccounted residual influences might have subtly impacted recognition performance, particularly on slower trials.

Our study employs the use of real-time NFB to regulate two adjacent cortical areas within the same network, FFA and OFA, in order to dissect their respective cognitive function. We demonstrate that successful NFB training enhances the targeted area’s recruitment during a subsequent visual task, leading to distinct behavioural changes in face processing. Specifically, face detection benefits from increased FFA activity coupled with reduced OFA activity, while face recognition is optimized with concurrent activation of both regions. Thus, although OFA lies at an earlier anatomical position along the ventral visual stream and preferentially extracts local feature information, it does not appear to mediate an initial perceptual stage of face detection but rather contribute to later processes subserving more precise face identification abilities. These results contradict a classic hierarchical model positing a posterior (OFA, detection) to anterior (FFA, recognition) local to global processing flow^13,15^, but align with a non-hierarchical perspective^32^, whereby FFA expedites face detection and both FFA and OFA jointly facilitate recognition. Remarkably, simultaneous activation in both regions amplifies recognition efficiency, suggesting more effective integration of OFA inputs with active FFA processes.

These data illuminate neuropsychological mechanisms of clinical deficits in prosopagnosia, and underscore how optimal cognitive performance requires a coordinated recruitment of multiple brain areas but suffers from imbalance among them after injury. More generally, our work highlights the utility of rt-NFB in probing cortical nodes within brain networks, helping elucidate the functional architecture of visual cognition and resolve discrepancies among theoretical models.

## Methods

This research complies with all relevant ethical regulations. The study protocol was approved by the Ethical Committee of Geneva University Hospital (Hôpitaux Universitaires de Genève, HUG), and all participants gave written informed consent prior to participation.

### Participants

Twenty-two right-handed healthy volunteers were recruited and assigned to the experimental (EXP group; female *N* = 14, *Mean* age = 24, *SD* = 3.46, range = 19–34). To control for non-specific factors (e.g., practice effects, habituation) and ascribe regulation effects more confidently to NFB training, we included a control group (CONT group) that comprised 20 matched participants (CONT group; female *N* = 9, *Mean* age = 21.3, *SD* = 2.93, range = 18–32). The latter participated in an identical training regimen but received sham feedback, copied from data of one participant in the experimental group (yoked feedback). Besides general MRI contraindications, exclusion criteria were prior experience with NFB, pregnancy, medication intake, or neurological and/or psychiatric history. All 42 participants had normal or corrected to normal vision and received a small monetary reimbursement for their time. Sex was self-reported by participants.

#### Briefing

For all participants, the first session started with a short (semi-structured) briefing on fMRI-based NFB principles and the study design. They were blinded to their group membership (EXP or CONT) and current target region (FFA or OFA).

### Experimental protocol

#### Stimulus presentation

Visual stimuli and response recording in all tasks (functional localizer, familiarization task, NFB-experiment) were controlled using Psychtoolbox (http://psyschtoobox.org) within the MATLAB environment (Mathworks, Natick, MA, USA).

### Pre-training

#### Functional localizer task

To identify face-responsive FFA and OFA, we used a previously validated protocol^68^ with black-and-white images of neutral faces, fearful faces, houses, scrambled faces, and oval textures. Short blocks of 10 images from the same category (500 ms display time, 50 ms inter-stimulus interval) were repeated 8 times, resulting in 40 blocks, 8.5 min total duration. A 3 s inter-block interval was added to allow for BOLD signal decay. Participants had to press a button whenever a stimulus was presented twice in a row (1-back). The right FFA and OFA were delineated by contrasting different stimulus categories as described elsewhere^68^: (faces fear + neutral faces) > (houses + scrambled faces) and (fear faces + neutral faces) > (textures + scrambled faces), respectively.

#### Familiarization task

Prior to NFB training, participants viewed a set of 18 faces (9 female) and 7 animal images that were randomly drawn from our compiled stimulus set (see: *Stimuli for the Visual Task*). These 25 stimuli were consecutively displayed at the screen’s center for 1500 ms with a 500 ms interval, in pseudo-randomized order. Each stimulus was shown three times over 75 trials (total 2.5 min). Six pictures (3 female faces, 3 animals) were randomly selected as 1-back targets for consecutive presentation. Participants pressed a button for any immediate repetition without explicitly memorizing the images.

### Neurofeedback training

Every participant underwent two distinct training sessions on consecutive days, separately targeting FFA or OFA dominance based on differential NFB signal (FFA-OFA vs OFA-FFA) (see: “Real-time fMRI setup and feedback computation“). Each NFB session consisted of 7 runs (7 trials per run, 49 trials in total, see Fig. 1A) with every trial comprising 4 successive phases (60 s phase duration): baseline (20 s), regulation (25.6 s), visual task (10 s), and intermittent feedback (3.2 s). To allow the feedback signal to stabilize, the first baseline measurement of each run was slightly longer (125 s for run 1 and 30 s for runs 2 to 7, resulting in total duration ~8 min run 1 and ~7min 30s run 2 to 7). Participants trained NFB for ~55 min in total. At the onset of each run, participants were briefly probed on their current motivation with a question presented on the screen: “How motivated are you?”. They answered by moving a horizontal slider between 0 (not motivated) and 100 (strongly motivated), shown below the question.

#### Baseline

During each baseline period, the word “COUNT” was displayed on the screen and participants were instructed to silently count down from 100, at their own pace, while fixating the word. The importance of counting (to obtain a consistent baseline measure) was emphasized during briefing.

#### NFB-based self-regulation

During NFB blocks, participants were presented with a continuous feedback display featuring a red horizontal bar that moved between a central white dot and a black horizontal bar at the top of the screen. This “thermometer” feedback was explained to participants as representing real-time activity in the target brain area. Participants were instructed to reliably raise its “temperature” by identifying effective mental strategies. They were encouraged to improve their performance over time and to explore new strategies if their performance dropped. To facilitate learning, we advised that any “face related visualizations could be a good initial strategy”, emphasizing that they were “free to explore any strategies and use those that worked best for them”. Participants were also informed that effective strategies in the first session may not be similarly effective in the second due to the different target ROI (e.g., aiming at FFA > OFA in session 1 and OFA > FFA in session 2). They were also instructed to stay awake, breathe normally, and fixate on the central fixation dot.

#### Face perception task

Following the NFB regulation period, participants were directly presented with a single trial of a visual task to assess any subsequent effects on face detection and recognition. Using dynamic visual stimulation^42^, participants saw a rapid stream of 200 successive images (48 ms display time, no inter-stimulus interval) with gradually decreasing noise intensities (see Stimuli), progressively revealing the image of face or animal (67 and 33% of trials, respectively) (Fig. 1B). We chose animals as control images to avoid easy discrimination based on low-level visual features (e.g., lines and edges). These control stimuli were included to enable binary perceptual decisions during rapid visual presentation and not considered as a distinct experimental condition (to keep scanning time and fatigue with tolerable limits). Participants were instructed to press one of two buttons as fast as possible when (1) they were sure to detect either a FACE or an ANIMAL, and (2) they recognized whether the image was from the familiarization set (OLD) or a new image (NEW). The corresponding response options were written below the bottom left and right corners of the screen for the entire duration of the trial. The side of response options (left or right) was counterbalanced across participants but fixed throughout both sessions. Subjects were told that only one image was presented on each trial, but that they could vary in size and spatial location (to discourage low-level strategies). After the recognition response was made, the image sequence ran until all 200 images were displayed, and the image was fully revealed.

#### Intermitted feedback

In addition to the thermometer feedback during regulation periods, an intermittent feedback score was provided at the end of each trial (3 s) to allow participants to better track their overall success over time. This score was calculated by taking the sum of all successful NFB moments during the last regulation period (see technical details below).

#### Stimuli for the face perception task

We first compiled a pool of 130 target stimuli (96 faces and 34 animals) selected from two validated sets of high-resolution photographs. We used 60 neutral-expression faces (30 male) from the Umea University Database of Facial Expressions (UUDFE)^69^, featuring predominantly Caucasian but also Arabic and Asian models. Additionally, we incorporated 36 (24 male) mixed-race, (semi)frontally viewed faces and 34 animal images (e.g., birds, horses, cats, etc.) from the Natural Face and Object Stimuli (NFOS)-set^70^. These images were cropped to remove any external features like hair, converted to grayscale, standardized in size, luminance, and contrast, and placed against a uniform white background of 130 × 185 pixels. Luminance was normalized using the SHINE toolbox^71^

From this pool of 130 images, we pseudo-randomly compiled a unique stimulus-set (70 faces (35 male) and 28 animals) for each participant, counterbalancing sex and origin-set (i.e., UUDFE and NFOS). We divided these 98 stimuli into two equal sets of 35 faces (half male) and 14 animals, each tailored for one of the two NFB sessions. Every stimulus was then embedded into one of 25 background images, with the centre of mass positioned at one of 8 possible locations around an imaginary circle on the screen (Fig. 1B), maintaining a balanced distribution of stimuli positions per session. Backgrounds (700 × 700 pixels) were created by fully randomizing the phase information of a single source image.

To gradually increase target visibility (face or animal) during NFB training, a sequence of 200 image-frames with two different sources of visual noise was generated from each of the 98 stimuli. For the first noise source, a 2-D Gaussian filter of decreasing kernel size (standard deviation: 10 to 0 in steps of 0.083) was applied to the first 120 frames in order to avoid any early pop-out effect of high spatial frequency information contained in faces (e.g., eyes). Secondly, for the entire set of 200 frames, we gradually reintroduced clarity through phase-scrambling by incrementally increasing the interpolation strength of the original phase spectra while decreasing the interpolation strength of the random phase spectra from 20 to 75% in steps of 0.37% (for a similar approach^42^).

### Technical details

#### NFB setup

##### Preprocessing with prepNFB Tool

Our NFB protocol was run with the OpenNFT suite (http://opennft.org/)^72^. To simplify and automate preparatory steps prior to NFB, we developed the prepNFB toolbox (https://github.com/lucp88/prepNFB), which uses both custom made and adapted SPM12 functions (Wellcome Trust Center for Neuroimaging, Queen Square, London, UK). Among other features, prepNFB was set up to (1) create the EPI motion correction templates used by OpenNFT for real-time realignment of fMRI data^72^, (2) run and analyse the functional localizer task, (3) perform the subsequent ROI delineation (session 1) and coregistration (session 2) routines.

##### ROI selection with analyse localizer tool

Using the inbuilt localizer tool of prepNFB, raw dicom images were imported as nifti files into a dedicated directory right after completion of the localizer task, spatially realigned to the first volume, and smoothed with an isotropic Gaussian kernel with a 6 mm FWHM. Beta estimates were then obtained for each of the 5 stimulus categories (neutral faces, fear faces, houses, textures, white noise/scrambled faces) by fitting a general linear model to the voxels’ TS. To identify our two target regions-of-interest (ROIs), we contrasted [neutral faces + fear faces] > [houses + scrambled faces] for the FFA, and [neutral faces + fear faces] > [textures + scrambled faces] for the OFA, in each individual^68^. Both activation maps were loaded in the prepNFB “ROI Tool” where they were superimposed on the participants’ structural scan at an uncorrected statistical threshold of *p* < 0.001. Then, we visually located active clusters for each ROI in the *right* hemisphere using a three-step routine:

1. We first selected the highest peak in fusiform (FFA) or inferior occipital gyrus (OFA) plus its 100 neighbouring voxels. If the cluster size was smaller than 100 but greater than 50 (our final ROI size), we accepted this reduced volume and applied it to the other ROI from this participant. If the cluster size was less than 50, we increased it by lowering the statistical threshold (e.g., *p* < 0.005). In case of poor spatial specificity (i.e., too large clusters), we reduced the clusters by applying a correction for multiple comparison (FWE < 0.05).
2. We visually inspected the resulting volume to rule out any overlap or fragmentation. If this was the case, we repeated the step 1 with a smaller number of voxels (but never less than 55) and/or increased the statistical threshold (FWE, *p* < 0.05).
3. Using the thresholded volume obtained from the step 2, we created binarized FFA and OFA ROI nifti files where we assigned a value of one to the 50 most active voxels and nulled the remaining voxels. An automated check was implemented to ensure that ROIs did not overlap.

To ensure the same regions were targeted during the second session, we used prepNFB to coregister the ROIs (delineated during session 1) to the new head position in the scanner on day 2 (session 2). The motion correction template and ROI files from session 1 were coregistered to the MC template of session 2 (generated from the first 25 volumes of the resting state sequence). The resulting coregistered ROI-masks were then used for NFB during session 2.

### Real-time fMRI setup and feedback computation

Real-time fMRI processing, feedback computation, and data visualization was implemented within the OpenNFT environment (http://opennft.org/)^72^, running on a processing unit (Intel Core I9, 128GB RAM, Windows 10). To optimally incorporate the face perception task into the NFB-protocol, a dedicated task module was incorporated into the NFB estimation scheme.

#### Whole-brain real-time data processing

During NFB, the acquired and reconstructed dicom images were exported in real-time and sent over an Ethernet connection to a shared destination folder on the processing unit. Data volumes were imported by OpenNFT, spatially realigned to the EPI motion correction template, resliced, and smoothed with a 5 mm FWHM Gaussian kernel.

#### Real-time time-series processing

To compute real-time feedback, the average signal of each ROI was extracted from the smoothed whole-brain data, filtered with an autoregressive model AR(1), and entered in a cumulative GLM with separately modelled regressors of NFB regulation and visual task periods (convolved with hemodynamic response function), as well as head motion, linear trend, and constant covariates. The constant GLM beta estimate was added back to the time-series after it was additionally filtered with a low-pass modified Kalman filter^73^. As GLM-based parameter estimations are notoriously noisy with insufficient data points, we (1) extended the first baseline block of each run (run 1 = 115 volumes/92 s, run 2 to 7 = 46 volumes/37 s), and 2) included data from the last half of each preceding run to the cumulative GLM estimation routine (runs 2 to 7).

#### Neurofeedback signal computations

For both ROIs, the filtered time-series were used to estimate the incremental percent signal change (PSC) values relative to an accumulated baseline median:

1 $${{PSC}}_{v}=\frac{{{mean}}({ROI}_{{Reg}(v-2:v)})-{median}({{ROI}}_{{AccBas}})}{{median}({{ROI}}_{{AccBas}})}$$

where *v* is the current time point, ${{ROI}}_{{Reg}}$ is the region’s activity during self-regulation and ${{ROI}}_{{AccBas}}$ is the accumulated baseline measurements since the start of the run. To account for sudden changes in the signal, the two previous regulation values were averaged with the current regulation value (*v−2:v*). Of note, as the beginning of each baseline block may contain residual activity from the preceding DVS and feedback presentation blocks, only the last 10 volumes (8 s) of baseline blocks were considered for PSC computations. We then computed the differential PSC between the target and non-target regions:

2 $${{diffPSC}}_{v}={{tPSC}}_{v}-{{ntPSC}}_{v}$$

While a differential NFB signal does not explicitly control for downregulation of the non-target region, extensive piloting and subsequent results (see main text) showed this to be a negligible factor. More critically, this approach allowed us to induce a relative bias in favor of one or the other ROI during their joint engagement during the task. By regulating this relative bias (ROI dominance) rather than mean activity in a single ROI, we were able to isolate the specific contribution of each region to the immediately following task of face detection and/or recognition. Moreover, because OFA and FFA display correlated activity during both rest and face processing tasks^74,75^, taking co-activation in the non-target region into account was essential. In addition, such a bidirectional design has been advocated to control for spurious factors such as motivational biases and placebo effects^76^ and controls for global effects like breathing or movement artifacts as they are cancelled out and do not alter the feedback signal.

The differential PSC was dynamically scaled to individual performance level up to the current point ${{limit}}_{{up}},\,{{limit}}_{{low}}$) and visualized as a temperature on feedback thermometer, ranging from the central fixation point to 420 pixels above the fixation point:

3 $${t}_{v}=\frac{{{diffPSC}}_{v}-{{limit}}_{{low}}}{{{limit}}_{{up}}-{{limit}}_{{low}}}*\left({{step}}_{\max }-{{step}}_{\min }\right)+{{step}}_{\min }$$

where *t* is the thermometer temperature, *v* is the current time point, *limit up/limit low* are the median of the five highest/lowest *diffPSC* values since the onset of the run, and *step max/step min* are the screen coordinates at the fixation dot (*y* = 0) and the top of the thermometer (*y* = 420). Importantly, Eq. (3) was only evaluated when PSC of the target ROI was larger than that of the non-target ROI (i.e., the negative *diffPSC* values defaulted to 0 and no thermometer increase), which ensures overall positive feedback of the more active target ROI. To obtain an intuitive feedback range, the intermittent feedback score presented at the end of each trial was scaled:

4 $${{IFB}}_{t}=\frac{{sum}(T)}{{{step}}_{\max }}*10$$

where *T* are the temperature readings of the most recent regulation block, and *step max* is the maximum temperature of the thermometer (420).

### Control group (CONT)

For the yoked control group, each subject was assigned to temperature readings, intermittent feedback scores, and stimuli/trial types taken from another randomly selected participant in the experimental group. Once used as a “yoke”, the experimental participant was discarded from further pairing to other control subjects.

### Image acquisition

MRI images were acquired using a 3T MRI scanner (TrioTIM, Siemens, Germany) with a 32-channel head coil at the Brain and Behavior Laboratory in Geneva University.

#### Functional images

For all tasks (functional localizer, resting state and NFB) were acquired with a single-shot gradient-echo T2*-weighted EPI sequence (44 slices, matrix size = 64 × 64, voxel size = 3 × 3 × 2.5 mm^3^, slice gap = 0.5 mm, flip angle α = 52˚, bandwidth 2004 Hz/Px, TR = 800 ms, TE = 30 ms).

#### Anatomical scans

A high-resolution T1-weighted anatomical scan was acquired (3D MPRAGE, 256 × 256 × 192, voxel size = 1 mm isotropic, flip angle *α* = 9°, bandwidth = 190 Hz/Px, TR = 1900 ms, TI = 900 ms, TE = 2.27 ms) and used to project the real-time whole-brain statistical results on during NFB runs.

### Analyses

#### Offline analysis of ROI time-courses

Raw FFA and OFA time-series were extracted by OpenNFT and processed offline using the same approach as above, but regressors of interest and covariates were now used for GLM filtering in their entirety (as opposed to incrementally). From these processed time-courses, *trial-wise* mean PSCs were computed using Eq. (1) (as opposed to the moment-by-moment computations during NFB), where the average of the last 10 volumes (8 s) of each *regulation* block was compared to the accumulated median baseline. Equation 2 then calculated the differential PSC for each trial separately.

Separate analyses were run to estimate evoked ROI activity during face/animal detection and recognition for each trial and each individual. Here, GLMs included trial-wise 5 regressors of interest (i.e., detection, recognition, baseline, self-regulation and IFB) and 6 covariates derived from head movement parameters.

### Offline analysis of whole brain data

#### Software

Preprocessing and statistical analyses of the anatomical and functional data were carried out within the Nipype framework^77^ using Python (version 3.8) in combination with the integrated development environment Spyder^78^.

#### Preprocessing

For anatomical scans, cortical reconstruction was performed on each participant’s data using FreeSurfer’s “recon-all” process (http://surfer.nmr.mgh.harvard.edu/). We used ANTs “antsRegistration”^79^ to obtain the transformation matrix for normalization by computing the registration between the participants’ segmented brain and the MNI-template. Preprocessing steps for functional data included despiking, realignment, removal of the second order polynomial fit, artifact detection, and smoothing. Despiking was performed using the AFNI software package^80^, while realignment to the mean was performed using SPM12 (http://fil.ion.ucl.ac.uk/spm/). Temporal signal-to-noise ratio was calculated using the TSNR tool and with a polynomial regression of the second order to remove low-frequency drifts. Artifact detection was performed with the RapidArt tool, and final image smoothing was performed using SPM12 (kernel size FWHM = 8).

#### First- and second-level analyses

First-level analyses were conducted within the Nipype workflow using SPM12 nodes. Contrast images were generated to evaluate brain regions that were more active during regulation compared to baseline (regulation > baseline). These contrast images were coregistered to the anatomical data and normalized to MNI standard space using ANTs apply transform routine, before being taken to the second-level (group) analyses. The resulting parametric maps were corrected for multiple comparisons (FWE < 0.05) and rendered on an MNI-template brain (MNI152) using nilearn^81^

#### Overlay mapping

To identify any overlap between brain activation produced by self-regulation during NFB and face-responsive cortical areas, we computed an overlay map between these two comparisons. This was achieved through the co-registration and superimposition of two distinct brain activation maps. NFB increases were determined by contrasting regulation > baseline periods in each run, while face perception activity was determined from the localizer scans, where we contrasted faces (fearful + neutral) vs. control stimuli (houses + scrambled faces). Both sets of activation maps were then superimposed on an MNI-template brain using MRIcroGL^82^. This process was aided by colour coding to clearly delineate the areas of activation from each dataset and their overlap. It is important to note that this approach allowed us to identify regions of anatomical overlap, but it did not employ statistical methods to formally test for the significance of these overlaps.

### Face perception task performance

To evaluate behavioural performance during the visual task following each NFB run, we recorded the DVS frame number when a detection/recognition response was made (by key press). Since fast behavioural response latencies occur early in the DVS-stream, they correspond to low frame numbers, whereas slow responses correspond to higher frame numbers. These trial-wise detection/recognition frame numbers were analysed to assess the speed of face/animal detection and recognition. Response times were recorded in milliseconds. All trials were included in the analyses, irrespective of response accuracy, as performance was generally high, allowing us to maximize and compare conditions with equal and maximal statistical power. Control analyses performed for the two main behavioural models (LMM 6 and 7) after exclusion of error trials (more frequent for recognition than detection) showed no meaningful changes to the results or interpretations.

### Self-regulation performance

#### Session effects

To investigate the effects of NFB on brain regulation performance, we first computed trial-wise (baseline, regulation, task) averages from the rFFA/rOFA time-courses for each participant and each training session (four time-courses per subject, pooling across all runs from one session). For each ROI, we compared the average self-regulation activity in the EXP group relative to the CONT group during each training session, resulting in four pairwise (between group) comparisons. Secondly, for each group, we compared ROI-specific self-regulation activity during FFA vs OFA sessions, resulting again in four pairwise (within group) comparisons. For each pairwise comparison, an independent (between group) or paired sample (within group) *t*-test was computed at each time point of the time-course and then compared against an empirically generated t distribution. These distributions were obtained from randomly permuting group membership (between group comparisons) or permuting the sign of a random subset of data points (within group comparisons). Permutation number in both cases was set to 10.000 times.

#### Linear mixed-effects models

To investigate self-regulation learning (Model 1), the influence of self-regulation on stimulus-evoked ROI responses (Models 2–5), and the influence of stimulus-evoked ROI responses on behavioural performance (Models 6–7), we fitted a series of linear mixed-effects models (LMMs) to trial-by-trial data using the lme4 package^83^ with Satterthwaite degrees of freedom (lmerTest) in R (version 2023.03.0 + 386). In all models, participants and stimuli were included as random intercepts to account for repeated measures within subjects and stimulus-specific variance. Binary predictor variables were deviation-coded (−0.5/+0.5), so that main effects reflect the average effect of a predictor across levels of other factors in the model. We report standardized LMM coefficients (*ß*) in the main text and in dot-whisker plots, where each coefficient is scaled by two standard deviations of its predictor to allow visual comparison of effect magnitudes across predictors. Marginal-effect plots show unstandardized LMM-predicted values in original units with 95% confidence bands. To decompose significant interactions, we computed post-hoc simple slopes using emtrends (emmeans package), evaluated on the unstandardized model in original units; corresponding estimates are annotated on the marginal-effect panels. These post-hoc tests were corrected for multiple comparisons within each model family using the Holm method; the number of tests per family is stated with each figure.

#### NFB-learning

To assess whether experimental participants gained increasing control over their differential ROI activity, we fitted an LMM (Model 1) with the trial-wise difference in PSC between FFA and OFA as the dependent variable. The fixed factors were run, session, group, and training day. A three-way interaction between run, session, and group was included to test whether within-session learning in the EXP group exceeded that of the CONT group.

### The effects of self-regulation performance on ROI detection and recognition responses

To examine whether FFA/OFA activity during self-regulation modulated subsequent task-evoked activity in these regions, we constructed four more LMMs. Models 2 and 3 assessed how regulation was associated with *face detection* responses, while models 4 and 5 assessed how regulation was associated with *face recognition* responses. We use the abbreviations “*regFFA*“ and “*regOFA*“ to denote percent signal changes in FFA and OFA during regulation, respectively.

In all four models, the fixed factors were trial-wise *regFFA* and *regOFA*, *Session*, *Run number, Detection latency (frames)*, and *Training day;* while the interaction between *regFFA* × *regOFA* × *Session* was included to investigate whether the effect of regulation activity on subsequent detection/recognition activity was differentially modulated as a function of the other ROI and training session. In the recognition models (4 and 5), we additionally controlled for the preceding FFA/OFA *detection response*.

The dependent variable of each model was defined as the trial-wise beta estimates related to the ROI-specific face detection and recognition responses. For instance, a positive association between *regFFA* and *FFA detection activity* would suggest that higher FFA activity during self-regulation is associated with enhanced FFA detection activity in the subsequent task phase, consistent with a potential carry-over or priming effect.

Both *participants* and *stimuli* were included as random (intercept) effects. For simplicity, the control variables: *Run number*, *Training Day*, *detection frames*, and *detection response* are not displayed in the main results (Fig. 5) but reported in the supplementary materials (Supplementary Fig. 3). To facilitate the interpretation of main effects in all four LMMs, binary predictor variables were factorized, and effect coded by assigning values of −0.5 and +0.5 to the respective categories. Such deviation coding allows interpreting the main effects as the average effect of the predictor variable across all levels of other predictors in the model, assuming no interaction.

### The effects of ROI detection/recognition responses on response latencies

To investigate the relationship between FFA/OFA activity evoked by face detection or face recognition and corresponding behavioural performance, one LMM was computed for latencies of each response category. The dependent variable, *performance*, was defined as the trial-wise response latencies in image frames (see above) for either face detection (LMM 6; blue) or face recognition (LMM 7; red). The fixed factors were the trial-wise evoked responses in *FFA* and *OFA*, as well as *training session* and *training day*. Three-way interaction terms between evoked responses in *FFA* and *OFA*, and *training session* were included to evaluate whether the effect of one region on response latencies was influenced by the co-activation in the other region, and whether there were differences between the two training sessions in this regard. Since *performance* was expressed as response latencies, negative linear effects corresponded to *improved* performance (i.e., fewer image frames and faster face detection or recognition). As the models above, participants and stimuli were included as random (intercept) effects.

We analysed stimulus-evoked responses in the task-phase separately from the regulation-phase to understand how FFA and OFA activation during the task directly relates to behavioural outcomes, independent of prior modulation. However, these analyses do not fully disentangle the contributions of regulation-induced and task-evoked activity. To address this, we conducted a path analysis (next section) integrating both phases into a single framework, allowing us to evaluate their distinct and combined effects on performance.

### Path analysis using structural equation modelling

To investigate how the NFB training (session) modulated self-regulation activity (PSC) in both FFA and OFA, and how subsequent detection/recognition responses in each ROI related to differences in behavioural performance (detection and recognition frames), we tested a more complex model incorporating these different steps (Fig. 7A) using path analysis in the context of structural equation modelling (SEM). Path analysis is an extension of multiple linear regression and allows for the quantification and testing of the overall fit of a pre-defined multivariate system. Such a system is statistically represented as a series of structured linear regression equations which together describe specific and directional relationships between variables (i.e., a directed path diagram as shown in Fig. 7A). In addition, path analysis allows for strength and significance estimation of these individual path coefficients (beta weights) between the variables specified (e.g., X→Y) which are interpreted as predictive terms similar to regular linear regression weights^84^. Finally, path model specification is theoretically informed and may involve defining both direct (X→Z) and indirect (X→Y→Z) “causal” effects between a set of observed variables, whereby causality is assured through the principle of temporal precedence (X occurs before Y). This approach thus permits to consider the effects of self-regulation, stimulus-evoked responses, and behavioural performance jointly within the same analysis.

To specify and test our path model, we used the open-source software package *Semopy*^85,86^. The model was applied to the trial-by-trial data and separately evaluated for the EXP and CONT groups. Besides these *connections of interest*, we also included multiple direct effects and Training Day as a control variable. The full model (depicted in Fig. 7A) was separately evaluated for the EXP and CONT groups and was restricted to data from face trials only. To account for the clustered nature of our data (49 trial observations per participant per session), we used the inbuilt “ModelMeans” method of semopy, which implements a subject-wise centring of the data before fitting the model. The maximum likelihood function was used for parameter estimation and model fitting. To evaluate the goodness of fit of the models, we focused on the Root Mean Square Error of Approximation (RMSEA) in combination with the chi-square goodness of fit statistic. The RMSEA is a “badness of fit” index where 0 indicates a perfect fit and higher values indicate poor fit. Values below 0.10 are acceptable, while values below 0.06 indicate a good fit^87,88^. The ${X}^{2}$ test statistic is used to evaluate the appropriateness of the specified model by comparing the resulting model-covariance matrix with that of the sample covariance matrix. As such, a non-significant difference indicates that the specified model adequately represents the empirical data. Once the models were evaluated, we assessed the standardized estimates of significant individual paths between variables to determine putative mechanistic influences between them.

### Reporting summary

Further information on research design is available in the Nature Portfolio Reporting Summary linked to this article.

## Supplementary information

Supplementary Information

Reporting Summary

Transparent Peer Review file

## Source data

Source Data

## Data Availability

The source data underlying all main and Supplementary Figs. are provided in the Source Data file published with this paper. The individual-level neuroimaging and behavioural data generated in this study are available under restricted access due to participant privacy protections under the ethical approval granted by the Ethical Committee of Geneva University Hospital (HUG), which does not permit unrestricted public deposition of identifiable neuroimaging data. Access can be obtained by contacting the corresponding author (lucas.peek@unige.ch) and completing a data use agreement specifying the intended use. Access requests will be reviewed and responded to within 30 days, subject to ethical approval. Data are available for academic, non-commercial research purposes. Data will remain available for a minimum of 10 years following publication. Source data are provided with this paper.

## Code availability

The prepNFB toolbox developed for this study is available at https://github.com/lucp88/prepNFB. The real-time fMRI neurofeedback protocol was implemented in a custom version of the OpenNFT suite (http://opennft.org/). Analysis code (R and Python) for reproducing all statistical analyses and figures (main Figs. 2–7 and Supplementary Figs. 1–9), including linear mixed models, permutation tests, and structural equation models, is publicly available at https://github.com/lucp88/NCOMMS-24-11279A and archived on Zenodo (10.5281/zenodo.19548058). All other software and tools used in this study are described in the “Methods” section.
