# Supplementary material for: Trial-by-trial fMRI-neurofeedback dissociates fusiform and occipital contributions to face detection and recognition
Source: Nat Commun. 2026 Jun 13;17:7515. doi: 10.1038/s41467-026-74331-2 (PMC13409027; doi:10.1038/s41467-026-74331-2)
Supplement: Supplementary file 1 — Supplementary Information [file 41467_2026_74331_MOESM1_ESM.pdf]

Supplementary Materials

Trial-by-trial neurofeedback dissociates fusiform and occipital contributions to face detection and recognition

NCOMMS-24-11279

Table of Contents

Supplementary Figures	Page
**Supplementary Figure 1. First vs last regulation run, between-session comparison within the EXP group**<br>Time-courses showing FFA session-specific modulation emerges only in the final regulation run.	1
**Supplementary Figure 2. Raw data and model diagnostics for LMM 1**<br>Individual learning slopes and residual diagnostics validating the statistical modelling approach.	2
**Supplementary Figure 3. Detailed tabulation of Linear Mixed Models 2–5**<br>Full model output for self-regulation effects on task-evoked FFA and OFA activity.	3
**Supplementary Figure 4. Self-regulation effects on animal detection and recognition**<br>Control analysis applying the same models to animal stimuli, confirming face-specificity.	4
**Supplementary Figure 5. Behavioral performance**<br>Response latencies for detection and recognition by stimulus category, session, and group.	4
**Supplementary Figure 6. Detailed tabulation of Linear Mixed Models 6 and 7**<br>Full model output linking task-evoked ROI activity to behavioral detection and recognition speed.	5
**Supplementary Figure 7. Full path model output for the EXP and CONT groups**<br>Structural equation model path diagrams with all regression coefficients and covariances.	6
**Supplementary Note**	
**Supplementary Note: ROI specificity**<br>STS control analyses demonstrating that neurofeedback effects are specific to targeted regions.	7–9
**Supplementary Figure 8. STS control analysis: time-course and learning**<br>STS time-course and LMM 1 with STS as DV, showing no selective session-specific upregulation.	7
**Supplementary Figure 9. STS control analysis: LMMs 6 & 7 with STS predictor**<br>Behavioral models extended with STS activity; key FFA × OFA interactions remain significant.	9

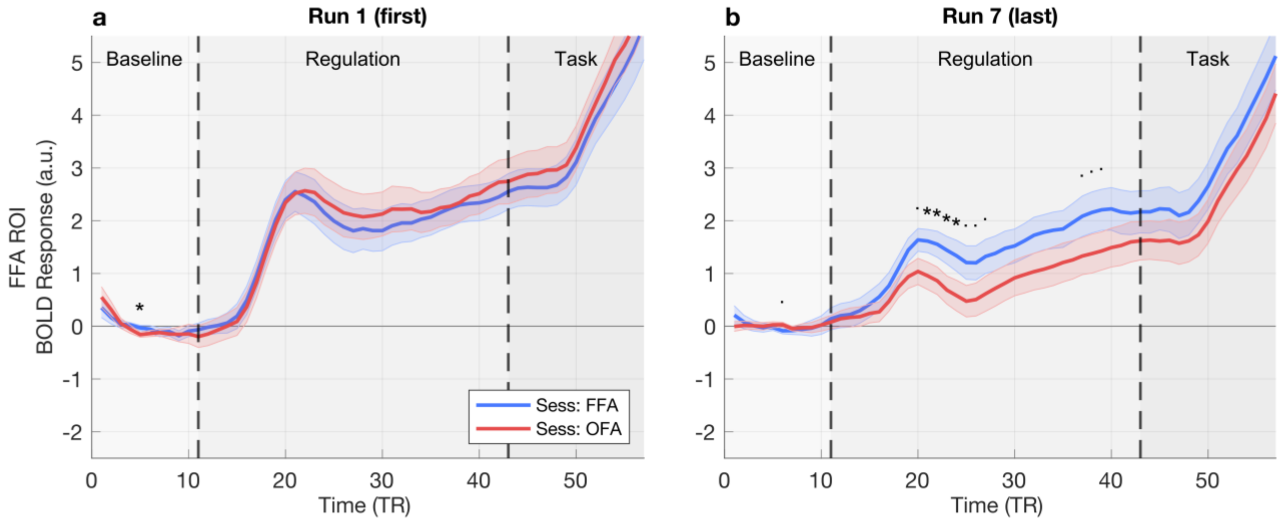

Supplementary Figure 1. First vs last regulation run, between-session comparison within the EXP group. Time-courses of BOLD activity (mean ± SEM; baseline-corrected) in the FFA ROI during the initial (Run 1; panel **a**) and final (Run 7; panel **b**) regulation run of each training session. Blue traces = FFA training session; red traces = OFA training session. A differential session-specific response emerges only at the end of training; significantly higher FFA activity during FFA-targeted sessions than OFA-targeted sessions across the regulation phase of Run 7 (**b**), whereas no such contrast is present in Run 1 (**a**).

Shaded error bands indicate ±1 SEM; time-courses are baseline-corrected for visualization. Dashed vertical lines demarcate the baseline, regulation, and task phases. Volume-by-volume permutation results (uncorr.) are marked with asterisks for significant effects and dots for trending effects. Data are presented as mean values ± SEM (n = 22 independent participants in the EXP group).

Statistical analysis: volume-by-volume paired permutation test (sign-flipping on within-subject difference scores; target session minus non-target session (i.e., FFA session − OFA session for the FFA ROI, OFA session − FFA session for the OFA ROI), two-sided, 10,000 permutations, uncorrected for multiple comparisons across time points. Significance at $p < 0.025$ or $p > 0.975$, trending at $0.025 \leq p < 0.05$ or $0.95 < p \leq 0.975$. * $p < 0.05$, · trending. Source data are provided as a Source Data file.

Abbreviations: NFB, neurofeedback; FFA, fusiform face area; OFA, occipital face area; ROI, region of interest; EXP, experimental group; SEM, standard error of the mean; TR, repetition time.

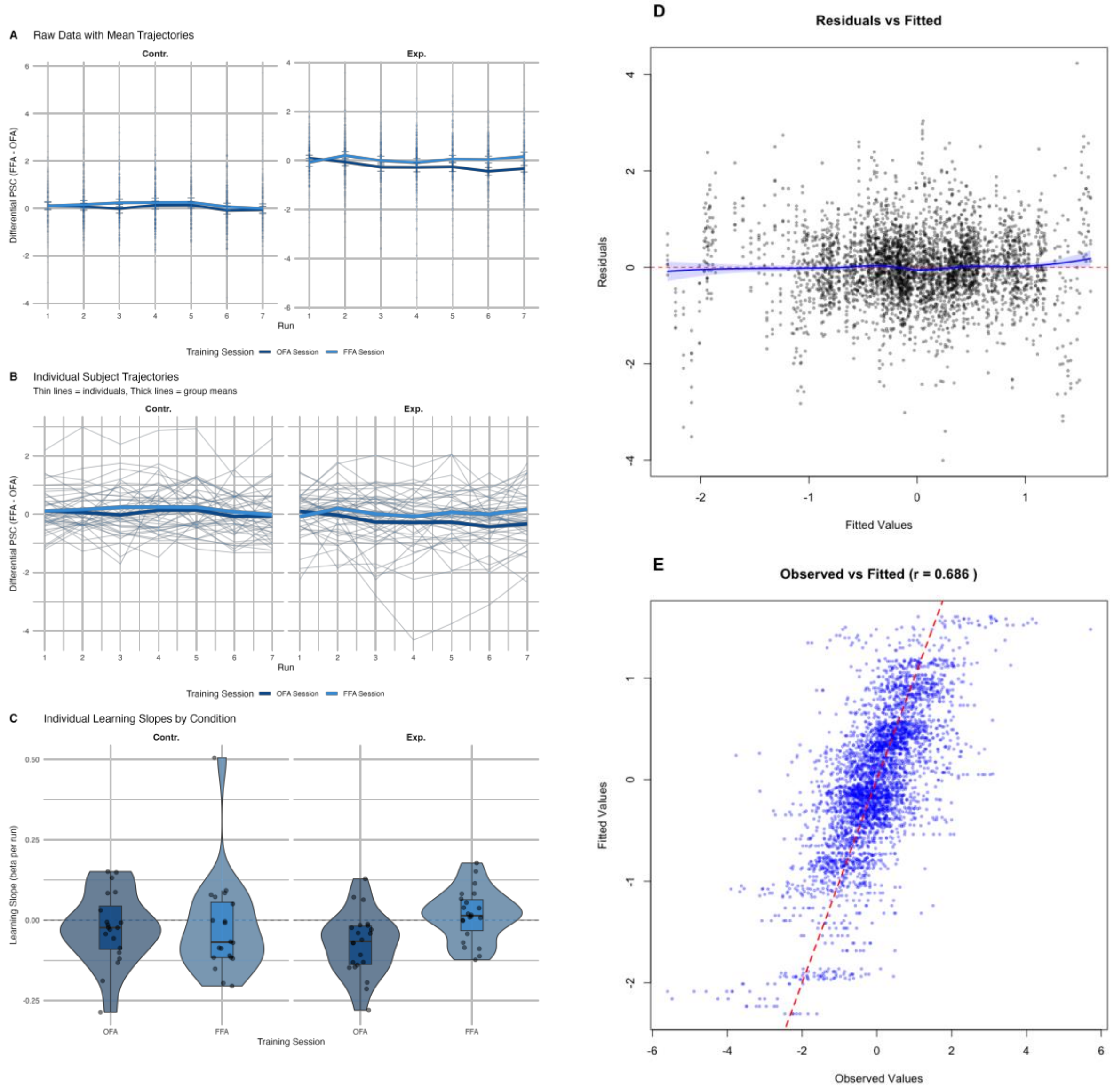

Supplementary Figure 2. Raw data and model diagnostics for LMM 1. Panels A–B show the raw, unmodelled, differential PSC (diffPSC) data for NFB learning effects captured in Linear Mixed Model 1 (Fig. 3, main text), followed by individual learning slopes (C) and diagnostic plots (D–E), which converge to demonstrate the validity of the statistical modelling approach.

A) Each dot represents an individual trial's diffPSC (FFA − OFA), plotted separately for each group (EXP, CONT) and training session (FFA session, light blue; OFA session, dark blue). Thick lines depict group-level mean trajectories per session; error bars represent 95% confidence intervals (±1.96 × SEM). The EXP group (right) shows clear session-contingent divergence: a decrease in differential PSCs during the OFA session (reflecting increased OFA activity) and relative stability or increase during the FFA session. The CONT group (left) shows no such divergence, consistent with the absence of a learning effect.

B) Same data as in A, plotted as individual subject trajectories. Each thin line represents a subject's mean diffPSC across runs in one training session; thick lines reflect group means. This visualization emphasizes individual variability and complements the modelled results by illustrating consistent session-specific modulation in the EXP group, and the lack thereof in the CONT group.

C) Distribution of individual learning slopes, computed as beta coefficients from linear regressions of run number on diffPSC per subject and session. Violin plots show the density distribution; embedded boxplots show the median (centre line), 25th–75th percentiles (box), and 1.5 × interquartile range (whiskers); individual data points are overlaid. In the EXP group (right), slope directions reflect effective learning: positive for FFA training (increased FFA dominance) and negative for OFA training (increased OFA dominance). The CONT group (left) shows no consistent slope direction or session-based separation.

D) Residuals versus fitted values for LMM 1. Residuals are symmetrically distributed around zero across the range of fitted values, supporting homoscedasticity and linearity. Blue line: LOESS smooth with 95% confidence band; red dashed line: zero reference.

E) Observed versus fitted values for LMM 1 (Pearson's r = 0.686), indicating that the model explains a substantial portion o variance in diffPSC. Red dashed line: identity line.

Linear Mixed Models: Self-Regulation → Task Responses

	Model 2: FFA detection				Model 3: OFA Detection				Model 4: FFA Recognition				Model 5: OFA Recognition			
Predictors	*Estimate*	*SE*	*CI*	*p*	*Estimate*	*SE*	*CI*	*p*	*Estimate*	*SE*	*CI*	*p*	*Estimate*	*SE*	*CI*	*p*
(Intercept)	-0.36	0.15	-0.65 – -0.07	**0.015**	-0.62	0.14	-0.90 – -0.34	**<0.001**	0.96	0.13	0.71 – 1.21	**<0.001**	0.79	0.15	0.49 – 1.09	**<0.001**
regFFA s	0.14	0.03	0.07 – 0.20	**<0.001**	-0.01	0.03	-0.07 – 0.06	0.834	0.12	0.03	0.07 – 0.17	**<0.001**	0.04	0.03	-0.01 – 0.10	0.085
regOFA s	0.03	0.04	-0.05 – 0.10	0.487	0.20	0.03	0.13 – 0.27	**<0.001**	0.02	0.03	-0.04 – 0.08	0.480	0.10	0.03	0.05 – 0.16	**<0.001**
Regulation1	0.02	0.05	-0.07 – 0.11	0.639	0.08	0.04	-0.01 – 0.17	0.072	0.01	0.04	-0.06 – 0.08	0.811	-0.14	0.04	-0.20 – -0.07	**<0.001**
runID	-0.03	0.01	-0.05 – -0.01	**0.007**	-0.01	0.01	-0.04 – 0.01	0.166	-0.00	0.01	-0.02 – 0.02	0.931	0.01	0.01	-0.00 – 0.03	0.096
Training Day [linear]	-0.07	0.03	-0.13 – -0.01	**0.030**	-0.03	0.03	-0.09 – 0.03	0.352	-0.13	0.03	-0.18 – -0.07	**<0.001**	-0.07	0.02	-0.12 – -0.03	**0.003**
detection frms	0.00	0.00	0.00 – 0.01	**<0.001**	0.01	0.00	0.01 – 0.01	**<0.001**	-0.01	0.00	-0.01 – -0.01	**<0.001**	-0.01	0.00	-0.01 – -0.01	**<0.001**
regFFA s × regOFA s	0.02	0.02	-0.01 – 0.05	0.118	0.01	0.01	-0.02 – 0.04	0.555	-0.00	0.01	-0.03 – 0.02	0.821	-0.01	0.01	-0.03 – 0.02	0.519
regFFA s × Regulation1	-0.12	0.05	-0.22 – -0.01	**0.027**	-0.15	0.05	-0.25 – -0.05	**0.004**	-0.00	0.04	-0.09 – 0.08	0.960	0.00	0.04	-0.08 – 0.08	0.956
regOFA s × Regulation1	0.05	0.05	-0.06 – 0.16	0.343	0.12	0.05	0.02 – 0.22	**0.024**	-0.04	0.04	-0.13 – 0.05	0.366	-0.05	0.04	-0.13 – 0.04	0.273
regFFA s × regOFA s × Regulation1	-0.02	0.03	-0.07 – 0.04	0.584	-0.07	0.03	-0.12 – -0.02	**0.011**	0.05	0.02	0.00 – 0.09	**0.047**	0.04	0.02	0.00 – 0.09	**0.040**
task detect FFA beta s									-0.60	0.02	-0.65 – -0.56	**<0.001**				
task detect OFA beta s													-0.67	0.02	-0.71 – -0.63	**<0.001**
**Random Effects**																
σ^2	0.68				0.62				0.44				0.39			
τ_{00}	0.00 $_{stimID}$				0.00 $_{stimID}$				0.00 $_{stimID}$				0.00 $_{stimID}$			
	0.26 $_{subID}$				0.23 $_{subID}$				0.20 $_{subID}$				0.38 $_{subID}$			
ICC					0.28											
N	22 $_{subID}$				22 $_{subID}$				22 $_{subID}$				22 $_{subID}$			
	50 $_{stimID}$				50 $_{stimID}$				50 $_{stimID}$				50 $_{stimID}$			
Observations	1494				1494				1494				1494			
Marginal R^2 / Conditional R^2	0.072 / NA				0.101 / 0.349				0.506 / NA				0.580 / NA			

Supplementary Figure 3. Detailed tabulation of Linear Mixed Models 2–5. The models assess the modulation of ROI pre-stimulus activity during self-regulation on subsequent stimulus-driven FFA and OFA activation during face detection and recognition. Each model incorporates fixed effects including regFFA, regOFA, Session, Run number, Training Day, and their interactions. Recognition models (Recog Resp. FFA/OFA) additionally include the preceding detection response of each ROI as a covariate. Control variables (Run number, Training Day, detection frames, detection response) included in the models were omitted for clarity in the main text but are comprehensively presented here.

Estimates are unstandardized coefficients with 95% confidence intervals (CI). Bold p-values indicate statistical significance ($p < 0.05$). Both participants (subID) and stimuli (stimID) are treated as random intercepts. EXP group only, n = 22 independent participants; 1,494 trial-level observations. Statistics were obtained from two-sided LMMs fitted with REML and Satterthwaite degrees of freedom (lme4/lmerTest). Source data are provided as a Source Data file (see Fig. 5 tab). Abbreviations: LMM, linear mixed model; FFA, fusiform face area; OFA, occipital face area; ROI, region of interest; regFFA/regOFA, self-regulation activity in FFA/OFA; Detect Resp, detection response; Recog Resp, recognition response; FRMS, frames (response latency); CI, confidence interval; σ^2, residual variance; τ_{00}, random intercept variance; ICC, intraclass correlation coefficient.

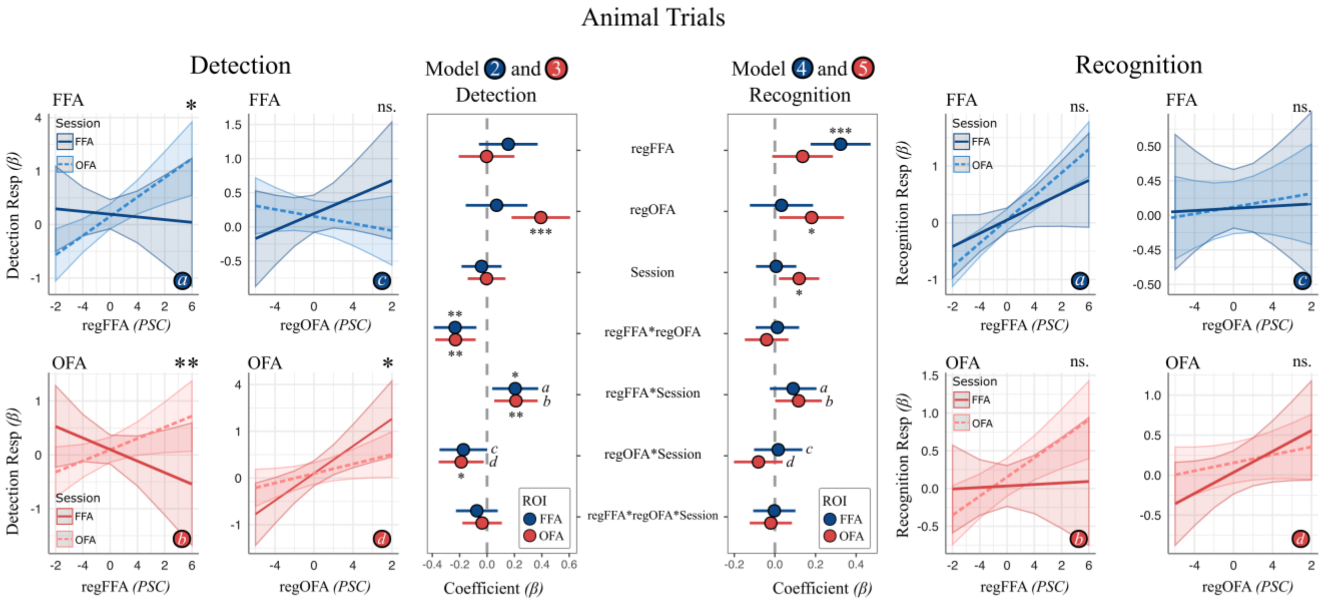

Supplementary Figure 4. Self-regulation effects on subsequent task-evoked activity in each ROI during animal detection and recognition. Same analysis as Figure 5 but applied to animal stimuli as a control comparison. Results indicated a more limited influence of FFA and OFA self-regulation on animal detection and recognition, aligning with their face-specific functions. Dot-whisker plots show coefficient estimates (centre) with 95% CIs (whiskers). Slopes show LMM-predicted marginal effects (lines) with 95% CIs (shaded bands; centre: predicted mean). Marginal plots show density distributions by session. EXP group only, n = 22; 583 trial-level observations (independent participants). Statistics were obtained from two-sided LMMs (fixed effects: regFFA × regOFA × Session, with Run, Training Day, and detection frames as covariates; random effects: participant and stimulus) fitted with REML and Satterthwaite degrees of freedom. * $p < 0.05$, ** $p < 0.01$, *** $p < 0.001$, ns. = not significant. Source data are provided as a Source Data file. Abbreviations: FFA, fusiform face area; OFA, occipital face area; ROI, region of interest; LMM, linear mixed model; CI, confidence interval.

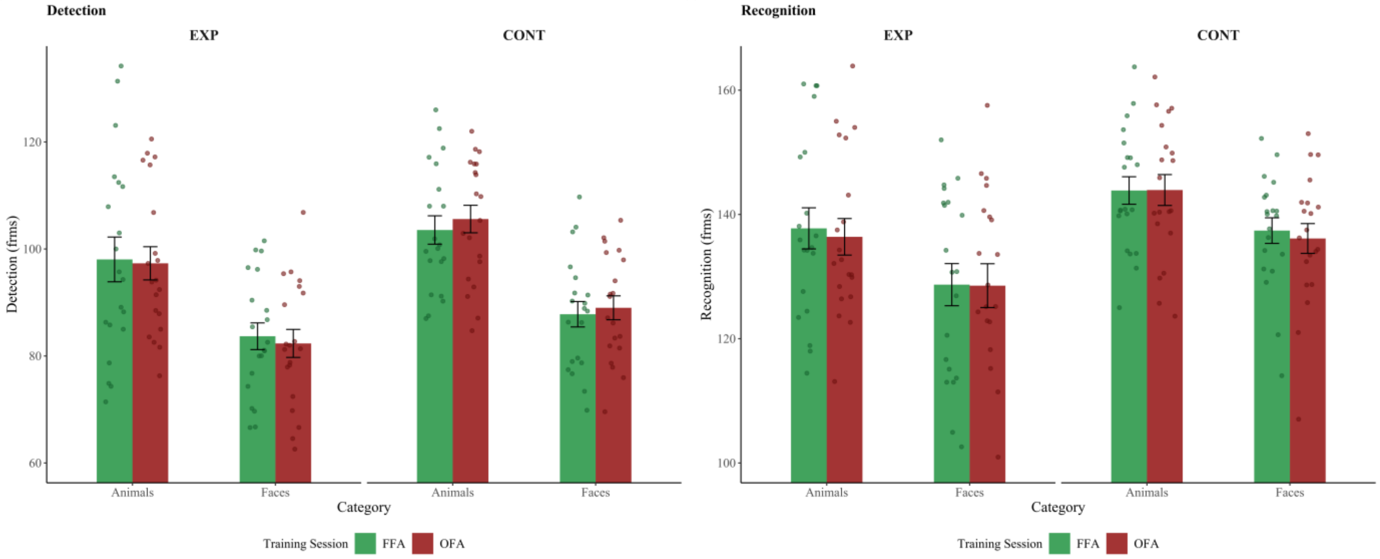

Supplementary Figure 5. Behavioral performance. Response latencies for correct detection and recognition performance, according to stimulus category (Faces, Animals) and training session (FFA, OFA) for both groups (EXP, CONT). Response latencies are expressed in image frames corresponding to the visual noise level at which a stimulus (face or animal) was reported by the participant on a given trial. Bars represent group means (centre) with error bars showing ± 1 SEM. Individual data points represent subject-level means. Two participants were excluded based on outlier detection (rstatix::identify_outliers; values outside Q1 − 1.5×IQR or Q3 + 1.5×IQR on detection latencies), leaving n = 20 independent participants per group (40 total). Statistics were obtained from two-sided mixed ANOVAs (within: Category × Training Session; between: Group) using rstatix::anova_test; full results reported in the main text. Source data are provided as a Source Data file. Abbreviations: FFA, fusiform face area; OFA, occipital face area; EXP, experimental group; CONT, control group; SEM, standard error of the mean; ANOVA, analysis of variance.

Model 6: Detection performance

	Model 6: Detection (unstandardized)				Model 6: Detection (standardized)			
Predictors	*Estimate*	*SE*	*CI*	*p*	*Estimate*	*SE*	*CI*	*p*
(Intercept)	88.57	3.39	81.93 – 95.22	**<0.001**	88.57	3.39	81.93 – 95.22	**<0.001**
FFA Detection	-1.96	0.81	-3.55 – -0.37	**0.016**	-3.92	1.62	-7.10 – -0.74	**0.016**
OFA Detection	5.32	0.83	3.69 – 6.95	**<0.001**	10.64	1.66	7.39 – 13.90	**<0.001**
Session	1.73	0.92	-0.08 – 3.54	0.061	1.73	0.92	-0.08 – 3.54	0.061
Training Day	-1.94	0.59	-3.11 – -0.77	**0.001**	-1.94	0.59	-3.11 – -0.77	**0.001**
FFA Detection × OFA Detection	1.75	0.28	1.19 – 2.31	**<0.001**	6.99	1.14	4.76 – 9.22	**<0.001**
FFA Detection × Session	-1.56	1.23	-3.98 – 0.85	0.204	-3.13	2.46	-7.96 – 1.70	0.204
OFA Detection × Session	1.14	1.22	-1.25 – 3.54	0.349	2.29	2.44	-2.50 – 7.08	0.349
FFA Detection × OFA Detection × Session	0.84	0.55	-0.24 – 1.93	0.128	3.37	2.22	-0.98 – 7.72	0.128
**Random Effects**								
σ^2	255.74				255.74			
τ_{00}	135.60_{stimID}				135.60_{stimID}			
	186.90_{subID}				186.90_{subID}			
ICC	0.56				0.56			
N	22_{subID}				22_{subID}			
	50_{stimID}				50_{stimID}			
Observations	1494				1494			
Marginal R^2 / Conditional R^2	0.050 / 0.580				0.050 / 0.580			

Model 7: Recognition performance

	Model 7: Recognition (unstandardized)				Model 7: Recognition (standardized)			
Predictors	*Estimate*	*SE*	*CI*	*p*	*Estimate*	*SE*	*CI*	*p*
(Intercept)	93.23	3.48	86.40 – 100.06	**<0.001**	131.94	2.82	126.41 – 137.47	**<0.001**
FFA Recognition	-2.69	0.78	-4.22 – -1.17	**0.001**	-5.38	1.55	-8.43 – -2.33	**0.001**
OFA Recognition	-1.37	0.79	-2.92 – 0.17	0.081	-2.75	1.57	-5.83 – 0.33	0.081
Session	0.52	0.92	-1.29 – 2.33	0.571	0.52	0.92	-1.29 – 2.33	0.571
Training Day	-1.06	0.60	-2.24 – 0.12	0.080	-1.06	0.60	-2.24 – 0.12	0.080
Detection (frames)	0.45	0.02	0.40 – 0.50	**<0.001**	21.44	1.15	19.18 – 23.69	**<0.001**
FFA Recognition × OFA Recognition	-0.80	0.26	-1.31 – -0.29	**0.002**	-3.19	1.03	-5.22 – -1.16	**0.002**
FFA Recognition × Session	-3.29	1.23	-5.70 – -0.87	**0.008**	-6.57	2.47	-11.41 – -1.74	**0.008**
OFA Recognition × Session	3.00	1.26	0.53 – 5.47	**0.017**	6.00	2.52	1.06 – 10.94	**0.017**
FFA Recognition × OFA Recognition × Session	0.31	0.50	-0.66 – 1.29	0.526	1.26	1.98	-2.63 – 5.15	0.526
**Random Effects**								
σ^2	263.05				263.05			
τ_{00}	0.00_{stimID}				0.00_{stimID}			
	170.21_{subID}				170.21_{subID}			
N	22_{subID}				22_{subID}			
	50_{stimID}				50_{stimID}			
Observations	1494				1494			
Marginal R^2 / Conditional R^2	0.381 / NA				0.381 / NA			

Supplementary Figure 6. Detailed tabulation of Linear Mixed Models 6 and 7. The models assess the relationship between task-evoked FFA and OFA activity during face detection and recognition and corresponding behavioral performance (response latencies in image frames). Model 6 (left) predicts detection latencies from FFA and OFA detection responses; Model 7 (right) predicts recognition latencies from FFA and OFA recognition responses, with detection latency as an additional covariate. Both models include a three-way interaction between FFA, OFA, and training session. Unstandardized estimates are presented alongside standardized coefficients (arm::standardize) for comparison; standardized values correspond to those reported in the main text and Figure 6. Bold p-values indicate statistical significance ($p < 0.05$). Both participants (subID) and stimuli (stimID) are treated as random intercepts. EXP group only, n = 22 independent participants; 1,494 trial-level observations. Statistics were obtained from two-sided LMMs fitted with REML and Satterthwaite degrees of freedom (lme4/lmerTest). Source data are provided as a Source Data file (see Fig. 6 tab). Abbreviations: LMM, linear mixed model; FFA, fusiform face area; OFA, occipital face area; ROI, region of interest; FRMS, frames (response latency); CI, confidence interval.

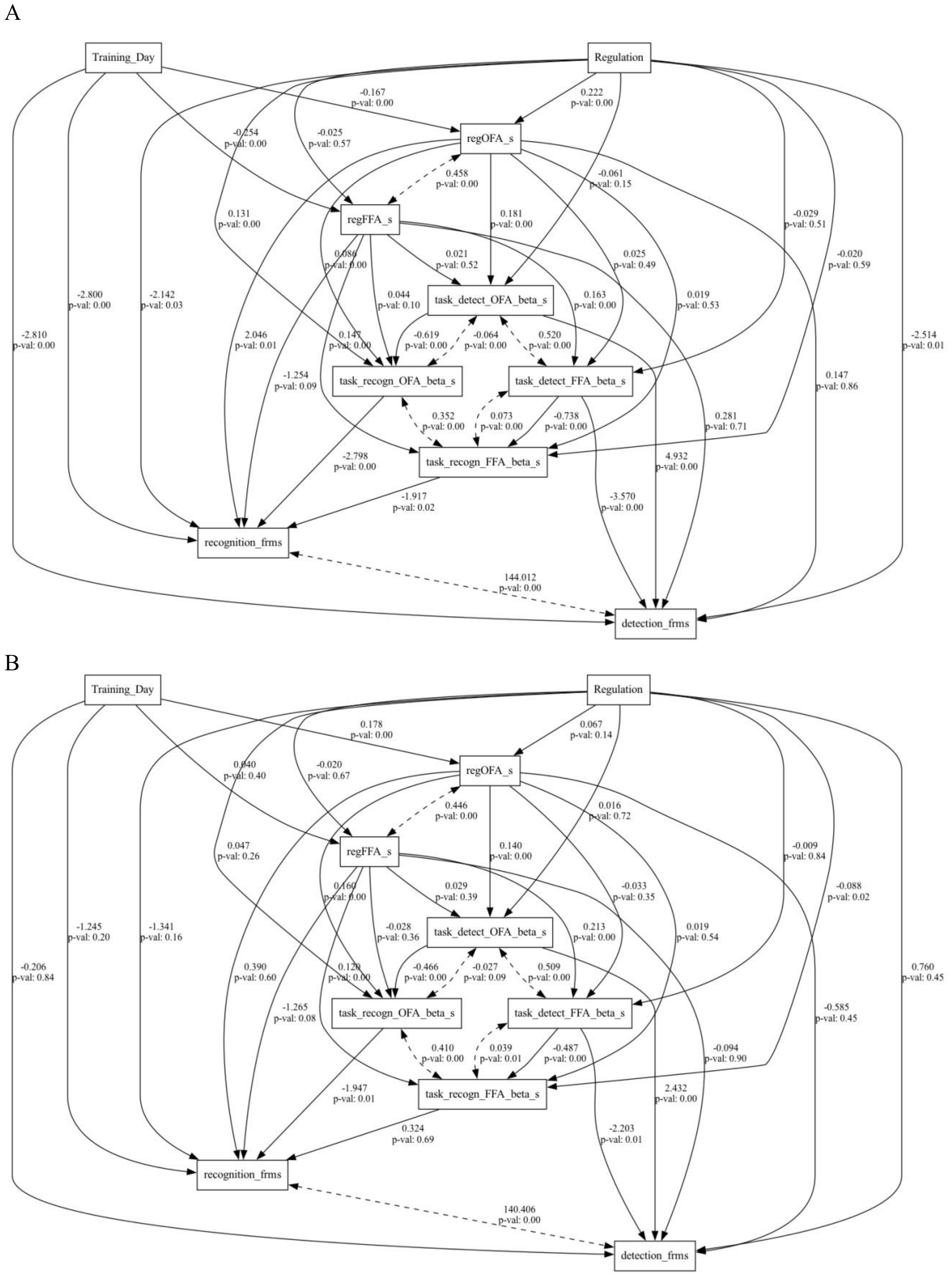

Supplementary Figure 7. Full path model output for the EXP (A) and CONT (B) groups. Path diagrams display all regression paths (solid arrows) and covariances (dashed lines) from the structural equation model described in Figure 7, including non-significant connections omitted in the main text for clarity. Each path is annotated with its unstandardized coefficient and p-value, generated directly by semopy; p-values displayed as 0.00 denote $p < 0.001$. $n = 22$ independent participants in the EXP group, $n = 20$ in the CONT group (trial-level observations). Statistical analysis: path analysis (structural equation modelling) fitted via maximum likelihood estimation (semopy, ModelMeans with subject-wise centering), two-sided. Path coefficients and p-values are estimated simultaneously within a single model. Source data are provided as a Source Data file (see Fig. 7 tab). Abbreviations: SEM, structural equation modelling; FFA, fusiform face area; OFA, occipital face area; regFFA/regOFA, self-regulation activity in FFA/OFA; NFB, neurofeedback; EXP, experimental group; CONT, control group.

ROI specificity

NFB specificity

To examine the regional specificity of our NFB manipulation, we performed additional analyses using the right STS as a control, co-activated, but non-targeted face-processing region. Time-course analyses showed that STS activation followed a similar profile across FFA and OFA sessions and was not differentially modulated during the regulation phase (Fig. 7A). LMM analyses revealed that STS showed globally higher activity during FFA sessions compared to OFA sessions, but with progressive decline over time (Fig 7B). This contrasts with the clear session-specific modulation observed in the targeted ROIs, where FFA and OFA showed distinct and sustained increases in activation during their respective training sessions (Fig. 2 main text), and with the increasing FFA/OFA dominance observed over time in model 1 (Fig. 3 main text). Together, this suggests that STS was co-activated during the task but not selectively upregulated through training.

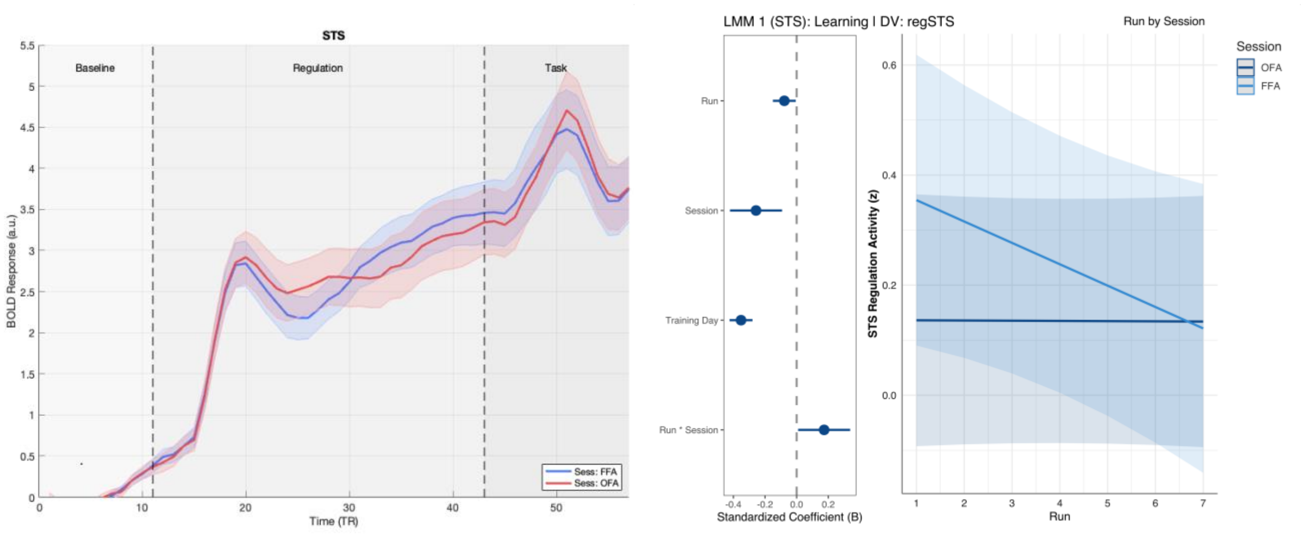

Supplementary Figure 8. STS control analysis: time-course and learning. **Left panel:** Time-course of right STS activation (mean ± SEM) across FFA and OFA training sessions, showing no selective session-specific upregulation during the regulation phase, in contrast to the targeted FFA and OFA (Fig. 2E–F, main text). Statistical analysis: volume-by-volume paired permutation test (sign-flipping on within-subject difference scores; FFA session minus OFA session), two-sided, 10,000 permutations, uncorrected for multiple comparisons across time points found not singificant differences. **Right panel:** Results of a Linear Mixed Model mirroring Model 1 (Fig. 3; main text) but with STS regulation activity as the dependent variable. STS showed globally higher activity during FFA sessions and a significant Run × Session interaction; however, this reflected a progressive decline rather than the session-specific increases observed in the targeted ROIs. EXP group only, n = 22 independent participants; 2,077 trial-level observations (all stimuli; faces and animals). Dot-whisker plots shows coefficient estimates (centre) with 95% CIs (whiskers). Shaded bands represent ±95% CI of LMM-predicted marginal effects (centre: predicted mean). Statistics were obtained from a two-sided LMM (fixed effects: Run × Session, Training Day; random effects: participant and stimulus) fitted with REML and Satterthwaite degrees of freedom. Key effects: Run × Session (ß = −0.15, 95% CI [−0.30, −0.005], $p = 0.039$), Session (ß = 0.10, 95% CI [0.03, 0.17], $p = 0.006$), Run (ß = −0.08, 95% CI [−0.15, −0.004], $p = 0.036$), Training Day (ß = −0.35, 95% CI [−0.42, −0.28], $p < 0.001$). * $p < 0.05$, ** $p < 0.01$, *** $p < 0.001$. Source data are provided as a Source Data file (see SFig. 7 and SFig. 7-TC tabs). Abbreviations: STS, superior temporal sulcus; FFA, fusiform face area; OFA, occipital face area; NFB, neurofeedback; LMM, linear mixed model; CI, confidence interval; SEM, standard error of the mean.

Behavioral Specificity

To test whether STS co-activation might (partially) account for the observed behavioral effects, we reran our behavioral models with this region (LMMs 6 & 7 main text). First, when considered alone, STS activity significantly predicted faster face detection ($\beta = -3.62$, 95% CI [−5.38, −1.85], $t(1427.63) = -4.01$, $p < .001$) and recognition ($\beta = -3.44$, 95% CI [−5.49, −1.39], $t(1484.03) = -3.30$, $p = .001$). However, when running the full model with FFA and OFA activity, STS remained a significant predictor only for detection speed ($\beta = -3.97$, 95% CI [−5.74, −2.20], $t(1424.82) = -4.39$, $p < .001$), not recognition ($\beta = 0.40$, 95% CI [−1.84, 2.65], $t(1478.82) = 0.35$, $p = .724$) (Figure 8A, 8B and 8C, next page). Critically, the key interaction between FFA and OFA we observed in LMMs 6 and 7 remained highly significant for both detection (FFA × OFA: $\beta = 6.41$, 95% CI [4.18, 8.65], $t(1418.16) = 5.63$, $p < .001$) and recognition (FFA × OFA: $\beta = -3.17$, 95% CI [−5.20, −1.14], $t(1465.17) = -3.06$, $p = .002$). So, while not ruling out contributions from other regions, these results accord with our functional characterization of FFA and OFA: *FFA activity contributes to both detection and recognition, while OFA activity is detrimental for detection but beneficial for recognition (see main text).*

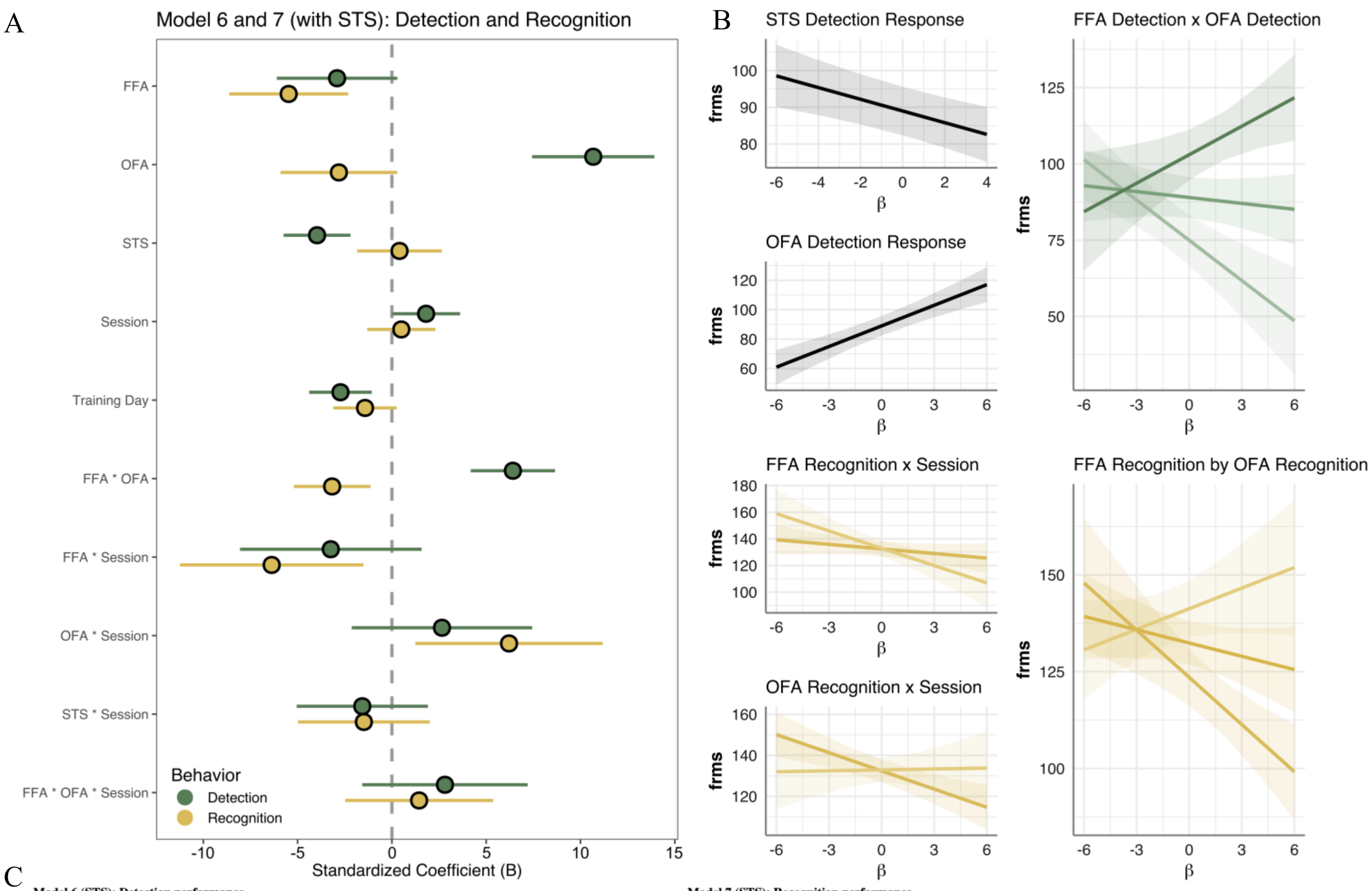

C

Model 6 (STS): Detection performance

	Model 6 (STS): Detection (unstandardized)				Model 6 (STS): Detection (standardized)			
Predictors	*Estimate*	*SE*	*CI*	*p*	*Estimate*	*SE*	*CI*	*p*
(Intercept)	88.52	3.36	81.92 – 95.12	**<0.001**	88.52	3.36	81.92 – 95.12	**<0.001**
FFA Detection	-1.45	0.81	-3.05 – 0.14	0.074	-2.91	1.63	-6.10 – 0.29	0.074
OFA Detection	5.34	0.83	3.72 – 6.96	**<0.001**	10.68	1.65	7.44 – 13.92	**<0.001**
STS Detection	-1.98	0.45	-2.87 – -1.10	**<0.001**	-3.97	0.90	-5.74 – -2.19	**<0.001**
Session	1.81	0.92	0.01 – 3.62	**0.049**	1.81	0.92	0.01 – 3.62	**0.049**
Training Day	-1.93	0.60	-3.10 – -0.76	**0.001**	-1.93	0.60	-3.10 – -0.76	**0.001**
STS Detection × Session	-0.79	0.89	-2.53 – 0.95	0.376	-1.57	1.77	-5.05 – 1.91	0.376
FFA Detection × OFA Detection	1.60	0.28	1.05 – 2.16	**<0.001**	6.42	1.14	4.18 – 8.65	**<0.001**
FFA Detection × Session	-1.62	1.23	-4.03 – 0.79	0.187	-3.24	2.45	-8.05 – 1.57	0.187
OFA Detection × Session	1.33	1.22	-1.06 – 3.72	0.277	2.65	2.44	-2.13 – 7.44	0.277
FFA Detection × OFA Detection × Session	0.70	0.56	-0.39 – 1.80	0.209	2.81	2.24	-1.57 – 7.20	0.209
**Random Effects**								
σ^2	253.12				253.12			
τ_{00}	126.21 $_{stimID}$				126.21 $_{stimID}$			
	187.52 $_{subID}$				187.52 $_{subID}$			
ICC	0.55				0.55			
N	22 $_{subID}$				22 $_{subID}$			
	50 $_{stimID}$				50 $_{stimID}$			
Observations	1494				1494			
Marginal R^2 / Conditional R^2	0.056 / 0.579				0.056 / 0.579			

Model 7 (STS): Recognition performance

	Model 7 (STS): Recognition (unstandardized)				Model 7 (STS): Recognition (standardized)			
Predictors	*Estimate*	*SE*	*CI*	*p*	*Estimate*	*SE*	*CI*	*p*
(Intercept)	93.53	3.60	86.47 – 100.59	**<0.001**	131.94	2.82	126.40 – 137.48	**<0.001**
FFA Recognition	-2.74	0.80	-4.32 – -1.16	**0.001**	-5.48	1.61	-8.63 – -2.32	**0.001**
OFA Recognition	-1.40	0.79	-2.95 – 0.14	0.075	-2.81	1.58	-5.90 – 0.29	0.075
STS Recognition	0.20	0.57	-0.92 – 1.33	0.725	0.40	1.15	-1.85 – 2.66	0.725
Session	0.50	0.92	-1.31 – 2.31	0.589	0.50	0.92	-1.31 – 2.31	0.589
Training Day	-1.01	0.61	-2.20 – 0.18	0.096	-1.01	0.61	-2.20 – 0.18	0.096
Detection (frames)	0.45	0.03	0.40 – 0.50	**<0.001**	21.28	1.25	18.82 – 23.73	**<0.001**
STS Recognition × Session	-0.74	0.89	-2.49 – 1.00	0.404	-1.49	1.78	-4.98 – 2.01	0.404
FFA Recognition × OFA Recognition	-0.79	0.26	-1.30 – -0.28	**0.002**	-3.17	1.04	-5.20 – -1.14	**0.002**
FFA Recognition × Session	-3.19	1.24	-5.62 – -0.75	**0.010**	-6.37	2.48	-11.24 – -1.51	**0.010**
OFA Recognition × Session	3.11	1.27	0.62 – 5.59	**0.014**	6.21	2.53	1.24 – 11.18	**0.014**
FFA Recognition × OFA Recognition × Session	0.36	0.50	-0.62 – 1.34	0.470	1.44	2.00	-2.48 – 5.36	0.470
**Random Effects**								
σ^2	263.25				263.25			
τ_{00}	0.00 $_{stimID}$				0.00 $_{stimID}$			
	170.66 $_{subID}$				170.66 $_{subID}$			
N	22 $_{subID}$				22 $_{subID}$			
	50 $_{stimID}$				50 $_{stimID}$			
Observations	1494				1494			
Marginal R^2 / Conditional R^2	0.382 / NA				0.382 / NA			

Supplementary Figure 9. STS control analysis: effects of FFA, OFA, and STS task-evoked activity on face detection and recognition performance (LMMs 6 & 7). **(A)** Dot-whisker plot showing standardized coefficient estimates (centre) with 95% CIs (whiskers) for both detection (dark green) and recognition (light green) models, including STS as an additional predictor alongside FFA and OFA.**(B)** LMM-predicted marginal effects (lines) with 95% CIs (shaded bands; centre: predicted mean) for selected effects: STS on detection, OFA on detection, FFA × OFA interaction on detection, FFA and OFA × Session interactions on recognition, and FFA recognition moderated by OFA recognition. **(C)** Full model tabulation of unstandardized estimates and standardized coefficients (arm::standardize, scaled by 2 SD) for Models 6 (left) and 7 (right). Standardized values correspond to those reported in the supplementary text and dot-whisker plot. Bold p-values indicate statistical significance ($p < 0.05$). Models extend the main-text Models 6 and 7 (Fig. 6) by adding right STS task-evoked activity and its interaction with training session as additional predictors. EXP group only, n = 22 independent participants;1,494 trial-level observations (face stimuli). Both participants (subID) and stimuli (stimID) are treated as random intercepts.Statistics were obtained from two-sided LMMs fitted with REML and Satterthwaite degrees of freedom (lme4/lmerTest). Source data are provided as a Source Data file (see SFig. 8 tab). Abbreviations: STS, superior temporal sulcus; FFA, fusiform face area; OFA, occipital face area; LMM, linear mixed model; CI, confidence interval; FRMS, frames (response latency); ROI, region of interest.
