# Supplementary material for: Trial-by-trial fMRI-neurofeedback dissociates fusiform and occipital contributions to face detection and recognition
Source: Nat Commun. 2026 Jun 13;17:7515. doi: 10.1038/s41467-026-74331-2 (PMC13409027; doi:10.1038/s41467-026-74331-2)
Supplement: Supplementary file 2 — Reporting Summary [file 41467_2026_74331_MOESM2_ESM.pdf]

nature portfolio

Corresponding author(s): Lucas Peek

Last updated by author(s): Apr 13, 2026

Reporting Summary

Nature Portfolio wishes to improve the reproducibility of the work that we publish. This form provides structure for consistency and transparency in reporting. For further information on Nature Portfolio policies, see our Editorial Policies and the Editorial Policy Checklist.

Statistics

For all statistical analyses, confirm that the following items are present in the figure legend, table legend, main text, or Methods section.

n/a	Confirmed	
☐	☒	The exact sample size (*n*) for each experimental group/condition, given as a discrete number and unit of measurement
☐	☒	A statement on whether measurements were taken from distinct samples or whether the same sample was measured repeatedly
☐	☒	The statistical test(s) used AND whether they are one- or two-sided<br>*Only common tests should be described solely by name; describe more complex techniques in the Methods section.*
☐	☒	A description of all covariates tested
☐	☐	A description of any assumptions or corrections, such as tests of normality and adjustment for multiple comparisons
☐	☒	A full description of the statistical parameters including central tendency (e.g. means) or other basic estimates (e.g. regression coefficient) AND variation (e.g. standard deviation) or associated estimates of uncertainty (e.g. confidence intervals)
☐	☒	For null hypothesis testing, the test statistic (e.g. *F*, *t*, *r*) with confidence intervals, effect sizes, degrees of freedom and *P* value noted<br>*Give P values as exact values whenever suitable.*
☐	☐	For Bayesian analysis, information on the choice of priors and Markov chain Monte Carlo settings
☐	☒	For hierarchical and complex designs, identification of the appropriate level for tests and full reporting of outcomes
☒	☐	Estimates of effect sizes (e.g. Cohen's *d*, Pearson's *r*), indicating how they were calculated

Our web collection on statistics for biologists contains articles on many of the points above.

Software and code

Policy information about availability of computer code

Data collection	- To prepare for the NFB sessions we developed and used the freely available prepNFB toolbox (https://github.com/lucp88/prepNFB).<br>- For real time fMRI Neurofeedback we used a Customized version of OpeNFT (http://opennft.org/)<br>- To display the feedback signal, present the task and record participant responses we used psychtoolbox (http://psychtoolbox.org/)
Data analysis	- We used custom MATLAB code to preprocess and statistically analyze the session by group time series comparisons. Parts of this process relied on adapted SPM12 functions. Visualizations were executed within the MATLAB environment.<br>- All linear mixed models were computed with R using the lmer package.<br>- Whole brain results were obtained using nipype (full workflow will be made available), final statistical results were projected on MNI templates using nilearn.<br>- For overlay mapping we relied on MRIcroGL (Rorden & Brett, 2000)<br>- Our path analyses were ran in the context of structural equation modelling. To specify and test our path model, we used the opensource software package Semopy (Igolkina & Meshcheryakov, 2020; Meshcheryakov et al., 2021)

For manuscripts utilizing custom algorithms or software that are central to the research but not yet described in published literature, software must be made available to editors and reviewers. We strongly encourage code deposition in a community repository (e.g. GitHub). See the Nature Portfolio guidelines for submitting code & software for further information.

Data

Policy information about availability of data

All manuscripts must include a data availability statement. This statement should provide the following information, where applicable:

- Accession codes, unique identifiers, or web links for publicly available datasets
- A description of any restrictions on data availability
- For clinical datasets or third party data, please ensure that the statement adheres to our policy

The source data underlying all main and supplementary figures are provided in the Source Data file published with this paper. The individual-level neuroimaging and behavioral data generated in this study are available under restricted access due to participant privacy protections under the ethical approval granted by the Ethical Committee of Geneva University Hospital (HUG), which does not permit unrestricted public deposition of identifiable neuroimaging data. Access can be obtained by contacting the corresponding author (lucas.peek@unige.ch) and completing a data use agreement specifying the intended use. Access requests will be reviewed and responded to within 30 days, subject to ethical approval. Data are available for academic, non-commercial research purposes. Data will remain available for a minimum of 10 years following publication.

Research involving human participants, their data, or biological material

Policy information about studies with human participants or human data. See also policy information about sex, gender (identity/presentation), and sexual orientation and race, ethnicity and racism.

Reporting on sex and gender	Participant sex was determined based on self-reported information, with careful consideration given to balance the distribution of sex and age within both the experimental and control groups. Given the specific aims of our research, sex was not incorporated as a covariate in our analyses. We posited no theoretical basis to anticipate differential responses to the NFB training between men and women. Gender, as a broader social construct encompassing a range of identities beyond biological sex, was not explicitly recorded or analyzed, as it did not directly pertain to the core objectives of our study.
Reporting on race, ethnicity, or other socially relevant groupings	Race, ethnicity, and other socially relevant categorizations were not documented in our study. The inclusion or exclusion of participants did not hinge upon these criteria. Our research design and participant selection process did not differentiate based on these factors, reflecting our commitment to an inclusive approach that prioritizes the examination of neurofeedback training effects across a diverse participant pool.
Population characteristics	Experimental Group: female N=14, Mean age=24, SD=3.46, range=19-34<br>Control Group: female N=9, Mean age=21.3, SD=2.93, range=18-32
Recruitment	Participants were recruited through printed advertisements disseminated across the university campus. Additionally, at the conclusion of their neurofeedback training sessions, participants were encouraged to inform their friends and classmates about the ongoing recruitment for the study, leveraging word-of-mouth to broaden our participant base.
Ethics oversight	Ethical Committee of Geneva University Hospital

Note that full information on the approval of the study protocol must also be provided in the manuscript.

Field-specific reporting

Please select the one below that is the best fit for your research. If you are not sure, read the appropriate sections before making your selection.

- [x] Life sciences
- [ ] Behavioural & social sciences
- [ ] Ecological, evolutionary & environmental sciences

For a reference copy of the document with all sections, see nature.com/documents/nr-reporting-summary-flat.pdf

Life sciences study design

All studies must disclose on these points even when the disclosure is negative.

Sample size	No sample size calculation was performed beforehand due to the novel application of our NFB training protocol, which had not been previously evaluated, leaving us without prior information to inform a precise sample size estimation. Consequently, we adopted a pragmatic approach in determining our sample size, influenced by several key factors:<br><br>Feasibility and Resource Constraints: We aimed to recruit a sample size that was both realistic and manageable within the bounds of our available resources and time frame.<br><br>Comparison to Similar Studies: In establishing our sample size, we considered the sample sizes utilized in analogous exploratory neurofeedback studies involving healthy volunteer populations. Our chosen sample size aligns with those reported in related research, which typically feature sample sizes ranging from 15 to 30 participants per group.
Data exclusions	In the experimental group 6 participants were discarded due to: excessive head motion (n=3), incomplete acquisition related to hardware issues (n=1), non-completion of the training sessions (n=1), or not being able to understand the task (n=1). From the control group, 1 participant was discarded due to excessive head motion and 1 for falling asleep during the localizer task.

Replication	No replication study was done with the same protocol in the a separate cohort. However general performance data in behavioral tasks and general fMRI results in baseline conditions as well as the task and localizer conditions replicated basic response patterns across our two groups
Randomization	In our study, participants were allocated to the experimental and control groups through a sequential method, driven by practical and methodological considerations. Initially, we focused on the experimental group to assess the effects of our novel neurofeedback (NFB) training protocol. This phase allowed us to test our experimental approach and ensure the protocol's efficacy before introducing a control condition.<br><br>The control group was matched to the experimental group in terms of age and sex to minimize the influence of these variables on the study's outcomes. Given the nature of our research and the specific hypotheses under investigation, this non-random allocation was deemed appropriate and sufficient for the study's goals. To ensure consistency in participant experience, both groups received the same scripted instructions and briefings. Furthermore, participants in both groups were coached and motivated equally before and throughout the training.
Blinding	In our study, researchers were not blinded to participant group assignments due to practical considerations and resource limitations. Ensuring the NFB system's proper functioning and monitoring for potential technical issues required researchers to be aware of whether participants were receiving actual or sham feedback. This approach allowed for immediate troubleshooting and adjustments to maintain the integrity of the NFB training sessions.<br><br>To mitigate any potential bias introduced by the lack of blinding, we employed standardized protocols across both experimental and control groups. This included using scripted instructions and briefings, as well as ensuring that all participants, regardless of their group, received consistent coaching and motivation. These standardized procedures were designed to minimize variations in the participants' experiences and responses, thereby reducing the potential influence of the researchers' awareness of group assignments on the study outcomes.<br><br>While the lack of blinding is a limitation, we believe that the measures implemented to standardize the experimental conditions and the use of objective, quantifiable outcomes in our analysis help to counterbalance this and maintain the study's scientific rigor.

Reporting for specific materials, systems and methods

We require information from authors about some types of materials, experimental systems and methods used in many studies. Here, indicate whether each material, system or method listed is relevant to your study. If you are not sure if a list item applies to your research, read the appropriate section before selecting a response.

Materials & experimental systems

n/a	Involved in the study
☒	☐ Antibodies
☒	☐ Eukaryotic cell lines
☒	☐ Palaeontology and archaeology
☒	☐ Animals and other organisms
☒	☐ Clinical data
☒	☐ Dual use research of concern
☒	☐ Plants

Methods

n/a	Involved in the study
☒	☐ ChIP-seq
☒	☐ Flow cytometry
☐	☒ MRI-based neuroimaging

Plants

Seed stocks	*Report on the source of all seed stocks or other plant material used. If applicable, state the seed stock centre and catalogue number. If plant specimens were collected from the field, describe the collection location, date and sampling procedures.*
Novel plant genotypes	*Describe the methods by which all novel plant genotypes were produced. This includes those generated by transgenic approaches, gene editing, chemical/radiation-based mutagenesis and hybridization. For transgenic lines, describe the transformation method, the number of independent lines analyzed and the generation upon which experiments were performed. For gene-edited lines, describe the editor used, the endogenous sequence targeted for editing, the targeting guide RNA sequence (if applicable) and how the editor was applied.*
Authentication	*Describe any authentication procedures for each seed stock used or novel genotype generated. Describe any experiments used to assess the effect of a mutation and, where applicable, how potential secondary effects (e.g. second site T-DNA insertions, mosiacism, off-target gene editing) were examined.*

Magnetic resonance imaging

Experimental design

Design type	Blocked design: baseline | NFB regulation | face detection and recognition trial
Design specifications	Each NFB training session consisted of 7 runs of 7 trials (49 trials in total per session). Each trial comprised 4 successive phases: baseline (20 seconds), regulation (25.6 seconds), visual task (~10 seconds), and intermittent feedback (IBF; 3.2 seconds), lasting a total of 60 seconds. To allow the beta estimation to stabilize for reliable feedback (see 2.3.2 Real-

Time fMRI Setup and Feedback Computation, page 61), the first baseline measurement of each run was 125 seconds (run 1) or 30 seconds (run 2 to 7), resulting in total run durations of ~8 minutes (run 1) or ~7min30sec (run 2-7), and a total training time of ~55 minutes.

Behavioral performance measures

In our study, we quantified participants' behavioral performance using two primary metrics: reaction times and frame numbers. To analyze these behavioral performance measures, we calculated the means and standard deviations of both reaction times and frame numbers across participants.

Acquisition

Imaging type(s)

Functional and Structural images

Field strength

3T

Sequence & imaging parameters

Functional images for all tasks (functional localizer, resting state and NFB) were acquired were acquired with a single-shot gradient-echo T2*-weighted EPI sequence (44 slices, matrix size = 64x64, voxel size = 3x3x 2.5mm3, slice gap = 0.5mm, flip angle α = 52°, bandwith 2004 Hz/Px), TR = 800ms, TE = 30 ms)

Anatomical scans. A high-resolution T1-weighted anatomical scan was acquired (3D MPRAGE, 256 × 256 × 192, voxel size = 1mm isotropic, flip angle α = 9° , bandwidth = 190 Hz/Px, TR = 1900ms, TI = 900ms, TE = 2.27ms) and used to project the real-time whole-brain statistical results on during NFB runs.

Area of acquisition

Whole brain scans. OpenNFT focussed on ROI activity (FFA and OFA). These regions were delineated based on a functional face processing task with the help of prepNFB toolbox

Diffusion MRI ☐ Used ☒ Not used

Preprocessing

Preprocessing software

Real-time fMRI processing, feedback computation and visualization was implemented within an adapted OpenNFT environment (http://opennft.org/) (Koush et al., 2017)

Offline Software. Preprocessing and statistical analyses of the anatomical and functional data
were carried out within the Nipype framework (Gorgolewski et al., 2011) using Python (version 3.8) in combination with the integrated development environment Spyder (Raybaut, 2009).

Normalization

For the anatomical scans, cortical reconstruction was performed on each participant's data using FreeSurfer's 'recon-all' process (http://surfer.nmr.mgh.harvard.edu/). ANTs 'antsRegistration' (Avants et al., 2010) function was used to obtain the transformation matrix for normalization by computing the registration between the participants' segmented brain and the MNI template.

Normalization template

fsl MNI template: fsl/data/standard/MNI152_T1_1mm_brain.nii.gz

Noise and artifact removal

Real-time time-series processing. To compute the real-time feedback, the average signal of each ROI was extracted from the smoothed whole-brain data, filtered with an autoregressive model AR(1) and a cumulative GLM with separately modelled regressors of NFB regulation and visual recognition task (convolved with hemodynamic response function), as well as head motion, linear trend and constant covariates. The constant GLM beta estimate was added back to the time-series after it was additionally filtered with a low-pass modified Kalman filter (Koush et al., 2012).

Offline Preprocessing. At the subject level, the functional data underwent several preprocessing steps, including despiking, realignment, removal of the second polynomial fit, artifact detection and smoothing. Despiking was performed using the AFNI software package (Cox, 1996), while realignment to the mean was performed using SPM12 (http://fil.ion.ucl.ac.uk/spm/). Temporal signal-to-noise ratio was calculated using the TSNR tool, with a polynomial regression of the second order used to remove low-frequency drifts. Artifact detection was performed with the RapidArt tool while the final smoothing of the images was performed using SPM12 (kernel size FWHM=8).

Volume censoring

Volume censoring in our study was addressed through despiking and artifact detection steps, ensuring data quality by removing outliers and motion artifacts. Statistical robustness was further enhanced by including covariates for detected spikes, aiding in controlling for potential confounders in our analyses.

Statistical modeling & inference

Model type and settings

For whole brain neuroimaging data, we conducted mass univariate GLM analyses at both the subject and group levels. At the subject level, individual brain responses were modeled using SPM12, with regressors of interest reflecting task and neurofeedback blocks. To address inter-subject variability and identify common patterns across participants, subject-level contrast images were taken to the second level for group analyses.

Complementing the GLM analyses, Linear Mixed Models (LMMs) were applied (using R) to assess for instance within-session trial-wise NFB learning effects, specifically examining the ability of participants to modulate differential brain activity in targeted regions of interest over the course of neurofeedback training. The LMM approach allowed us to include fixed factors such as run, session, and group, while accounting for individual differences by including participants as random effects.

Lastly, Path Analysis was utilized to explore the complex relationships between neurofeedback-induced brain activity

changes, task performance, and behavioral outcomes. This multivariate approach enabled us to construct and test theoretical models that describe how different variables influence one another within the context of neurofeedback training.

Effect(s) tested

In our NFB study, our design was used to explore the impact of NFB training on brain activity and behavioral outcomes. The NFB training conditions comprised a baseline, self-regulation (where participants were instructed to modulate their brain activity based on feedback), and task-specific conditions (i.e., faces detection and recognition). These were selected for their relevance to the targeted brain regions and the overarching goals of our NFB protocol.

Self-regulation was quantitatively defined by the differential percent signal change (PSC) values between FFA and OFA, aiming to capture the extent of participants' ability to modulate their brain activity using real-time feedback. Task conditions were operationalized through the presentation of visual stimuli (faces and animals) designed to evoke natural responses in the same ROIs. This setup facilitated a direct comparison between the effects of voluntary control (via NFB) and stimulus-evoked brain responses.

For our statistical analysis framework, we implemented both within-subject (paired) and between-group (experimental vs. control) comparisons to delineate the effects of NFB training. This methodological choice enabled us to dissect the specific impact of NFB on brain dynamics and behavioral performance, distinguishing these from changes attributable to time or repeated task engagement.

Although our design involved comparisons that are characteristic of factorial designs, our primary analysis focused on the use of GLMs and Linear Mixed Models (LMMs) rather than traditional ANOVAs. This choice was informed by the hierarchical structure of our data, encompassing trial-by-trial observations across participants rather than session averages.

Specify type of analysis: ☐ Whole brain ☐ ROI-based ☒ Both

Anatomical location(s)

In our study, functional locations for regions of interest (ROIs) were determined using a data-driven approach rather than relying on automated labeling algorithms or probabilistic atlases. Specifically, we employed a task-based localizer sequence to identify areas responsive to specific stimulus categories, enabling the functional delineation of ROIs such as the Fusiform Face Area (FFA) and the Occipital Face Area (OFA). The prepNFB toolbox was used during delineation and creation of the image files.

Statistic type for inference

(See Eklund et al. 2016)

Voxel-wise

Correction

FWE

Models & analysis

n/a	Involved in the study
☒	☐ Functional and/or effective connectivity
☒	☐ Graph analysis
☒	☐ Multivariate modeling or predictive analysis
