# Supplementary material for: Trial-by-trial fMRI-neurofeedback dissociates fusiform and occipital contributions to face detection and recognition
Source: Nat Commun. 2026 Jun 13;17:7515. doi: 10.1038/s41467-026-74331-2 (PMC13409027; doi:10.1038/s41467-026-74331-2)
Supplement: Supplementary file 3 — Transparent Peer Review file [file 41467_2026_74331_MOESM3_ESM.pdf]

nature portfolio

Peer Review File

Trial-by-trial neurofeedback dissociates fusiform and occipital contributions to face detection and recognition

Corresponding Author: Dr Lucas Peek

This file contains all reviewer reports in order by version, followed by all author rebuttals in order by version.

Version 0:

Reviewer comments:

Reviewer #1

(Remarks to the Author)
This study using real-time fMRI shows two potentially interesting findings. First, individual participants can self-regulate neural activity in two different category-selective regions, i.e., face-selective areas of their right hemisphere. Second, the self-regulation modulates performance in a different way for the two areas (OFA and FFA), a result that is interested in favour of a non-hierarchical model of human face perception.

General evaluation and main comments: The study is based on a large amount of work and appear to have been carefully executedin terms of data recording and analysis. Even if the authors have attempted as much as possible to simplify the presentation of the results, the paper is long and difficult to read, with a relatively complex methodology. The introduction is relatively clear but could be more concise, particularly in the presentation of the hierarchical and non-hierarchical hypotheses.

- There is an over-reliance on statistical modelling, with no actual data shown (expect for some example BOLD time-courses in Figure 2). All other figures only display model parameters and not the actual data. This is not a standard good
practice for empirical/experimental work.

- Although the method itself is interesting and novel in the context of face processing, the study does not bring stronger or or decisive evidence regarding the debate between hierarchical and non-hierarchical models of face processing: isn't evidence of FFA activity despite OFA lesion, as presented in the paper (also in Weiner et al., 2016; https://doi.org/10.1523/JNEUROSCI.4509-15.2016), clearer already that the hierarchical model isn't correct than the evidence provided in the present paper?

- In general, how can we be sure that if a given region is involved in a task, its level of activation will be correlated with an increase in performance at the task? Evidence that a region is activated during a given task doesn't necessarily imply that an activation of the region (outside of the task, i.e. during NFB training) will lead to better performance in the task. In other words, the neurons or the neurophysiological processes that are involved in a region during a certain task may be different from the neurophysiological processes that are called upon in the same region during NFB training.

- How much the effects observed during the task phase might be driven by signal carried over from the Regulation phase with
no real modulation due to the task itself (except maybe the OFA negative effect on detection). Figure 2 would suggest that. Maybe that is what the authors are after, but then why provide a separate analyses for the amplitude of the signal in both regions during the task phase
separately from the regulation phase?

- How do the authors interpret the (almost) lack of training effect on the FFA. It seems that given this absence of NFB training effect on the FFA, any relationship between FFA activity and behaviour is difficult to relate to FFA NFB training (see e.g. Lines 716- 717). Along the same lines, authors use the term (self)regulation effects of FFA and OFA for variations of BOLD signal in these regions during the regulation phase. However, it seems that at least some of these variations of BOLD signal

may be random variations and/or not related to the regulation process per se (especially for FFA).

- In the section ‘Impact of Self-regulation on Subsequent Activation of Target ROIs During Face Detection and Recognition ‘, the authors do not provide analyses for the CONT group. However, it would still be interesting to see what happens in the CONT group to have a better knowledge of the specificity of the effects, e.g. to know if the magnitude of the OFA or FFA during regulation affects the magnitude of the response in these regions during tasks in the same manner as for the EXP group. The same is true for the influence of the signal during regulation on the behavioural performance. Is it specific to the EXP group?

In the view of this reviewer, the main novel finding of the study is the demonstration of a (differential) self-regulation of activity in the OFA and FFA with rt-fMRI. This is novel but does not have theoretical implications, and given previous evidence of self-regulation neural activity in fMRI in high-level visual cortex, does not represent substantial novel evidence

Minor:

- Figure 2, no units are mentioned or indicated for the x (time) and y (BOLD signal % signal change ?) axis.

- Figure 5: The panels would be easier to read and interpret if the scales matched, at least within region x tasks.

- Too many different terms are used to refer to the same functions: face detection/face categorization; face recognition/face identity discrimination/identity recognition … this shul be homogenous, for aske of clarity.

- Did Gauthier et al., 2000 propose that the OFA mediates face detection (p.3)? It seems that that study instead proposed that BOTH regions are involved in face identity discrimination (based on adaptation).

- Tsao & Livingstone (2008; e.g., p.34) suggested that there must be a face detection stage preceding a ‘measurement’ stage in face processing, but certainly did not argue in favor of that stage being at the level of the FFA (vs. the OFA), in a non-hierarchical organization.

Reviewer #2

(Remarks to the Author)
The study investigates the possibility and the impact of modulating activity in two core areas of the face processing network (FFA, OFA) via NFB. The authors provide evidence that participants are able to modulate the difference in activation between the two areas. Further, this difference impacts activation in the same areas during subsequent detection and recognition tasks as well as the reaction time associated with these tasks. Theoretically, the authors argue that the results support a non-hierarchical model of face processing that relies on the FFA as an entry point and a key area for face detection. Overall, the study makes a significant contribution to the field: the ability to modulate activity in specific nodes of the face network via NFB provides a novel and informative tool for understanding its functional architecture. The manuscript is clearly written and I enjoyed reading it. My comments below aim primarily to enhance clarity and to strengthen the conclusions of the study.

1. Activity in the FFA appears robust in the regulation stage even in the control group across both sessions (Fig 2A, G). I wonder what accounts for this: is it an anticipatory response associated with the subsequent face detection task? Also, the difference between the experimental and the control group in the same figure is not easy to interpret since the markers are not labeled with a significance value, there is no mention of multiple-comparison correction, the shaded areas are not labeled as SE/CI etc.
2. Participants were advised to explore face-related visualization strategies to modulate activity. It would be informative to know, even anecdotally, what strategies were found successful in upregulating FFA vs OFA activity.
3. In the presence of high OFA activity, it seems that detection performance is improved if FFA activity is reduced (Fig. 6c). Could this indicate an alternative part-based strategy for face detection, supported by the OFA, but interfering with a more holistic strategy deployed by the FFA?
4. The NFB session has a direct advantageous effect on detection and recognition performance in the experimental group (Fig. 7b). Somewhat surprisingly though, there is a “benefit in the OFA training session, independent of task-evoked activity” (lines 714-716), running counter to the impact of higher OFA activity during detection. One possibility relies on the contribution of additional regions recruited during regulation. Alternatively, though, I wonder if this does not simply reflect a higher level of motivation during the detection/recognition tasks associated with the successful use of strategies in the experimental (versus control) group. Since the authors collected data on the level of motivation of the participants for each run, this possibility can be easily addressed.
5. The authors state that “regulating FFA did not change the detection responses in OFA” but Fig. 5b seems to indicate otherwise. Please clarify.

Smaller points:
- Were trials with incorrect responses removed? How many?
- The authors refer to a Fig. 8 (line 765), which does not appear in the manuscript

A. Nestor

Reviewer #3

(Remarks to the Author)
The authors aimed at dissecting the specific functional role of OFA and FFA in relation to face processing by differentially manipulating the BOLD activity in these two regions using rtfMRI neurofeedback, and by concurrently testing how learned differential regulation of OFA and FFA activity affects consecutive face detection/recognition tasks.

The article is well structured and written in all sections.
The objective of the study, whose rational is appropriately introduced and motivated, is neuroscientifically challenging and potentially quite influential.
The methodology is clear and sufficiently detailed, however it does not appear particularly innovative and advanced. For instance, a differential feedback between two cortical areas (SMA vs PPA) was already proposed by Weiskopf et al. 2004. In addition, the assessment of the behavioral effects of regulation on a trial-by-trial basis was also previously proposed by Caria et al.2010 where emotional stimulus perception and evaluation was interleaved with consecutive regulation blocks.

More importantly, there are some methodological issues that critically impact the results and their interpretation.

My major objection is that a differential BOLD activation (dominance) in FFA wrt OFA, or viceversa, might be sufficient to clearly dissect the distinct functional role of these regions in face processing. Specific engagement of visual areas during a visual task is not necessarily reflected by an overall increased/decreased ROI activation. For instance Shibata et al 2011 showed that increased visual stimulus detection was associated with a learned control of stimulus-specific brain pattern in V1/V2 resulting in a combination of positive and negative voxels almost symmetrically distributed around 0. Similarly, as FFA and OFA are posited to process face-specific local or global features, highly selective BOLD activity patterns might indeed characterize OFA and FFA activity. This features selective activity, that in fact might be modulated during self-regulation, would then result in either increased, decreased or null mean BOLD activity. However, the interpretation of the potential influence on consecutive face detection/recognition tasks would be then confounded when discussed in terms of a higher or lower activation and not in terms of the actual face-related representational content, that might be highly specific also in case of an overall low or null BOLD activation.

In addition, mean BOLD signal magnitude can be also modulated by confounding non neural factors such as changes of basal vascular physiology, that are region specific (Kurzawski et la. 2022, Williams et al. 2023). Such a potential variance, that during neurofeedback would play a critical role, is not sufficiently controlled/reduced by a differential feedback between two regions, as these regions might be characterized by different physiological mechanisms.

A second major issue is related to the assumption that the preceding activity affects consecutive face stimuli presentation. Considering the low temporal resolution of the BOLD signal and of the experimental protocol the persistence of the preceding modulation of FFA or OFA dominance activity during face processing is not certain, and difficult to prove in the presence of a visual stimulus. In fact, in Figure 2 the increased BOLD activity extending into the task phase appears to reflect an initial effect of previous regulation block and its associated delayed response (about 5 to 6s), and a later increase likely because of the face stimulus presentation.
In any case, it would be important to propose a clear explanation of the hypothesized sustained effect in terms of possible long lasting (reverberating?) neuronal processes elicited by neurofeedback-based regulation.

Furthermore, as increased neural activity is inevitably associated with explicit or implicit perception/behavior a more clear understanding of how mental strategies during regulation affects self-regulation itself as well as the consecutive face detection/recognition tasks is highly desirable (for instance by analyzing more clearly what specific strategies were adopted by the participants), so as to prevent readers to think that brain and mind do work independently, in particular during self-regulation.
This would also be possibly informative of the observed differential ability in modulating FFA and OFA dominance.

Finally, the authors did not test whether the additional brain regions involved in self-regulation (paragraph 3.1.3) also explain (predict) the observed changes in face detection/recognition. Such a control is important to demonstrate the specificity of the ROIs-induced effects.

Additional points

page 17 lines 464/472 Description of LLMs for detection and recognition should be improved as it is currently not clear. The authors refer to recognition models but not to detection models. Lines 471/472 p 17. as the models mentioned in the paragraph ”Effects of Self-Regulation Performance on ROI Detection and Recognition Responses” are 4 what are the numbers 3 and 5 referring to? I suppose that model 1 refers to NFB learning mentioned in the previous paragraph whereas models 2 to 5 refer to those mentioned in the current paragraph, but that’s not evident.

page 17 lines 474/476, the author state that “a positive association between regFFA and FFA detection activity would suggest that the FFA during self-regulation predicts subsequent enhanced FFA detection activity.” This is a rather speculative assumption as a positive association might be also result from increased activity associated with face-related imagery during regulation and face processing during face detection task, without implying any predictive/causal dependency.

Version 1:

Reviewer comments:

Reviewer #2

(Remarks to the Author)
The authors have addressed all concerns.

Open Access This Peer Review File is licensed under a Creative Commons Attribution 4.0 International License, which permits use, sharing, adaptation, distribution and reproduction in any medium or format, as long as you give appropriate credit to the original author(s) and the source, provide a link to the Creative Commons license, and indicate if changes were made.
In cases where reviewers are anonymous, credit should be given to 'Anonymous Referee' and the source.
The images or other third party material in this Peer Review File are included in the article’s Creative Commons license, unless indicated otherwise in a credit line to the material. If material is not included in the article’s Creative Commons license and your intended use is not permitted by statutory regulation or exceeds the permitted use, you will need to obtain permission directly from the copyright holder.
To view a copy of this license, visit https://creativecommons.org/licenses/by/4.0/

Reviewer 1

Global comment of Reviewer 1:
"*This study using real-time fMRI shows two potentially interesting findings. First, individual participants can self-regulate neural activity in two different category-selective regions, i.e., face-selective areas of their right hemisphere. Second, the self-regulation modulates performance in a different way for the two areas (OFA and FFA), a result that is interested in favour of a non-hierarchical model of human face perception.*
General evaluation and main comments: The study is based on a large amount of work and appear to have been carefully executed in terms of data recording and analysis. Even if the authors have attempted as much as possible to simplify the presentation of the results, the paper is long and difficult to read, with a relatively complex methodology. The introduction is relatively clear but could be more concise, particularly in the presentation of the hierarchical and non-hierarchical hypotheses."

Response:
We thank the reviewer for these positive and encouraging comments. Below we addressed all their comments and feel they helped us clarify the paper and eased reading.

Major

Reviewer 1, comment #1:
"There is an over-reliance on statistical modelling, with no actual data shown (except in figure 2). All other figures only display model parameters and not the actual data. This is not a standard good practice for empirical/experimental work."

Response:
We agree that visualizing actual data is a valuable practice in empirical research, as it allows readers to assess the distribution, variability, and robustness of the findings. However, modelling is key to ensure valid statistical inferences and reliable conclusions.

In our study, we employed linear mixed models (LMMs) to ease the presentation and interpretation of results specifically when they were based on hierarchical data consisting of multiple nested levels—i.e., trials within runs, runs within sessions, and sessions within subjects. LMMs are particularly well-suited for such data structures because they account for both fixed effects (e.g., experimental conditions) and random effects (e.g., individual differences among participants). This approach enables us to model the inherent variability at each level and make accurate inferences about the effects of interest. Moreover, statistical modelling provides the standard criteria to determine the significance / reliability of experimental effects, hence these results constitute a concise and essential description of the findings. Please also note that modelling (e.g. GLM) is a standard approach to report fMRI results, although the latter are usually limited to simpler experimental conditions such as linear contrasts between non-hierarchical conditions.

We believe these points justify our general approach. Given the complexity and high dimensionality of our dataset, visualizing raw data points and relating them to factors of interest in a way that is both meaningful and interpretable would present several challenges; most notably:

- **Overplotting and clutter:** Plotting individual data points for all trials and participants across conditions would result in an overwhelming number of data points, potentially obscuring patterns and making it difficult for readers to discern meaningful trends.

- **Misrepresentation of effects:** Raw data plots may not accurately reflect the effects captured by the LMMs, especially when there are multiple sources of variability that the models account for but are not easily visualized in simple plots.

These are the reasons why neurofeedback effects in our study are reported using LMMs. However, we do understand the importance of transparency and the value of displaying actual data to illustrate findings. To address the reviewer's concern and improve our manuscript, we therefore propose to add to the supplementary materials the following for our first model (LMM 1; figure 3 in manuscript):

Raw Data (Suppl. Mat. Fig. 1): Mean trajectories with data distribution per run (A), individual subject trajectories across runs (B) and individual learning slopes for each regulation session (C).

Model fit plots (Suppl. Mat. Fig. 1): We now include diagnostic plots for the LMM 1 (as an example), such as residuals versus fitted (D) and observed versus fitted values (E) to demonstrate the adequacy of the models and to illustrate how well the models capture the data patterns.

Reference to the figure can be found on **page 24** in the manuscript.

We are grateful to the Reviewer's comment which prompted us to improve our presentation of the results. Of course, we would be happy to also provide additional plots with data clouds in Supplementary Material if the Reviewer and Editor find it crucial.

Reviewer 1, comment #2:
"Although the method itself is interesting and novel in the context of face processing, the study does not bring stronger or decisive evidence regarding the debate between hierarchical and non-hierarchical models of face processing: isn't evidence of FFA activity despite OFA lesion, as presented in the paper (also in Weiner et al., 2016; https://doi.org/10.1523/JNEUROSCI.4509-15.2016), clearer already that the hierarchical model isn't correct than the evidence provided in the present paper?"

Response:
We acknowledge that the evidence from Weiner et al. (2016) did challenge the traditional hierarchical model by demonstrating preserved FFA activity despite OFA lesions. It is also cited in our paper. However, this observation pertains to the flow of face information more than the nature of information processing. Our study contributes additional and complementary insights into the functional interplay between the FFA and OFA, specifically addressing the relative contributions of each region to perceptual function and the potential mechanisms driving these effects.

More specifically:

- **Weiner et al. (2016)** report convincing evidence against a strict hierarchical model by showing that the FFA can remain active even when the OFA is lesioned. This suggests that the FFA can receive inputs from regions other than the OFA, thus challenging the notion of a simple feedforward pathway from OFA to FFA.

- While the latter observation highlights the independence of FFA activity from OFA, it does not provide detailed insights into the relative contributions of these regions to face processing tasks. Preserved FFA activity could potentially be driven by inputs from other regions, possibly linked to the broader, bilateral, and complex network of face processing. However, this does not necessarily imply that the FFA can fully compensate for the absence of OFA input in all aspects of face processing. Without OFA, the quality and nature of the input to the FFA might differ, potentially affecting the FFA's ability to represent faces accurately.

- **Our study** addresses this gap through a more direct/causal test of their mechanistic role in face processing. By using a controlled manipulation of their activity with neurofeedback, we were able to causally and selectively enhance the recruitment of either the FFA or OFA and then measure the subsequent effects on face detection and recognition tasks. This allows us to delineate the specific functional roles of each region in face processing. These findings support a model where both regions contribute uniquely to different stages of face processing, suggesting that while the FFA can function without direct OFA input, the OFA still plays a critical role in refining the information processed by the FFA.

In sum, while we agree that the two studies converge to offer evidence in favour of a similar conclusion, we feel our study goes one step further conceptually, both in terms of methodology and functional insights, in way that are theoretically important and unprecedented. We have included a reference to Weiner et al 2016 in our manuscript **(p4)**.

Reviewer 1, comment #3
"In general, how can we be sure that if a given region is involved in a task, its level of activation will be correlated with an increase in performance at the task? Evidence that a region is activated during a given task doesn't necessarily imply that an activation of the region (outside of the task, i.e. during NFB training) will lead to better performance in the task. In other words, the neurons or the neurophysiological processes that are involved in a region during a certain task may be different from the neurophysiological processes that are called upon in the same region during NFB training."

Response:
Thank you for this thoughtful point. We fully agree that activation of a brain region during a task does not inherently guarantee that artificially increasing its activation via NFB should always lead to improved task performance. However, both theoretical frameworks and empirical evidence support the notion that targeted modulation of specific brain regions (through NFB or any other technique) is generally associated with enhancement of the cognitive function related to these regions.

Firstly, several studies have demonstrated a positive correlation between the level of activation in face-processing regions and performance on face perception tasks. For instance, higher stimulus-evoked activity in the FFA has been associated with better face recognition performance (Elbich & Scherf, 2017; Grill-Spector et al., 2004; Weibert & Andrews, 2015). Similarly, increased pre-stimulus activity in the OFA and FFA has been linked to improved face detection and recognition (Esterman & Yantis, 2010; Hesselmann et al., 2008; Peatfield et al., 2017). These findings suggest that modulating activity in these regions could potentially influence task performance.

Moreover, empirical evidence from NFB studies indicates that training individuals to self-regulate specific brain regions can lead to behavioral improvements in tasks associated with those regions (deCharms et al., 2005; Robineau et al., 2014; Scharnowski et al., 2012). These studies suggest that the neurophysiological processes engaged during NFB training overlap with those activated during task performance, leading to functional improvements. The modulation achieved through NFB likely recruits similar neuronal populations and pathways as those involved in natural task-related activation. It is generally assumed that intrinsic connectivity and neuronal responsivity among these populations are strengthened subsequent to training.

In our study, participants who successfully upregulated activity in the FFA or OFA via NFB demonstrated corresponding changes in face detection and identity recognition tasks. Specifically, enhanced FFA activity led to faster face detection, while enhanced OFA activity contributed to optimal identity recognition when both regions were upregulated. This indicates that the activation induced by NFB training did engage relevant neural mechanisms contributing to task performance.

Please also note that our results could have been different and falsify this hypothesis; but the pattern of findings did turn out to accord with these assumptions – fully consistent with typical findings in NFB research. In addition, the question of whether higher BOLD signal in fMRI necessarily implies better processing is a more general issue that goes beyond the realm of our work and likely varies depending on task or domains. Again, the current results turned out to indicate that higher BOLD responses during our face processing tasks predicted increases in performance, but it could have been otherwise.

More generally, our findings align with theories of experience-dependent neuroplasticity, where targeted modulation of brain activity can enhance cognitive functions associated with the regulated regions (Ros et al., 2014). We therefore find our results compelling enough to suggest that by engaging participants in *task-relevant* upregulation of specific face-processing areas, we facilitated neural changes that translated into measurable behavioral improvements. We briefly comment on this point in our revision to recognize its importance **(p5)**.

Reviewer 1, comment #4

"How much the effects observed during the task phase might be driven by signal carried over from the Regulation phase with no real modulation due to the task itself (except maybe the OFA negative effect on detection). Figure 2 would suggest that. Maybe that is what the authors are after, but then why provide a separate analysis for the amplitude of the signal in both regions during the task phase separately from the regulation phase?"

Response:

This is again a relevant point worth clarifying. It is true that higher neural activity induced during the regulation phase could persist into the task phase; but critically this might have different causes, i.e., either due to a functional modulation of the trained neural pathways or more trivially due to the sluggish nature of the BOLD response.

To some extent, a functional carryover effect was an intentional aspect of our experimental design. Our primary objective was to use pre-trial neurofeedback (NFB) to boost brain activity in the target regions (FFA and OFA) immediately before the task, with the goal of influencing subsequent perceptual abilities in face detection and recognition. By interleaving regulation and task phases, we aimed to create a direct link between the self-regulation of neural activity and

task performance. Based on the reasoning below we believe that the task effects were at least partially phase specific rather than a simple carry-over of (e.g. vascularly driven) BOLD signal.

Firstly, we analysed the task phase separately from the regulation phase to explore the direct functional relationships between regional brain activity and behavioural performance. Specifically, we wanted to examine how activation in the FFA and OFA during the task phase relates to face detection and recognition performance. This analysis helped us understand the role of these regions during the task itself (through the intended 'boosting' of these functions). Such relationship might in principle exist independently of any prior modulation. Hence, this analysis helped us clarify whether and how higher OFA/FFA activation to face stimuli related to detection/recognition performance, which seems essential to back up our interpretations.

The reviewer's comment also touches upon an interesting point regarding whether we can claim that task-evoked activations contribute uniquely to performance outcomes as, by themselves, these analyses do not consider regulation-induced effects on activation. Therefore, to address this concern, we conducted a comprehensive path analysis that included both regulation-induced activation and task-evoked activation within a single analytical framework. This approach allowed us to model the contributions of both phases simultaneously and to control for the influence of regulation-induced activation when assessing the effects of task-evoked activation on behaviour. Results from this analysis clearly show that task-evoked activations in the ROIs had distinct effects on behavioural performance, even after accounting for the preceding regulation-induced activation. This suggests that, even if non-specific activity carryover effects might have been present, the task itself also elicited additional neural responses in the target regions that independently influenced performance.

On the other hand, a trivial (e.g. vascular) sluggishness of BOLD would not account for the task-related and regulation-related differences in OFA and FFA activity, nor for differences in behavioral performance. Exactly as noted by the Reviewer themselves, this could not explain why increases in OFA would impair detection while it simultaneously improves recognition.

For all these reasons, we feel that NFB induced a meaningful functional carry over in regional activity and recruitment that go beyond simple BOLD inertia. We added brief comments in the revision to clarify this important point **(p19, p42)**.

Reviewer 1, comment #5

"How do the authors interpret the (almost) lack of training effect on the FFA. It seems that given this absence of NFB training effect on the FFA, any relationship between FFA activity and behaviour is difficult to relate to FFA NFB training (see e.g. Lines 716- 717). Along the same lines, authors use the term (self)regulation effects of FFA and OFA for variations of BOLD signal in these regions during the regulation phase. However, it seems that at least some of these variations of BOLD signal may be random variations and/or not related to the regulation process per se (especially for FFA)."

Response:

Thank you for raising this point and prompting us to clarify the paper. We respectfully disagree with this statement which does not fully reflect the results. Although it is true that, on average, the FFA increase seen during NFB training was less pronounced compared to the OFA increases during OFA training, there was other evidence of reliable NFB effects. First of all, relative to the baseline prior to regulation, FFA was significantly enhanced during regulation in the active NFB group – but not in the sham group. Second, and more importantly, there was a progressive increase in FFA activity over

successive regulation runs with concomitant decreases in OFA during FFA regulation – but the opposite pattern during OFA regulation. Third, our path analysis with SEM demonstrated significant relationships between regulation session (FFA vs OFA) and behavioural face processing performance (detection vs recognition), which again did not exist in the sham group. Taken together, these findings suggest meaningful effects of NFB regulation on FFA and associated behavioural outcomes (**Sections 4.2, 4.4, and 4.5**).

Moreover, what the reviewer seems to refer to here is the lack of differential increases in FFA relative to OFA in the experimental group when activity is averaged across all runs (i.e. including the very first training blocks where NFB success is unlikely), which contrast with larger and more rapid training effects in OFA. In our revised paper, we do offer an interpretation for this attenuated regulation effect in FFA, as now detailed in section 4.2 **(p37)**.

To summarize, the FFA is known to be critically involved in face processing and often exhibits high baseline activation during face perception tasks independent of stimulus-evoked responses (Tsao & Livingstone, 2008). FFA activity is also increased by mere face expectation in the absence of actual face stimuli (Righart et al., Cereb Cortex 2010). This inherent high activity may have occurred due to face processing demands in our paradigm and thus resulted in a ceiling effect, making additional upregulation through NFB more challenging to detect at the group level. In fact, this would be consistent with the view that FFA provides an "obligatory" entry point into face processing and therefore generally activates due to task demands. We do acknowledge however that other interpretations cannot be ruled out, or that FFA regulation might be more difficult than OFA regulation and thus the effect of NFB less prominent. Accordingly, we revised the manuscript to address this point by considering these alternative explanations in more details, and noting that regulation effects in FFA could have been more convincingly demonstrated if there had been a main effect like seen in OFA **(p37).**

Reviewer 1, comment #6

"In the section 'Impact of Self-regulation on Subsequent Activation of Target ROIs During Face Detection and Recognition', the authors do not provide analyses for the CONT group. However, it would still be interesting to see what happens in the CONT group to have a better knowledge of the specificity of the effects, e.g. to know if the magnitude of the OFA or FFA during regulation affects the magnitude of the response in these regions during tasks in the same manner as for the EXP group. The same is true for the influence of the signal during regulation on the behavioural performance. Is it specific to the EXP group?"

Response:

While we omitted these results in the manuscript for brevity, we agree that presenting these analyses can offer additional clarity. However, please note these data were actually described in our path analysis with SEM. Briefly, our results indicate that the CONT group corroborates the observation in the EXP group regarding the relationship between regulation-phase activity (regROI) and task-phase activity (taskROI) in the FFA and OFA. However, critically, there was no main or interaction effects with session observed in the CONT group. This absence of session-dependent effects in CONT supports the specificity of NFB training in the EXP group as the driver of these changes.

Our choice to restrict the reported analyses to the EXP group was made for two reasons: First *to focus on NFB training effects*: Given the lack of NFB modulation in the CONT group, our primary

interest was to evaluate how NFB training (effective in the EXP group) influenced the relationship between regulation and task-phase activity, as well as the associated behavioral outcomes. There is no reason to conduct the same follow-up analysis in CONT. Second, *to ensure manuscript brevity and conciseness*: Including the same detailed analyses for the CONT group would overcomplicate the narrative without substantially altering the conclusions about NFB-specific effects. We now made sure to clearly justify this choice in the manuscript (p39).

Reviewer 1, comment #7

"In the view of this reviewer, the main novel finding of the study is the demonstration of a (differential) self-regulation of activity in the OFA and FFA with rt-fMRI. This is novel but does not have theoretical implications, and given previous evidence of self-regulation neural activity in fMRI in high-level visual cortex, does not represent substantial novel evidence."

Response:

We feel this statement is only partly correct and unfairly trivialize our work. While the real-time fMRI NFB technique has been demonstrated in prior research, we would like to emphasize the unique contributions of our study, which extend beyond individual points to the combination of factors:

First, our study is the first to obtain a differential modulation of two functionally linked regions that operate within the same functional network and mediate the same general cognitive / perceptual ability (i.e. face processing). While previous studies have established self-regulation of single regions or unrelated pairs of regions, our study is among the first to target multiple, interconnected regions involved in the same cognitive process, dissecting their complementary roles.

Second, our study is novel by using NFB to test for a quasi-causal link to behavioural changes on a trial-by-trial basis, rather than to obtain more sustained training benefits (e.g. rehabilitation). Specifically, we were able to show that a differential modulation of FFA and OFA activity predicted dissociable enhancements in face detection and recognition performance, respectively, bridging the gap between neural regulation and its functional impact. Further, we believe that our trial-by-trial basis approach is particularly suited to our purpose of linking dynamic neural modulation to immediate behavioural outcomes, allowing us to capture fine-grained relationships between regional activity and performance.

Third, our use of NFB has important theoretical implications for face processing models. While we do agree (and acknowledge) that our results are neither the first nor the only ones to suggest non-hierarchical functioning of OFA-FFA, we believe the ability to differentially modulate distinct nodes in the same functional network and observe distinct behavioral changes is a novel and important contribution to our understanding of network-level description of face processing in the human brain. More generally, this methodological framework lays the groundwork for future research exploring more complex neural systems using similar approaches.

Minor

Reviewer 1, comment #1

“Did Gauthier et al., 2000 propose that the OFA mediates face detection (p.3)? It seems that that study instead proposed that BOTH regions are involved in face identity discrimination (based on adaptation).”

Response:

We agree that Gauthier et al. (2000) primarily demonstrated that the OFA is sensitive to face identity, as shown through adaptation effects, and therefore suggested its function is therefore not restricted to extracting information for face detection. However, Gauthier et al. also left open the possibility that the location of the OFA in the early stages of the face processing hierarchy makes it a plausible candidate for a putative detection stage. Importantly, other influential studies (e.g., Dubois et al., 1999; Haxby et al., 1999) have explicitly suggested that the OFA may play a role in detecting faces at the basic perceptual level. This broader body of evidence provides the foundation for considering the OFA as relevant to face detection while acknowledging its more established role in face identity discrimination. We have rephrased the relevant text in the manuscript **(p3).**

Reviewer 1, comment #2

“Tsao & Livingstone (2008; e.g., p.34) suggested that there must be a face detection stage preceding a ‘measurement’ stage in face processing, but certainly did not argue in favor of that stage being at the level of the FFA (vs. the OFA), in a non-hierarchical organization.”

Response:

Thank you for highlighting this point. In fact, there is some ambiguity in how Tsao & Livingstone distinguish the putative functions of the FFA and OFA. However, they emphasize the FFA’s role in face-versus-object classification, while they consider that OFA might be more sensitive to processing specific features or identity-related cues. This interpretation suggests complementary roles within the face processing network, but indeed does not assign detection exclusively to one region. We have rephrased our citation of their work accordingly **(p38).**

We are grateful to this reviewer for their insightful comments that helped us strengthen the paper in several places.

Reviewer 1, figure comments

- *Figure 2, no units are mentioned or indicated for the x (time) and y (BOLD signal % signal change?) axis.*
- *Figure 5: The panels would be easier to read and interpret if the scales matched, at least within region x tasks.*

Response:

Thank you. Both comments have been addressed and corrected in the main text accordingly.

Reviewer 2

Overall comment of Reviewer 2:
"The study investigates the possibility and the impact of modulating activity in two core areas of the face processing network (FFA, OFA) via NFB. The authors provide evidence that participants are able to modulate the difference in activation between the two areas. Further, this difference impacts activation in the same areas during subsequent detection and recognition tasks as well as the reaction time associated with these tasks. Theoretically, the authors argue that the results support a non-hierarchical model of face processing that relies on the FFA as an entry point and a key area for face detection. Overall, the study makes a significant contribution to the field: the ability to modulate activity in specific nodes of the face network via NFB provides a novel and informative tool for understanding its functional architecture. The manuscript is clearly written and I enjoyed reading it. My comments below aim primarily to enhance clarity and to strengthen the conclusions of the study."

Response:
We are gratified by these thoughtful and encouraging comments, and thank Reviewer 2 for their constructive input. We have carefully addressed the points raised below, which helped us clarify several aspects of our methodology and sharpen the theoretical framing of our conclusions.

Major

Reviewer 2, comment #1
"Activity in the FFA appears robust in the regulation stage even in the control group across both sessions (Fig 2A, G). I wonder what accounts for this: is it an anticipatory response associated with the subsequent face detection task? Also, the difference between the experimental and the control group in the same figure is not easy to interpret since the markers are not labeled with a significance value, there is no mention of multiple-comparison correction, the shaded areas are not labeled as SE/CI etc."

Response:
Thank you for highlighting this. Regarding the robust FFA activity in the control group during the regulation stage. Indeed, participants in the control condition were also instructed to apply face-related mental imagery strategies. This likely explains the globally increased activity in relevant ROIs during regulation, although without ROI selectivity according to training session. Importantly, the control group did not receive real neurofeedback and thus did not differentially modulate the two ROIs, whereas the experimental group receiving real feedback exhibited significantly more effective and targeted regulation outcomes. This point is addressed when we discuss the possible ceiling effect on FFA activity during regulation.

Concerning the clarity of Fig. 2: we have revised the figure and its legend to explicitly indicate the meaning of shaded areas, clarify which comparisons are statistically significant, and address the multiple-comparison correction.

Reviewer 2, comment #2

"Participants were advised to explore face-related visualization strategies to modulate activity. It would be informative to know, even anecdotally, what strategies were found successful in upregulating FFA vs OFA activity."

Response:

During the debriefing sessions, participants were encouraged to share their experiences and strategies were recorded. However, our inspection of these individual reports did not reveal any systematic differences in the strategies used to upregulate FFA versus OFA activity. Participants most commonly described mental visualization techniques, such as imagining faces of familiar individuals, recalling emotional expressions, or picturing detailed facial features. While these strategies were diverse and varied across individuals, no clear pattern emerged to indicate that specific strategies were more effective for modulating one region over the other. We now mention this in the revised paper and hope this clarifies the findings and provides context for the observed results. This remains however an interesting question for future investigation.

Reviewer 2, comment #3

"In the presence of high OFA activity, it seems that detection performance is improved if FFA activity is reduced (Fig. 6c). Could this indicate an alternative part-based strategy for face detection, supported by the OFA, but interfering with a more holistic strategy deployed by the FFA?"

Response:

This is an interesting and insightful observation, as the reciprocal relationship between OFA and FFA activity might indeed suggest the hypothesis of an alternative strategy for face detection mediated by higher OFA activity. While we highlight the very strong evidence that a combination of high FFA and low OFA activity is optimal for fast detection (section 4.4), we acknowledge that the reverse pattern (high OFA activity and low FFA activity) could in principle also facilitate detection and thus reflect an alternative strategy via OFA-mediated part-based processing. However, as shown by the modelling effects depicted in Fig. 6c, this pattern is actually weak and not clearly dissociable across varying levels of OFA activity, hence it is overall far from the strong facilitation associated with high FFA and low OFA activity, which remains preferred and linked to faster face detection times (figure 6c). We now note this point in the text **(p40)**.

Reviewer 2, comment #4

"The NFB session has a direct advantageous effect on detection and recognition performance in the experimental group (Fig. 7b). Somewhat surprisingly though, there is a "benefit in the OFA training session, independent of task-evoked activity" (lines 714-716), running counter to the impact of higher OFA activity during detection. One possibility relies on the contribution of additional regions recruited during regulation. Alternatively, though, I wonder if this does not simply reflect a higher level of motivation during the detection/recognition tasks associated with the successful use of strategies in the experimental (versus control) group. Since the authors collected data on the level of motivation of the participants for each run, this possibility can be easily addressed."

Response:

This is a valid suggestion, and we agree that differences in motivation could, in principle, contribute to the observed behavioural effects. We did analyse the motivation ratings across

groups and sessions, and although some interesting trends emerged (see figure below), none of the differences reached statistical significance. For this reason, and to maintain focus in the main narrative, we did not include them in the manuscript.

Notably, as shown in the figure, the control group even reported slightly higher motivation on average (albeit non-significant), possibly reflecting increased effort in the face of a more challenging or less effective training experience. This makes it unlikely that the behavioural advantages observed in the experimental group were driven by differential motivation.

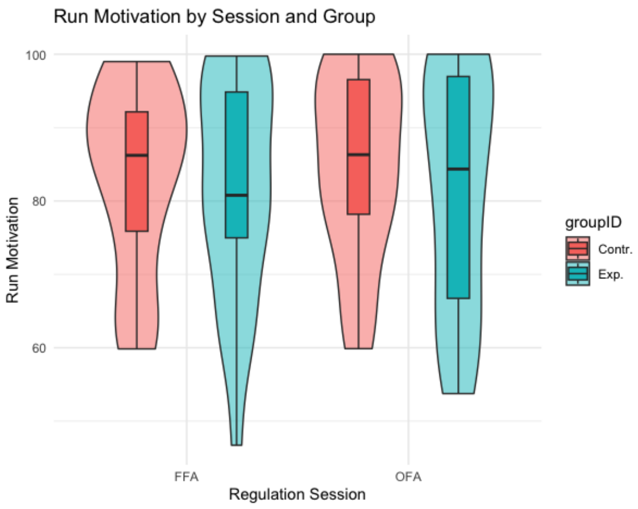

Figure 1 Average Run Motivation by Session and Group

Violin plots showing the distribution of subject-level average motivation ratings during FFA and OFA training sessions for the experimental (EXP) and control (CONT) groups. Each violin reflects the spread of per-subject means within each condition. No significant differences in motivation were observed between groups or sessions.

Reviewer 2, comment #5

"The authors state that "regulating FFA did not change the detection responses in OFA" but Fig. 5b seems to indicate otherwise. Please clarify."

Response:

This is again a valuable point. We agree that Fig. 5b indicates a differential effect of FFA regulation (regFFA) on OFA responses depending on the session - such that during FFA sessions (unlike OFA sessions; p=0.1), regFFA was associated with a relative decrease in OFA activity. Our statement actually referred to a lack of any co-activation or "spill-over" of FFA regulation on OFA, but we realize this was inappropriately phrased.

Indeed, our primary goal with the NFB modulation was to enhance the activity of the target ROI during subsequent task phases. This effect, seen predominantly within the same ROI (e.g., regulating FFA increasing FFA responses during the task), aligns with our design intentions. The observed reduction in OFA detection activity as a function of enhancing FFA regulation activity in the FFA sessions is not inconsistent with this goal; rather it may reflect a dynamic adjustment within the face-processing pathways subsequent to training in favour of FFA. In other words, by prioritizing FFA detection activity through prior up-regulation, a relative downregulation of OFA may occur as part of a functional rebalancing of processing strategy induced by NFB. Therefore, this finding fully accords with the broader conclusion we made: the magnitude of modulation in the targeted ROI (whether FFA or OFA) during NFB was linked to an increase of task-phase responses in the same ROI, reinforcing the role of region-specific modulation in shaping subsequent behavior. The relative decrease in OFA during FFA sessions ultimately aligns well with the intended functional enhancement of the targeted region and provides further insight into reciprocal interactions within the face-processing network.

We appreciate the opportunity to clarify this observation and integrate it with our overall framework, and we therefore modified our wording accordingly **(p27, p28, p38)**.

Our thanks for careful reading and insightful comments.

Minor

Reviewer 2, comment #1
"Were trials with incorrect responses removed? How many?"

Response:
We did not exclude incorrect trials from our analyses to avoid selection bias and preserve statistical power. Please note that accuracy was near ceiling for detection (99.3%) with no speed difference between correct and incorrect trials ($p > .146$), whereas recognition accuracy was 68% and showed a speed-accuracy trade-off with correct responses ~4 frames faster than incorrect ones ($p = .022$). However, excluding incorrect trials would substantially reduce power.

Importantly, re-running our two central behavioral models (LMM models 6 and 7) with only correct trials produced no meaningful changes to our results or interpretations. We have clarified this methodological decision in the manuscript **(p17)**.

Reviewer 2, comment #2
"The authors refer to a Fig. 8 (line 765), which does not appear in the manuscript"

Response:
Thank you and now corrected in manuscript.

Reviewer 3

Global comments of Reviewer 3:
"The authors aimed at dissecting the specific functional role of OFA and FFA in relation to face processing by differentially manipulating the BOLD activity in these two regions using rtfMRI neurofeedback, and by concurrently testing how learned differential regulation of OFA and FFA activity affects consecutive face detection/recognition tasks.

The article is well structured and written in all sections. The objective of the study, whose rational is appropriately introduced and motivated, is neuroscientifically challenging and potentially quite influential."

Response:
Thank you for your positive appraisal of our work and its significance. We appreciate your careful comments and feel that all changes made in response to these points have improved the paper in multiple ways, as we describe in details below.

Major

Reviewer 3, comment #1
"The methodology is clear and sufficiently detailed, however it does not appear particularly innovative and advanced. For instance, a differential feedback between two cortical areas (SMA vs PPA) was already proposed by Weiskopf et al. 2004. In addition, the assessment of the behavioral effects of regulation on a trial-by-trial basis was also previously proposed by Caria et al.2010 where emotional stimulus perception and evaluation was interleaved with consecutive regulation blocks."

Response:
Thank you for raising this point of clarification. Indeed, we are aware that differential feedback between cortical areas and trial-by-trial behavioral assessments have been explored in prior studies. However, differential feedback between two regions was previously implemented to use one region as a control for the target ROI (e.g. Weisskopf et al.) or to induce interhemispheric biases between homologous regions in visual attention (e.g. Robineau et al.). Here the novelty of our approach lies in targeting two functionally linked regions (FFA and OFA) <u>within the same network</u> that subserve <u>the same cognitive function</u>, namely face processing. Even more critically, the relative contribution of each of these two interconnected regions has remained debated. We believe this is conceptually very much unlike the NFB modulation of anatomically distinct and unrelated regions (e.g., SMA vs PPA) used in prior studies. Additionally, our design involved an experimental manipulation of ROI activity on a trial-by-trial basis interleaved with a constant visual task, allowing us to directly asses the quasi-causal role of each ROI in the task – rather than to induce more long-lasting plasticity-related effects as typically used for rehabilitation purposes. Although a trial-by-trial approach itself is not novel (Caria et al., 2010), its application here serves to bridge self-regulation with immediate behavioral outcomes in a network-specific context, or task-specific domain, providing a direct link between neural modulation and behavioural performance.

Therefore, by probing two well-defined regions with shared functional roles across specific face-related tasks, we could demonstrate their complementary contributions to detection and recognition processes and make important theoretical inferences about the underlying network

organization, unlike previous NFB applications. We believe this causal approach to dissect processing steps along a cognitive function through NFB is novel and differs from standard NFB studies aiming at strengthening or attenuating a given ability. Our study also addresses an important theoretical debate in the face processing domain via NFB, rather than studying NFB itself. We hope our revision now makes this novelty more comprehensible.

Reviewer 3, comment #2

"My major objection is that a differential BOLD activation (dominance) in FFA wrt OFA, or viceversa, might be sufficient to clearly dissect the distinct functional role of these regions in face processing. Specific engagement of visual areas during a visual task is not necessarily reflected by an overall increased/decreased ROI activation. For instance Shibata et al 2011 showed that increased visual stimulus detection was associated with a learned control of stimulus-specific brain pattern in V1/V2 resulting in a combination of positive and negative voxels almost symmetrically distributed around 0. Similarly, as FFA and OFA are posited to process face-specific local or global features, highly selective BOLD activity patterns might indeed characterize OFA and FFA activity. This features selective activity, that in fact might be modulated during self-regulation, would then result in either increased, decreased or null mean BOLD activity. However, the interpretation of the potential influence on consecutive face detection/recognition tasks would be then confounded when discussed in terms of a higher or lower activation and not in terms of the actual face-related representational content, that might be highly specific also in case of an overall low or null BOLD activation."

Response:

This is a well taken point. We fully agree that changes in the representational content of face-related activity, such as systematic decreases in a subset of neurons offset by increases in others, could underlie the overall BOLD signal measured in a given ROI without being captured by our approach. We are also aware of the work done by Shibata et al (2011) whose sophisticated methodology could have other potentially interesting applications in the domain of face processing. While our study focused on modulating the overall amplitude of activity within the FFA and OFA, this does not preclude the existence of more nuanced, computationally relevant patterns that could also influence behaviour – in similar or other tasks. Investigating such representational content with a multivoxel pattern-based NFB approach would be an interesting direction for future research.

Here, by design, we focused on the relationship between the overall amplitude of modulation and task performance in line with previous work concerning our research question. Specifically, we were motivated by previous studies linking overall recruitment (e.g. average BOLD signal) of OFA and FFA in distinct aspects of face processing (such as detection and recognition). Accordingly, all conclusions from these studies derived from standard univariate analysis, not from multivoxel pattern analysis. In addition, our findings themselves aligned with this standard approach to fMRI BOLD activity, by demonstrating that both increases and decreases in activity made significant contribution to behavioural performance in a balanced, interactive manner (e.g. as evidenced by the interaction effects on behavioral outcomes). But the results could have been otherwise and thus falsify our hypothesis. Hence, the current data speak for themselves by showing that global increases / decreases in each ROI can be differentially related to performance. However, this does not contradict the possibility of other neural-behavioral relationships as imagined by the Reviewer. Therefore, while we did correlate overall ROI activity with performance, we did not claim that only increases in activity improve performance. Locally, the reviewer's hypothesis about feature-selective patterns remains plausible but beyond the

scope of the present study. We hope our revised manuscript makes this point clearer and we cite the work of Shibata in more details in our revision **(p2)**.

Reviewer 3, comment #3
"In addition, mean BOLD signal magnitude can be also modulated by confounding non-neural factors such as changes of basal vascular physiology, that are region-specific (Kurzawski et al., 2022; Williams et al., 2023). Such a potential variance, that during neurofeedback would play a critical role, is not sufficiently controlled/reduced by a differential feedback between two regions, as these regions might be characterized by different physiological mechanisms."

Response:
We agree that non-neural factors, such as basal vascular physiology, can modulate mean BOLD signal magnitude. However, this issue is inherent to BOLD fMRI methodology in general and applies to all activation studies broadly; hence it is not unique to neurofeedback paradigms. In addition, as noted by this Reviewer in comment No 1 above, it is a common practice in NFB research to control for regulation specificity by using differential BOLD signal relative to a second region outside the target ROI; hence our NFB calculation follows highly standard state-of-the-art methodology. For this reason, our differential feedback signal computed between two adjacent cortical areas is as strict as, or even stricter than, most fMRI NFB studies. Furthermore, although vascular physiology may differ between region and contribute to variance in BOLD response within single individuals, to our knowledge there is no evidence to suggest that vascularization differs significantly between the FFA and OFA, as both regions are located within the extrastriate cortex and are dependent on the posterior cerebral artery for blood supply. These anatomical and vascular similarities reduce the likelihood of region-specific physiological mechanisms confounding our results. Accordingly, in the literature, no systematic vascular difference has been reported to account for different response profiles of these two interconnected areas, despite a vast amount of studies in this field. In addition. It has been shown that between-subject variance within a group is generally higher than between-region variance within a subject, such that random effect analyses in group studies such as ours are robust to these more subtle effects. Finally, we should emphasize that any systematic vascular effect as suspected by the reviewer would affect all conditions alike and seem insufficient to explain our key findings of significant interaction between regulation block, target region, and face task. Hence, we believe there is no fair reason to suspect that vascular effects would confound our results.

Reviewer 3, comment #4
"A second major issue is related to the assumption that the preceding activity affects consecutive face stimuli presentation. Considering the low temporal resolution of the BOLD signal and of the experimental protocol, the persistence of the preceding modulation of FFA or OFA dominance activity during face processing is not certain, and difficult to prove in the presence of a visual stimulus. In fact, in Figure 2, the increased BOLD activity extending into the task phase appears to reflect an initial effect of the previous regulation block and its associated delayed response (about 5 to 6s), and a later increase likely because of the face stimulus presentation. In any case, it would be important to propose a clear explanation of the hypothesized sustained effect in terms of possible long-lasting (reverberating?) neuronal processes elicited by neurofeedback-based regulation."

Response:
We agree that the sluggish nature of the BOLD response during regulation and its overlap with task-phase activity could complicate interpretations about the exact timing of influences exerted by regulation on subsequent face stimulus processing. While it is challenging to fully disentangle

regulation-phase carryover from task-specific activation due to the temporal resolution of fMRI, our experimental design and findings suggest that the preceding neurofeedback (NFB) modulation plays a functional role in task performance beyond its immediate impact on BOLD signal magnitude. In fact, this overlap could even be considered as an advantage and offer direct evidence in favour of an effect of regulation on FFA/OFA activity following regulation and accounting for the modulation of the face task. Furthermore, our detailed statistical analysis and structural equation modelling help tease apart the role of differences in BOLD signals at different time points in such modulation. Finally, please note that the concern of the reviewer might apply to any other traditional neurofeedback study where the effect of brain function or behaviour is tested after variable or longer delays, making it hard to relate the increases in activity due to self-regulation to the subsequent increases in task performance. In fact, few if any studies previously reported a precise analysis of these temporal relationships as we do here.

Regardless, our approach and findings are fully in accord with the possible explanation offered by the reviewer, and we are happy to expand on this point along their interesting hypothesis. To speculate about the possible neural bases of sustained effects, in line with the reviewer, we surmise that the neurofeedback-based regulation may elicit neuronal processes (e.g. changes in synaptic activity or short-term plasticity) that reverberate beyond the immediate regulation phase. These effects could mediate a functional priming of the targeted regions (FFA or OFA), whereby self-regulation enhances the readiness or efficiency of neural computations necessary for face detection or recognition. Such priming effects, while not fully captured by BOLD signal dynamics alone, could underlie the observed behavioral improvements. Please also note that effects of performance were not only region- and task dependent, but also found in the EXP group specifically, not in the CONT group, further arguing for a functionally selective effect of these sustained effects and against non-specific BOLD carry over.

We have now incorporated this discussion into the revised manuscript to better contextualize the observed results and more directly address the reviewer's valuable suggestion **(p37)**.

We also believe that limitations imposed by the slow temporal resolution of fMRI might be usefully illuminated in future studies by employing techniques with higher temporal resolution, such as MEG or EEG, which could provide further insight into the timing **of** neurofeedback-based modulations across successive phases from regulation through to task and baseline periods. However, as explained above, these limitations do not undermine our findings and conclusions.

Reviewer 3, comment #5

"Furthermore, as increased neural activity is inevitably associated with explicit or implicit perception/behavior, a more clear understanding of how mental strategies during regulation affects self-regulation itself as well as the consecutive face detection/recognition tasks is highly desirable (for instance by analyzing more clearly what specific strategies were adopted by the participants), so as to prevent readers from thinking that brain and mind work independently, in particular during self-regulation. This would also be possibly informative of the observed differential ability in modulating FFA and OFA dominance."

Response

We agree that understanding how mental strategies influence self-regulation and subsequent task performance is important. As noted earlier (see response to reviewer 2, point 1), we collected reports of strategies during the debriefing sessions but our inspection of these individual reports did not reveal any systematic differences in the strategies used to upregulate FFA versus OFA activity. Although participants commonly described face-related visualization

techniques, such as imagining familiar faces or focusing on facial features, no systematic differences in strategies were identified that could explain the differential modulation of FFA and OFA activity. It is however a classic observation in NFB research that participants often have no direct phenomenal access to the exact mental process underlying successful regulation, and participants in sham groups often report a subjective experience of control despite the lack of veridical NFB signal. These observations are intriguing and definitely beg for more research

More generally, we do not imply that the brain and mind work independently during self-regulation and not do fully understand why the reviewer thought our work implied such a statement. Neurofeedback inherently integrates both explicit and implicit processes, with implicit mechanisms such as reinforcement learning often playing a significant role (Lubianiker et al., 2022). These processes may operate below conscious awareness, shaping neural activity and influencing performance without the participant explicitly understanding how their strategies contribute to regulation (Sitaram, 2017). In fact, this is often put forward as an explanation for poor introspective access to NFB success. In any case, to avoid ambiguity, we now note the likely interplay of both explicit and implicit processes in the revised manuscript, making it clear that both may contribute to self-regulation and behavioral outcomes. Additionally, we emphasize how participant-reported strategies, while informative, represent only part of the broader neurofeedback mechanism **(p21)**. Thank you for prompting us to clarify this issue.

Reviewer 3, comment #6

"Finally, the authors did not test whether the additional brain regions involved in self-regulation (paragraph 3.1.3) also explain (predict) the observed changes in face detection/recognition. Such a control is important to demonstrate the specificity of the ROIs-induced effects."

Response:

This is a well taken point indeed. We have rerun the analysis to include another region from the face network, namely, the superior temporal sulcus (STS), defined based on the localizer data. STS was co-activated but not targeted by neurofeedback. We now added a similar analysis as those conducted on FFA and OFA to examine its role in predicting the observed changes in face detection and recognition **(Suppl. Mat. Fig 7 in section Roi Specificity)**. A reference to these results is now included in the revised manuscript to address the specificity of the ROI-induced effects **(p38).**

Briefly, although STS was engaged during the task and showed a general relationship with face detection performance, its activation was not differentially modulated by neurofeedback training (no STS × Regulation effects) and did not account for the behavioral changes associated with FFA and OFA regulation activity. Critically, all key effects involving the targeted ROIs remained significant when STS activity was included as a covariate. These findings converge with the indea that the observed behavioral modulation arises from (neurofeedback-induced) changes in FFA and OFA, rather than broader co-activation within the face-processing network.

Additional points

Additional point 1:
"*page 17 lines 464/472 Description of LLMs for detection and recognition should be improved as it is currently not clear. The authors refer to recognition models but not to detection models. Lines 471/472 p 17. as the models mentioned in the paragraph "Effects of Self-Regulation Performance on ROI Detection and Recognition Responses" are 4 what are the numbers 3 and 5 referring to? I suppose that model 1 refers to NFB learning mentioned in the previous paragraph whereas models 2 to 5 refer to those mentioned in the current paragraph, but that's not evident."*

Response:
Noted and corrected in the manuscript **(p17)**.

Additional point 2:
"*page 17 lines 474/476, the author state that "a positive association between regFFA and FFA detection activity would suggest that the FFA during self-regulation predicts subsequent enhanced FFA detection activity." This is a rather speculative assumption as a positive association might be also result from increased activity associated with face-related imagery during regulation and face processing during face detection task, without implying any predictive/causal dependency."*

Response:
Our use of the term *"predict"* was not intended to imply causality, but rather to indicate a statistical association with temporal precedence (regulation-phase activity occurred prior to detection-phase activity) and was used as a predictor variable in the model. Nevertheless, we understand how this phrasing may be misinterpreted, and have revised the sentence to clarify this point **(p18)**.
